# Cysteine imaging reveals early redox dysregulation and identifies gnetol as a ferroptosis-modulating agent in doxorubicin cardiotoxicity

**DOI:** 10.1016/j.redox.2026.104131

**Published:** 2026-03-21

**Authors:** Yan Chen, Bo Zhang, Yufan Wei, Yanfa Dai, Baoyue Zhang, Jing Li, Ke-Jia Wu, Ning Sun, Chenwen Shao

**Affiliations:** a Wuxi School of Medicine, Jiangnan University, Wuxi, Jiangsu, 214122, China; b School of Pharmacy, Changzhou University, Changzhou, 213164, China; c MOE Medical Basic Research Innovation Center for Gut Microbiota and Chronic Diseases, Wuxi School of Medicine, Jiangnan university, Wuxi, Jiangsu, 214122, China

**Keywords:** Doxorubicin cardiotoxicity, Redox imaging, Cysteine, Ferroptosis, Hepcidin-FPN1, Gnetol

## Abstract

Doxorubicin (DOX)-induced cardiotoxicity remains a major limitation of cancer chemotherapy, largely due to the lack of sensitive approaches for early detection and effective cardioprotective interventions. This study investigated whether cysteine depletion represents an early redox event during DOX cardiotoxicity and evaluated a cysteine-activatable fluorescent probe, termed the cardiotoxicity-responsive cysteine probe (CCP), for *in vivo* redox imaging and therapeutic discovery. Cardiac imaging revealed a significant reduction in intracellular cysteine levels three weeks after DOX administration, preceding systolic dysfunction detected by echocardiography at four weeks. Mechanistically, cysteine depletion was accompanied by impaired glutathione-dependent antioxidant defense, iron accumulation, lipid peroxidation, and ferroptosis. Through probe-guided screening, gnetol (a naturally occurring polyphenolic stilbene) was identified as a potent regulator of intracellular cysteine homeostasis. Gnetol restored cysteine and glutathione levels, reduced lipid peroxidation, and suppressed ferroptosis by modulating the SMAD–hepcidin–FPN1 axis and preserving glutathione peroxidase 4 activity. In the mouse model of DOX-induced cardiomyopathy, gnetol significantly improved cardiac function, attenuated myocardial injury and fibrosis, and reduced oxidative stress without evident systemic toxicity. Collectively, these findings establish cysteine depletion as an early redox feature of DOX cardiotoxicity and demonstrate that cysteine-targeted redox imaging enables mechanism-guided discovery of cardioprotective agents. This study highlights gnetol as a promising ferroptosis-suppressing candidate and provides a mechanistic framework for early detection and intervention in redox-driven cardiac injury.

## Highlights

• Cardiac cysteine depletion occurs early in doxorubicin cardiotoxicity.
• Cysteine loss precedes systolic dysfunction in a mouse model.
• Imaging-guided screening identifies gnetol as a cysteine homeostasis modulator.
• Gnetol attenuates ferroptosis via SMAD–hepcidin–FPN1 and GPX4 pathways.

## Graphical abstract

**Figure:**
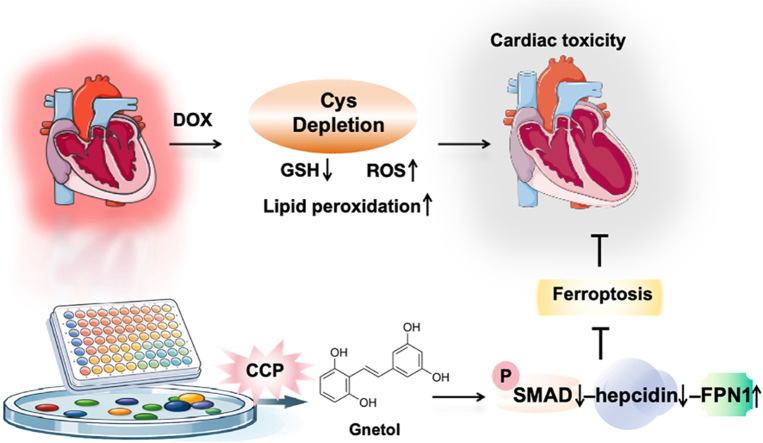

## 1 Introduction

Doxorubicin (DOX) is widely used to treat hematologic malignancies and solid tumors, but its clinical utility is limited by dose-dependent and often progressive cardiotoxicity [[1], [2], [3]]. Despite advances in surveillance and cardioprotective strategies, anthracycline-induced cardiomyopathy remains a major cause of long-term morbidity in cancer survivors [[4], [5], [6]]. Clinically, cardiac injury is frequently recognized only after measurable declines in systolic function, such as reductions in ejection fraction, at which point myocardial damage may be partially irreversible [4,5,7]. Therefore, identifying early molecular changes that precede overt functional deterioration is essential for improving risk stratification and enabling timely intervention [4,7,8].

Oxidative stress and dysregulated iron metabolism play central roles in DOX cardiotoxicity [5,9,10]. Ferroptosis, an iron-dependent form of regulated cell death driven by lipid peroxidation, has been increasingly implicated in cardiomyocyte injury under conditions of oxidative stress [[11], [12], [13]]. Cysteine is the rate-limiting substrate for glutathione (GSH) synthesis, and its availability regulates the System Xc^−^/GSH/GPX4 axis, a core pathway for ferroptosis defense. [12,14,15]. These observations suggest that loss of cysteine may occur early during DOX-induced cardiac injury and contribute to subsequent ferroptotic damage.

A major challenge in addressing this possibility is the limited ability to monitor intracellular cysteine dynamics in cardiomyocytes and in the intact heart in real time, especially in living systems where early molecular alterations may arise before detectable functional or structural changes. Fluorescent probes that can track cysteine changes offer a good solution [16,17]. They allow scientists to watch and measure molecular changes in cells and *in vivo* [[18], [19], [20], [21], [22]]. These probes can be used not only to discover biomarkers but also to test potential treatments, directly linking early molecular changes to possible interventions.

In this study, we report a fluorescent probe called CCP. CCP can monitor cysteine levels in a sensitive and selective way. Using CCP, we identify intracellular cysteine depletion as an early molecular signature of DOX-induced cardiotoxicity (DIC) that precedes echocardiographic dysfunction in a mouse model. We further establish a CCP-guided screening strategy to discover small molecules that restore intracellular cysteine under DOX stress, leading to the identification of gnetol as a cardioprotective candidate. Finally, we delineate a mechanistic framework linking cysteine restoration to suppression of myocardial ferroptotic injury, highlighting the SMAD-hepcidin-FPN1 axis and the glutathione–GPX4 antioxidant system as convergent targets. Together, this work provides a probe-enabled mechanistic framework for early detection and mechanism-informed intervention in anthracycline-induced cardiotoxicity.

## 2 Materials and Methods

### 2.1 Materials and instruments

Chromatographic purification was performed with 300-400 mesh chromatography silica gel (Haiyang, Qingdao, P.R. China). The NMR spectra were recorded in DMSO‑*d*_6_ on DRX-600 and Advance III HD spectrometer (Bruker, Rhenistetten-Forchheim, Germany). The mass spectra were evaluated on AB SCIEX Triple-TOF 4600. The UV–vis absorption spectra of samples were carried out on a UV-2450 spectrophotometer (Shimadzu, Japan). All fluorescence measures were recorded on by Hitachi fluorescence spectrometer F-7000. The pH values were determined on the PHS-25 pH-meter (Shanghai Geotechnical International Trading Co. Ltd., Shanghai, P.R. China). The cell imaging was analyzed on a confocal laser scanning microscope (CLSM) (Lei TCS SP8-MaiTai MP, Germany). The fluorescence imaging experiments of animals were detected on an IVIS Lumina XR multispectral imaging system (PerkinElmer, USA).

### 2.2 Synthesis of compound CCP-OH

3-Hydroxy-3-methylbutan-2-one (449 mg, 4.40 mmol) and malononitrile (594.5 mg, 9.00 mmol) were dissolved in anhydrous ethanol (10 mL). Sodium ethoxide (45 mg, 0.66 mmol) was added portionwise under stirring, and the mixture was refluxed for 2 h. After removal of the solvent under reduced pressure, the crude product was obtained and used for the next step (or purified if needed) to afford compound 1 (∼621 mg, 75%) [23].

Next, compound 1 (188.2 mg, 1.00 mmol) and 2-bromo-4-hydroxybenzaldehyde (402.02 mg, 2.00 mmol) were dissolved in ethanol (15 mL), followed by addition of piperidine (28.1 mg, 0.33 mmol). The reaction mixture was refluxed for 12 h until the reaction completed by TLC. After the reaction solvent was removed, the resulting crude product was purified by column chromatography (PE/EA = 6:1) to obtain CCP-OH a solid (∼191 mg, 50% yield).

### 2.3 Synthesis of probe CCP

Acryloyl chloride (90.51 mg, 1.00 mmol) and CCP-OH (305.78 mg, 0.80 mmol) were dissolved in anhydrous dichloromethane. Then the reaction solution was added 5 drops of triethylamine (TEA) as catalyzer and stirred overnight at 0 °C. Finally, the solvent was removed and then the mixture was purified via column chromatography using petroleum ether and ethyl acetate (V: V = 8:1) to acquire probe CCP (∼227 mg, 63% yield).

### 2.4 Spectra measurement of CCP

The stock solution of the probe CCP dissolved in DMSO at a concentration of 1.0 mM for further use. The stock solution of hydrazine, amino acids, cations, and anions was prepared in ultrapure water. All spectroscopic records were performed at room temperature in PBS buffer solution. The wavelength of excitation was set as 560 nm. Both excitation and emission slit widths were 5 nm. The photomultiplier voltage was 600 V. The error bars were the standard deviation based on three independent experiments.

### 2.5 Cell viability assay

H9C2 cells were cultured in Dulbecco's Modified Eagle Medium (DMEM) supplemented with 10% fetal bovine serum (FBS) and 1% penicillin/streptomycin (P/S). The cells were maintained in a humidified incubator at 37 °C with 5% CO_2_. Doxorubicin hydrochloride (DOX, MCE, HY-15142), Gnetol (TCI, #G4359), and Dexrazoxane (DEX, Aladdin, #D134376) were dissolved in dimethyl sulfoxide (DMSO) as stock solutions and subsequently diluted with the complete culture medium to the desired working concentrations. For the cell viability assay, cells were seeded into 96-well plates and allowed to adhere for 24 h. Following attachment, the cells were treated with the specified compounds at predetermined concentrations for 24 h. After the treatment period, the supernatant was removed, and each well was replaced with 100 μL of fresh complete medium containing 10% (v/v) CCK-8 reagent. The plate was then incubated at 37 °C for 1-2 h. At the end of the incubation, absorbance at 450 nm was measured by using a microplate reader. The absorbance of the control group was set to 1.0 as a reference. The relative cell viability for the treated groups was calculated based on control. Absorbance values were measured from 4 independent culture wells, with each well representing one biological replicate.

### 2.6 The Fe^2+^ levels

H9C2 cells were seeded in 96-well plates and allowed to adhere for 24 h in complete medium. The adherent cells were then assigned to the following treatment groups for 24 h: control (culture medium only), DOX (1 μM), Gnetol (5 μM) + DOX (1 μM), and Gnetol (10 μM) + DOX (1 μM). Following treatment, the supernatant was aspirated, and the cells were loaded with 10 μM FeP in fresh complete medium for 30 min at 37 °C. After incubation, the FeP solution was removed, and the cells were washed with 100 μL of PBS. Fluorescence was immediately measured using a microplate reader with an excitation wavelength of 465 nm and an emission wavelength of 640 nm [24]. The mean fluorescence intensity of the control group was set to 1.0, and the relative fold change in intracellular labile iron for each treatment group was calculated accordingly. Fluorescence intensity were measured from 6 independent culture wells, with each well representing one biological replicate.

### 2.7 Confocal fluorescence imaging experiments

H9C2 cells were seeded onto cell crawls placed in 12-well plates and cultured in DMEM supplemented with 10% FBS and 1% P/S for 24 h to allow adhesion. Following this, the cells were subjected to various drug treatments for 24 h. After treatment, the cells were washed three times with PBS and then incubated with complete medium containing 10 μM CCP at 37 °C for 30 min. Subsequently, the cells were washed again with PBS to remove excess probe. Imaging was performed using a confocal fluorescence microscope (Carl Zeiss LSM88) with an excitation wavelength of 565 nm and an emission wavelength of 615 nm. Fluorescence intensity was quantified in ImageJ from 3 independent wells per group (representing n = 3 biological replicates). For each well, ≥2 random fields of view were analyzed and averaged to yield one value per well (≥6 fields per group).

### 2.8 The lipid peroxidation levels

The level of lipid peroxidation was assessed using the fluorescent probe C11-BODIPY 581/591 and flow cytometry [25]. H9C2 cells were seeded in 6-well plates and cultured in complete DMEM for 24 h. The adherent cells were then treated for 24 h with the following: culture medium (control), DOX (1 μM), a combination of Gnetol (5 μM) and DOX (1 μM), a combination of Gnetol (10 μM) and DOX (1 μM), or a combination of DEX (100 μM) and DOX (1 μM). After treatment, the cells were washed three times with PBS and stained with 5 μM C11-BODIPY 581/591 at 37 °C for 30 min. The cells were then analyzed immediately using a flow cytometer (BD LSRFortessa). The oxidized form of C11-BODIPY 581/591 was detected in the FL1 channel. Three independent culture wells (n = 3 biological replicates) were analyzed by flow cytometry.

### 2.9 Cellular ROS assay

Intracellular reactive oxygen species (ROS) levels were measured using the Reactive Oxygen Species Assay Kit (S0033 M, Beyotime) [26] and CM-H_2_DCFDA (C6827, ThermoFisher) following the manufacturer's protocol. In brief, H9C2 cells were seeded in 6-well plates and cultured in complete DMEM for 24 h. The adherent cells were then treated for 24 h with the following, culture medium (control), DOX (1 μM), a combination of Gnetol (5 μM) and DOX (1 μM), a combination of Gnetol (10 μM) and DOX (1 μM), or a combination of DEX (100 μM) and DOX (1 μM).

For flow cytometric ROS measurement, cells from 3 independent culture wells per group (n = 3 biological replicates) were washed three times with PBS and incubated with 10 μM DCFH-DA (Reactive Oxygen Species Assay Kit, Beyotime) at 37 °C for 30 min in the dark. Cells were then harvested, and the fluorescence intensity of oxidized DCF was immediately analyzed using a BD LSRFortessa flow cytometer.

For fluorescence imaging, cells from 3 independent culture wells per group (n = 3 biological replicates) were washed three times with PBS and incubated with 10 μM DCFH-DA or 5 μM CM-H_2_DCFDA at 37 °C for 30 min in the dark. After washing with PBS, fluorescence images were acquired using a fluorescence microscope (ECHO RVL2-K) under identical exposure settings for all groups, and fluorescence intensity was quantified using ImageJ software.

### 2.10 Mitochondrial membrane potential effects

The effect of Gnetol and DOX on mitochondrial membrane potential (ΔΨm) was assessed using the JC-1 fluorescent probe [27]. Cells were seeded on glass cell crawls in 24-well plates and cultured in complete DMEM for 24 h. The adherent cells were then treated for 24 h with the following: culture medium (control), DOX (1 μM), a combination of Gnetol (5 μM) and DOX (1 μM), a combination of Gnetol (10 μM) and DOX (1 μM), or a combination of DEX (100 μM) and DOX (1 μM). After treatment, the cells were washed three times with PBS and stained with the JC-1 working solution for 30 min at 37 °C in the dark. The stained cells were then imaged using a Zeiss confocal microscope. JC-1 aggregates (red fluorescence, indicating high ΔΨm) and monomers (green fluorescence, indicating low ΔΨm) were detected using the FL-2 (Ex/Em: 488/550-600 nm) and FL-1 (Ex/Em: 488/500-560 nm) channels, respectively. Fluorescence intensity was quantified using ImageJ from 3 independent culture wells per group (n = 3 biological replicates), with 5 randomly selected fields of view per well (15 fields per group in total).

### 2.11 DIC models and treatment *in vivo*

Adult male C57BL/6 mice (6 weeks old, 20-25 g) were obtained from ChangZhou Cavens Laboratory Animal Co. LTD. All animals were housed under standard conditions (temperature: 20-26 °C; humidity: 50-60 %; 12/12 h light/dark cycle) with free access to food and water. All experimental procedures were approved by the Experimental Animal Ethics Committee of Wuxi Medical College of Jiangnan University (Permission Code: JN.No20241115c0450530[596]) and conducted in accordance with the National Institute of Laboratory Animal Health guidelines. For animal experiments, we have specified n = 6-8 mice per group, which was influenced by the mortality associated with doxorubicin-induced cardiomyopathy.

Mice were established as a model of chronic cardiac insufficiency by **i.p.** injection of DOX (5 mg/kg) on days 1, 8, 15, and 22, respectively [[28], [29], [30]]. To evaluate the protective effect of Gnetol, mice were pretreated via intraperitoneal (i.p.) injection with either vehicle (10% DMSO in PBS), Gnetol (3.75 mg/kg/day), or Gnetol (7.5 mg/kg/day) 2 h prior to the first DOX injection. This pretreatment regimen was continued daily for a total of 28 days. Gnetol was first dissolved in DMSO to prepare a stock solution, which was then diluted to the working concentration using sterile PBS containing 10% DMSO for intraperitoneal injection, ensuring the final DMSO concentration did not exceed 10% of the injection volume. Control mice received an equal volume of vehicle (PBS with 10% DMSO).

Cardiac function was assessed by transthoracic echocardiography one day before the initiation of treatments and then weekly thereafter. For each session, the chest hair was removed, and ultrasound gel was applied as a coupling agent. Cardiac images were acquired using the Vevo3100 High-Resolution In Vivo Imaging System. Both B-mode and M-mode images were recorded along the long and short axes of the heart for subsequent analysis.

To minimize bias, randomization and blinding were implemented throughout the study. Specifically, the operators performing the echocardiography, modeling, and data analysis were blinded to the group assignments.

### 2.12 *In vivo* fluorescence imaging in C57BL/6J mice

The control group and the experimental group (DIC model) mouses were intravenously injected with the probe CCP (1.5 mg/kg, 50 μL with 10% DMSO and 20% PEG300 in saline solution) for 30 min respectively. To confirm the response of the probe to Cys *in vivo* mice, the fluorescent signals after the injection were detected on an IVIS Lumina XR multispectral.

Mice were assigned to three groups: control, NEM, and NEM + Cys. Mice in the NEM group received a subcutaneous injection of NEM (1 mM, 50 μL) into the hindlimb, followed 30 min later by an injection of CCP (1.5 mg/kg, 50 μL). In the NEM + Cys group, mice received a subcutaneous injection of NEM (1 mM, 50 μL) into the hindlimb, followed 30 min later by an injection of Cys (1 mM, 50 μL) and then CCP (1.5 mg/kg, 50 μL). To confirm the response of the probe to Cys *in vivo*, fluorescence signals were detected using an IVIS Lumina XR multispectral imaging system.

*In vivo* fluorescence imaging was performed using an IVIS Lumina XR multispectral imaging system with the following settings: excitation filter, 570 nm; emission filter, 620 nm. Exposure time was automatically adjusted, but all images were normalized to exposure time for quantitative comparison. Fluorescence signals were quantified as total radiant efficiency (p/s)/(μW/cm^2^). Regions of interest (ROIs) were consistently defined as the cardiac projection area with constant size across all animals.

### 2.13 HE and masson

Heart tissues were fixed in 4% paraformaldehyde for 24 h, followed by paraffin embedding. Subsequently, the embedded tissues were sectioned at a thickness of 5 μM. The sections were then subjected to hematoxylin and eosin (H&E; G1076, ServiceBio) and Masson's trichrome (G1006, ServiceBio) staining according to the manufacturer's standard protocols.

### 2.14 Immunofluorescence and immunohistochemical staining

Mice were deeply anesthetized with isoflurane and euthanized by decapitation, followed by immediate transcardial perfusion with 4% paraformaldehyde (PFA) to clear residual blood from myocardial tissue. Hearts were promptly collected and fixed in 4% PFA overnight at 4 °C. Following fixation, tissues were dehydrated, cleared, and embedded in paraffin. Sections were cut at 5 μM thickness and mounted for subsequent staining.

For immunofluorescence staining, sections were deparaffinized and rehydrated through a graded ethanol series. Antigen retrieval was performed, followed by blocking with 5% goat serum for 1 h at room temperature. Sections were then incubated overnight at 4 °C with primary antibodies against GPX4 (Proteintech, 67763-1-Ig), FPN (Proteintech, 26601-1-AP), or 4-HNE (MCE, HY-PB1208). After washing, appropriate Alexa Fluor 488 or 594 conjugated secondary antibodies were applied for 1 h at room temperature. Images were acquired using a fluorescence microscope with a 20 × objective, and fluorescence intensity was quantified using ImageJ software.

For immunohistochemical detection of HEPCIDIN (HAMP), 5 μM sections underwent antigen retrieval in citric acid buffer, endogenous peroxidase quenching with 3% hydrogen peroxide, and blocking with 10% goat serum. Sections were incubated with anti-HAMP antibody (Affinity, DF6492) overnight at 4 °C, followed by incubation with EnVision™+/HRP reagent (ProteinTech, PK10006) for 1 h at 37 °C. Signal was developed using diaminobenzidine substrate at room temperature.

### 2.15 WGA fluorescence staining

To analyze the cross-sectional area of cardiomyocytes, paraffin-embedded heart sections were deparaffinized and rehydrated through a graded ethanol series. Antigen retrieval was performed by microwave heating in EDTA buffer (pH 6.0). The sections were heated until boiling and maintained for 8 min, followed by a 7-min medium-power heating cycle, ensuring the buffer did not evaporate completely. After cooling, the sections were blocked with 5% goat serum for 1 h at room temperature and then incubated overnight at 4 °C with FITC-conjugated wheat germ agglutinin (WGA, 10 μg/mL) to outline cell membranes. Nuclei were counterstained with DAPI (5 μg/mL) for 10 min at room temperature. Finally, the sections were mounted with an anti-fade mounting medium. Images were acquired using a fluorescence microscope with a 40 × objective, and the cross-sectional area of cardiomyocytes was quantified using ImageJ software.

### 2.16 Western blotting

Protein extraction was performed from left ventricular tissues (≈30 mg) and H9C2 cardiomyocytes using RIPA lysis buffer supplemented with 1 mM PMSF and a phosphatase inhibitor. The lysates were centrifuged at 12,000×*g* for 15 min at 4 °C, and the supernatant was collected. Protein concentration was determined using a BCA protein assay kit. Equal amounts of protein (30 μg per lane) were separated by SDS-PAGE on 4–20% gradient gels and subsequently transferred onto polyvinylidene fluoride (PVDF) membranes. The membranes were blocked with 5% non-fat milk in TBST for 1 h at room temperature and then incubated with specific primary antibodies overnight at 4 °C. After washing, the membranes were incubated with an HRP-conjugated goat anti-rabbit IgG secondary antibody (1:10,000) for 2 h at room temperature. Protein bands were visualized using an ECL chemiluminescent reagent and imaged with a GE ImageQuant™ 800 system. Band intensity was quantified using ImageJ software.

### 2.17 Real-time quantitative PCR

Total RNA was extracted from mouse left ventricular tissues (20 mg) using Trizol reagent. Briefly, tissues were homogenized in 1 mL of Trizol using a tissue homogenizer and centrifuged at 12,000×*g* for 5 min at 4 °C. The resulting supernatant was mixed with 200 μL of chloroform, vigorously shaken, and centrifuged at 12,000×*g* for 15 min at 4 °C. The upper aqueous phase was transferred to a new tube, and RNA was precipitated by adding an equal volume of isopropanol, followed by centrifugation at 12,000×*g* for 15 min at 4 °C. The RNA pellet was washed once with 1 mL of 75% ethanol, air-dried, and resuspended in 50 μL of DEPC-treated water. RNA was dissolved by incubation in a metal bath at 55 °C. RNA concentration was determined spectrophotometrically, and the samples were subsequently reverse-transcribed into cDNA using the First-Strand cDNA Reverse Transcription Hypermix Kit.

### 2.18 Statistical analysis

All data were analyzed using GraphPad Prism. N indicates independent biological replicates (independent culture wells for *in vitro* assays or individual mice for *in vivo* studies), as stated in figure legends. For microscopy-based quantification, multiple random fields were measured and averaged to yield one value per biological replicate. Mice were randomized into groups, and investigators performing echocardiography, sample processing, and quantification were blinded to group allocation until analysis was completed. Values of protein/mRNA expression and cell-based readouts were normalized to the blank control group when indicated. Comparisons between two groups were performed using an unpaired two-tailed Student's t-test. For experiments with one factor (≥3 groups), one-way ANOVA with Bonferroni's post hoc test was used. For longitudinal measurements, two-way repeated-measures ANOVA with Bonferroni's multiple-comparisons test was applied. Data are presented as mean ± SD, P < 0.05 was considered statistically significant.

## 3 Results

### 3.1 Design, synthesis, and photophysical characterization of the cysteine-responsive fluorescent probe CCP

**Fig. 1:**
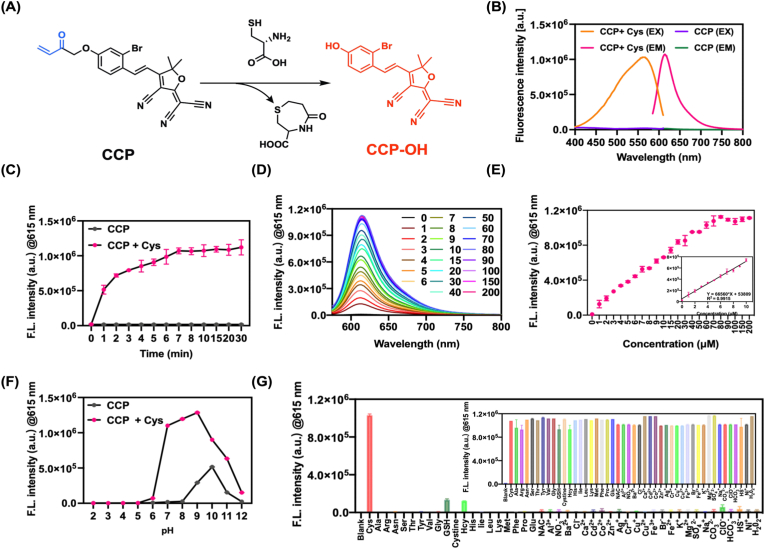
Design, synthesis, and photophysical characterization of the cysteine-responsive fluorescent probe CCP. (A) Chemical structures of CCP and its cysteine-activated product CCP-OH, illustrating the thiol–Michael addition and cleavage mechanism responsible for fluorescence activation. (B) Absorption and fluorescence emission spectra of CCP (10 μM) in the absence or presence of cysteine (100 μM). (C) Time-dependent fluorescence response of CCP (10 μM) upon addition of cysteine (100 μM). (D) Fluorescence emission spectra of CCP (10 μM) in the presence of increasing concentrations of cysteine (0–200 μM). (E) Linear relationship between fluorescence intensity at 615 nm and cysteine concentration. (F) Fluorescence response of CCP-OH under different pH conditions. (G) Selectivity of CCP toward cysteine over other biologically relevant analytes, including biothiols, amino acids, reactive oxygen species, and metal ions. All measurements were performed in PBS buffer (10 mM, pH 7.4, 1% DMSO) at 37 °C. Data are presented as mean ± SD (n = 3).

To visualize intracellular cysteine dynamics during DOX-induced cardiotoxic injury, we designed a cardiotoxicity-responsive cysteine fluorescent probe (CCP). CCP incorporates an acryloyl-based electrophilic recognition unit that undergoes a selective thiol–Michael addition followed by spontaneous cleavage upon reaction with Cys, thereby releasing the masked fluorophore and generating the highly emissive product CCP-OH (Fig. 1A, Scheme S1). This reaction mechanism confers preferential responsiveness toward cysteinewhile minimizing interference from other intracellular nucleophiles. The synthetic route of CCP is outlined in Scheme S1, and the chemical structure of CCP-OH and CCP was fully characterized by ^1^H NMR, ^13^C NMR, and high-resolution mass spectrometry (HRMS), confirming the successful synthesis of the probe (Fig. S1–S6).

The basic photophysical properties of CCP were then characterized. In the presence of Cys, CCP exhibited a maximal absorption peak at 560 nm and an emission peak at 615 nm (Fig. 1B). The reaction process between the CCP and cysteine proceeded rapidly with fluorescence reaching saturation within approximately 10 min, supporting its suitability for real-time monitoring of the changes in cysteine concentration (Fig. 1C). As the concentration of cysteine increased, the fluorescence of CCP also linearly brightened, covering the physiologically relevant range (0-200 μM) [31], with a detection limit of 36 nM (Fig. 1D and E). The high sensitivity of CCP enables detection of subtle changes in intracellular cysteine levels, even when cysteine is depleted during ferroptosis [14]. Moreover, we found that CCP showed good stability in the normal physiological pH range (pH 6.0–8.0) (Fig. 1F). In addition, CCP showed minimal fluorescence decay over 48 h both before and after reaction with cysteine and is suitable for long-term monitoring (Fig. S7). Selective and competitive studies revealed that CCP responded specifically to Cys, while other biothiols (homocysteine and glutathione), amino acids, reactive oxygen species, and metal ions induced negligible fluorescence changes (Fig. 1G). Together, these results demonstrate that CCP is a sensitive and specific probe that can be applied to the monitoring of cysteinein complex biological systems.

### 3.2 Validation of CCP for intracellular cysteine imaging and early *in vivo* assessment of DOX-induced cardiotoxicity

**Fig. 2:**
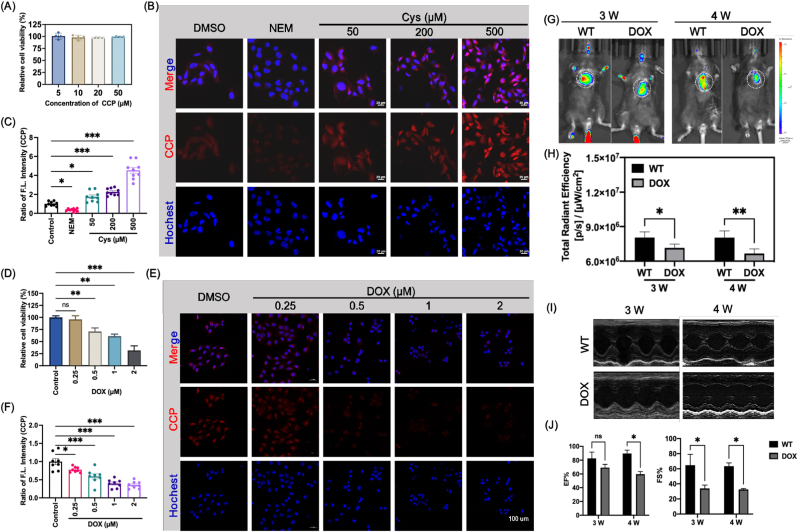
Validation of CCP for intracellular cysteine imaging and early *in vivo* detection of doxorubicin-induced cardiotoxicity. (A) Cell viability of H9C2 cells after incubation with increasing concentrations of CCP, assessed by CCK-8 assay (n = 4). (B) Confocal fluorescence images of H9C2 cells stained with CCP under basal conditions, after thiol depletion with NEM, or after supplementation with exogenous Cys. (C) Quantification of intracellular CCP fluorescence intensity corresponding to (B) (n = 3). (D) Cell viability of H9C2 cells treated with increasing concentrations of DOX (n = 4). (E) Confocal fluorescence images of H9C2 cells stained with CCP after DOX treatment. (F) Quantitative analysis of CCP fluorescence intensity corresponding to (E) (n = 3). (G) *In vivo* fluorescence imaging of mice following tail-vein injection of CCP (1.5 mg/kg) at 30 min post-injection. (H) Quantification of cardiac fluorescence signals shown in (G) (n = 3). (I) Representative M-mode echocardiographic images of control and DOX-treated mice. (J) Quantitative analysis of left ventricular ejection fraction (EF) and fractional shortening (FS) (n = 3). Scale bars: 20 μm. Data are presented as mean ± SD. ∗P < 0.05, ∗∗P < 0.01,∗∗∗P < 0.001.

The ability of CCP to image intracellular cysteine was evaluated in live rat cardiomyoblast H9C2 cells. No cytotoxicity was observed at concentrations up to 100 μM, as measured by CCK8 assay, indicating good cellular biocompatibility of CCP(Fig. 2A). CCP produced a weak basal fluorescence signal consistent with endogenous intracellular cysteinelevels. Treatment with N-ethylmaleimide (NEM, 10 mM), which chemically blocks the intracellular sulfhydryl groups, significantly reduced CCP fluorescence [32], whereas supplementation with exogenous cysteine(50, 200, and 500 μM) resulted in a concentration-dependent fluorescence enhancement (Fig. 2B and C), confirming the responsiveness of CCP to intracellular cysteine fluctuations. To assess the subcellular distribution of CCP, confocal co-localization with organelle trackers was performed (Fig. S8). CCP showed overlap with ER and mitochondrial markers (Pearson's Rr = 0.889 and 0.817, respectively), but only moderate co-localization with the lysosomal marker, indicating preferential enrichment in the ER/mitochondria where redox/thiol homeostasis is tightly regulated.

A DOX-induced cardiotoxicity model was then established in H9C2 cells. DOX treatment (0.25, 0.5, 1 and 2 μM) caused a dose-dependent reduction in cell viability (Fig. 2D), and the fluorescence intensity of CCP also showed a gradually decreasing trend (Fig. 2E and F). These observations indicate that DOX treatment is accompanied by a reduction in intracellular cysteine levels. Notably, the reduction in intracellular cysteine preceded the loss of cell viability, suggesting that cysteine depletion represents an early event during DOX-induced cardiomyocyte injury.

We next examined whether CCP could be applied for early *in vivo* assessment of cardiotoxicity using a mouse model of DOX-induced cardiomyopathy. Following intravenous administration of CCP (1.5 mg/kg) for 30 min, *in vivo* fluorescence imaging was performed. A significant reduction in cardiac fluorescence was observed in DOX-treated mice as early as 3 weeks, with a further decline at 4 weeks (Fig. 2G and H). Notably, echocardiographic assessment revealed that ventricular dilatation and left ventricular ejection fraction (EF) showed no significant changes at 3 weeks but were markedly impaired at 4 weeks. (Fig. 2I and J). Thus, the decline in CCP fluorescence clearly preceded detectable functional impairment, supporting intracellular cysteine depletion as an early and functionally relevant event during DOX-induced cardiotoxicity. To verify that the *in vivo* fluorescence signal originates from biothiol-dependent activation of CCP, mice were pretreated with NEM and then rescued with exogenous Cys, which abolished and restored the hindlimb fluorescence signal, respectively (Fig. S9).

### 3.3 CCP-based screening identifies gnetol as an effective modulator of intracellular cysteine levels

**Fig. 3:**
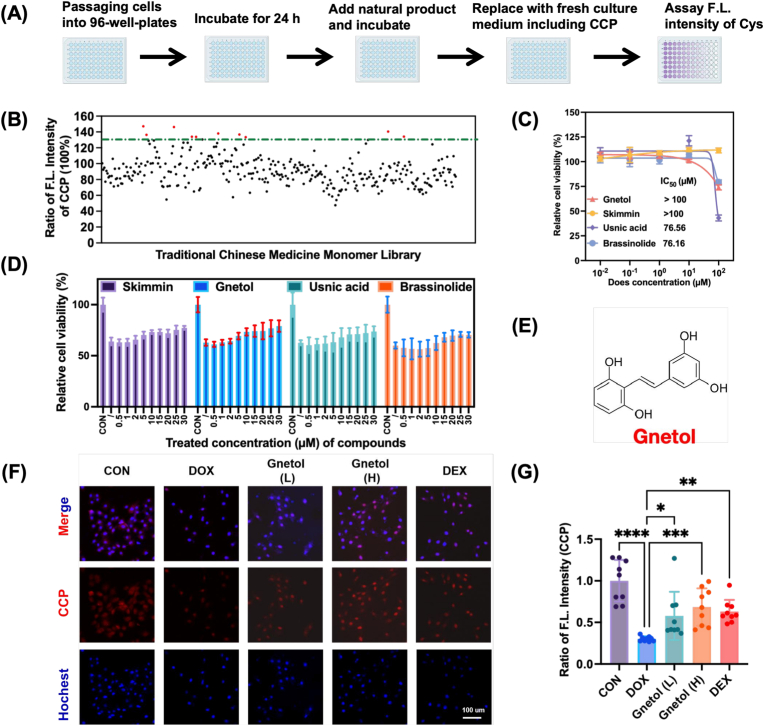
CCP-based screening identifies gnetol as an effective modulator of intracellular cysteine levels. (A) Schematic illustration of the CCP-guided screening strategy for identifying compounds that enhance intracellular cysteine under DOX stress. (B) Primary screening results of candidate compounds based on CCP fluorescence intensity (n = 3). (C) Cytotoxicity evaluation of the four candidate compounds in H9C2 cells. (D) Effects of candidate compounds on cell viability in DOX-treated H9C2 cells (n = 3). (E) Chemical structure of gnetol. (F) Confocal fluorescence images of CCP-stained H9C2 cells treated with gnetol under DOX challenge. (G) Quantitative analysis of intracellular CCP fluorescence intensity corresponding to (F) (n = 3). Scale bars: 100 μm. Data are presented as mean ± SD. ∗P < 0.05, ∗∗P < 0.01,∗∗∗P < 0.001,∗∗∗P < 0.0001.

Based on the excellent performance of CCP *in vitro* and *in vivo*, we established a CCP-based screening platform to identify small molecules capable of enhancing intracellular cysteine levels and mitigating DOX-induced cardiotoxicity (Fig. 3A). We screened a targeted natural compound library derived from traditional Chinese herbs using the fluorescence intensity of CCP as the primary readout. Initial screening identified four candidate compounds that significantly enhanced CCP fluorescence (Fig. 3B). These candidate compounds were then evaluated in subsequent biological assays. The cytotoxicity test showed that Gnetol and Skimmin had lower cytotoxicity, with IC_50_ values greater than 100 μM (Fig. 3C). Their cardioprotective potential was further evaluated in the DOX-induced H9C2 cell injury model. Gnetol significantly increased cell viability in a concentration-dependent manner (Fig. 3D). Based on cytotoxicity, protective efficacy, and the ability to restore intracellular Cys, Gnetol was selected for further study. Gnetol is a naturally occurring polyphenolic stilbene with reported antioxidant and cytoprotective properties [[33], [34], [35]](Fig. 3E). Confocal fluorescence imaging further confirmed that gnetol treatment (5 and 10 μM) markedly restored CCP fluorescence in DOX-treated H9C2 cells (Fig. 3F and G), indicating restoration of intracellular cysteine levels. These results identify gnetol as a potent intracellular cysteine modulator and support the feasibility of targeting cysteine homeostasis as an experimental approach to mitigate DOX-induced cardiotoxicity.

### 3.4 Gnetol attenuates DOX-induced ferroptotic injury through modulation of the cysteine–SMAD–hepcidin–FPN1 axis in H9C2 cardiomyocytes

**Fig. 4:**
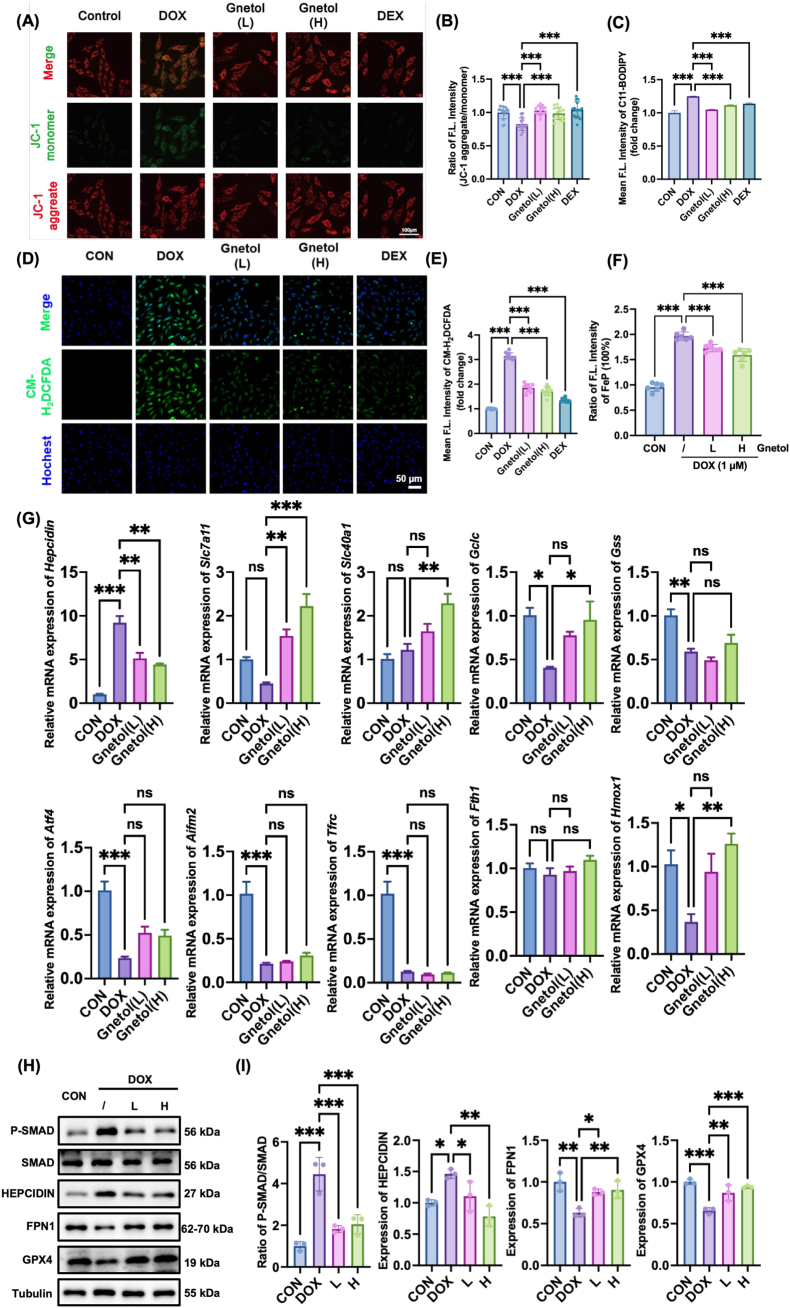
Gnetol suppresses DOX-induced ferroptotic injury in H9C2 cardiomyocytes. (A) Mitochondrial membrane potential assessed by JC-1 staining. (B) Quantification of JC-1 red/green fluorescence ratio. (C) Lipid peroxidation levels were quantified by flow cytometry following C11-BODIPY staining (n = 3). (D) Representative fluorescence images of cells stained with CM-H_2_DCFDA (green) to indicate cytosolic ROS and Hoechst 33342 (blue) to label nuclei. Scale bar, 50 μm. (E) Quantification of CM-H_2_DCFDA mean fluorescence intensity presented as fold change relative to CON (n = 3). (F) Intracellular Fe^2+^ levels detected using a Fe^2+^-sensitive fluorescent probe (n = 6). (G) Relative mRNA expression levels of ferroptosis- and cysteine-related genes (n = 3). (H) Western blot analysis of phosphorylated SMAD1/5/9, total SMAD1/5/9, ferroportin (FPN1), and GPX4. (I) Quantitative analysis of protein expression shown in H (n = 3). Data are presented as mean ± SD (n ≥ 3). ∗P < 0.05, ∗∗P < 0.01,∗∗∗P < 0.001.

Because cysteine metabolism is closely linked to ferroptosis, we examined whether gnetol could attenuate DOX-induced ferroptotic injury in H9C2 cardiomyocytes. Mitochondrial membrane potential (MMP), which reflects early mitochondrial dysfunction during ferroptosis, was examined using JC-1 staining. DOX treatment caused a marked loss of MMP, whereas gnetol partially restored MMP to a level comparable with that observed following dexrazoxane (DEX) treatment (Fig. 4A and B). In parallel with improved mitochondrial function, gnetol suppressed DOX-induced oxidative stress. Lipid peroxidation, a hallmark of ferroptosis, was further assessed using C11-BODIPY staining. DOX significantly increased the level of lipid peroxides, while gnetol effectively alleviated it (Fig. 4C). The flow cytometry analysis using the fluorescent probe CM-H_2_DCFDA and DCFH-DA for reactive oxygen species (ROS) showed that DOX exposure led to marked ROS accumulation, whereas gnetol treatment reduced ROS levels (Fig. 4D, E, S10). Additionally, after DOX treatment, the intracellular Fe^2+^ level increased, but it was significantly reduced after gnetol treatment, indicating attenuation of intracellular iron accumulation (Fig. 4F). To explore the molecular basis of these ferroptosis-related changes, we measured the expression levels of genes involved in the cysteine-ferroptosis metabolic network. Among the genes examined, hepcidin showed the strongest upregulation in response to DOX, and this upregulation was significantly inhibited by gnetol (Fig. 4G). Because of its established role in ferroptosis regulation, hepcidin was selected for further mechanistic analysis [36]. Western blot analysis revealed that the SMAD1/5/9 signaling pathway was activated after DOX treatment, while gnetol significantly inhibited the phosphorylation of SMAD (Fig. 4H and I). Since hepcidin promotes the internalization and degradation of ferroportin (FPN1), the major cellular iron exporter, FPN1 expression was subsequently examined [37,38]. Gnetol significantly restored FPN1 protein levels that were otherwise suppressed by DOX. In addition, DOX-induced downregulation of glutathione peroxidase 4 (GPX4), a key ferroptosis suppressor, was markedly reversed by gnetol. Together, these results indicate that gnetol attenuates DOX-induced ferroptotic injury in cardiomyocytes by restoring intracellular cysteine homeostasis and modulating the SMAD-hepcidin-FPN1 axis alongside GPX4-mediated lipid peroxide detoxification.

### 3.5 Gnetol attenuates DOX-induced cardiomyopathy *in vivo*

**Fig. 5:**
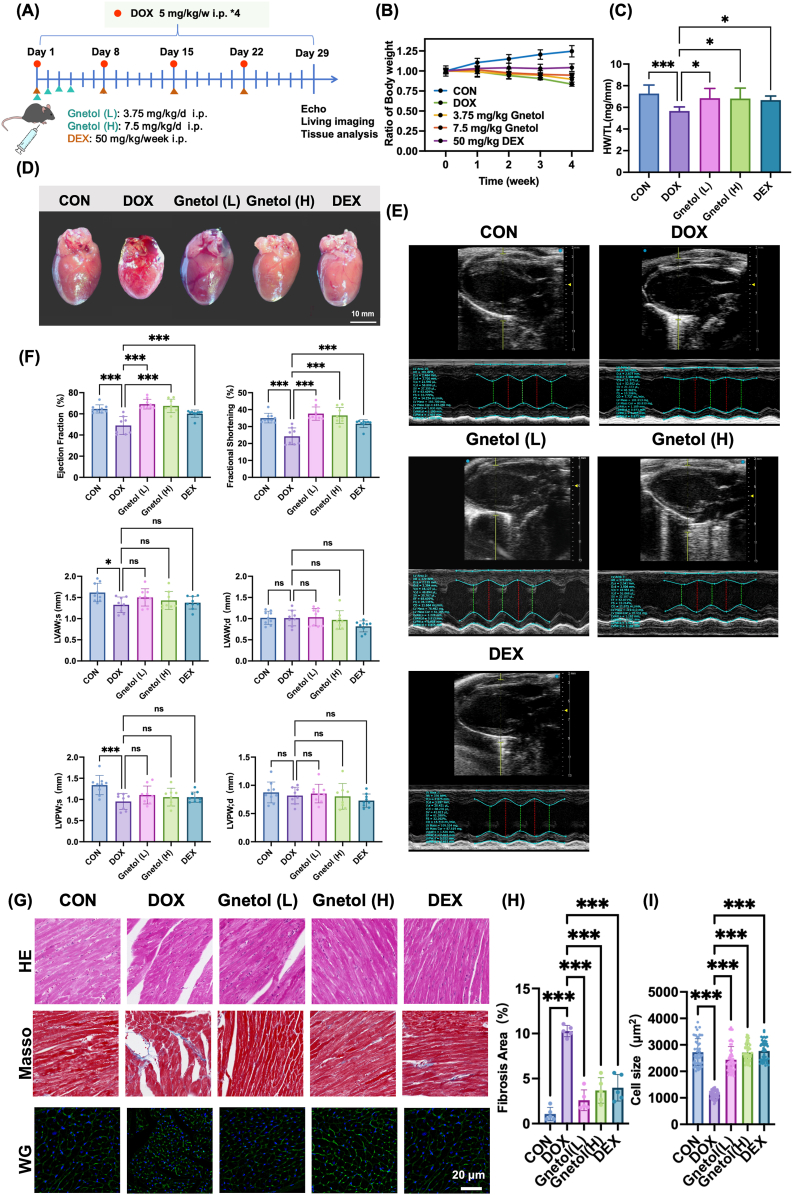
Gnetol ameliorates doxorubicin-induced cardiomyopathy *in vivo*. (A) Experimental scheme of the DOX-induced cardiomyopathy model and gnetol treatment. (B) Changes in body weight during the treatment period. (C) Heart weight-to-tibia length ratio (HW/TL). (D) Representative gross morphology of hearts from each group. (E) Representative M-mode echocardiographic images. (F) Quantitative analysis of cardiac function parameters. (G) Representative histological images of heart sections stained with H&E, Masson's trichrome, and wheat germ agglutinin (WGA). (H) Quantification of myocardial fibrosis area. (I) Quantification of cardiomyocyte cross-sectional area. Data are presented as mean ± SD (n = 6-8). ∗P < 0.05, ∗∗P < 0.01,∗∗∗P < 0.001.

To evaluate the cardioprotective effects of gnetol *in vivo*, a mouse model of DOX-induced cardiomyopathy was established (Fig. 5A). Repeated DOX administration resulted in significant body weight loss, whereas gnetol treatment partially alleviated DOX-induced weight reduction, suggesting partial improvement in overall status (Fig. 5B). In parallel, DOX significantly decreased the heart weight–to–tibia length ratio (HW/TL), which was restored by gnetol administration (Fig. 5C). As expected, DOX caused cardiac atrophy, as evidenced by the significant reduction in the overall size of the heart, which was partially reversed by gnetol treatment (Fig. 5D). Echocardiographic assessment revealed that DOX significantly impaired cardiac contractile function, as reflected by reductions in ejection fraction (EF) and fractional shortening (FS). Compared with the DOX group, low-dose (3.75 mg/kg) and high-dose (7.5 mg/kg) gnetol treatment increased EF and FS (Fig. 5E and F). These functional improvements were supported by histological analyses. Hematoxylin-eosin (H&E) staining showed that the hearts treated with DOX exhibited severe myocardial disorders, whereas gnetol treatment partially improved myocardial architecture (Fig. 5G). Masson staining showed extensive interstitial fibrosis following DOX exposure, and gnetol significantly reduced collagen deposition (Fig. 5G and H). Wheat germ agglutinin (WGA) staining showed that DOX-induced myocardial cell atrophy was reversed under gnetol treatment (Fig. 5G and I). Additionally, histological examinations of major organs including the liver, spleen, lung, and kidney revealed no obvious pathological changes after gnetol treatment (Fig. S11), indicating no overt toxicity *in vivo*. Overall, these results suggest that gnetol can effectively alleviate cardiomyopathy caused by DOX, and preserve cardiac structure and contractile function *in vivo*.

### 3.6 Gnetol attenuates DOX-induced myocardial ferroptotic injury *in vivo*

**Fig. 6:**
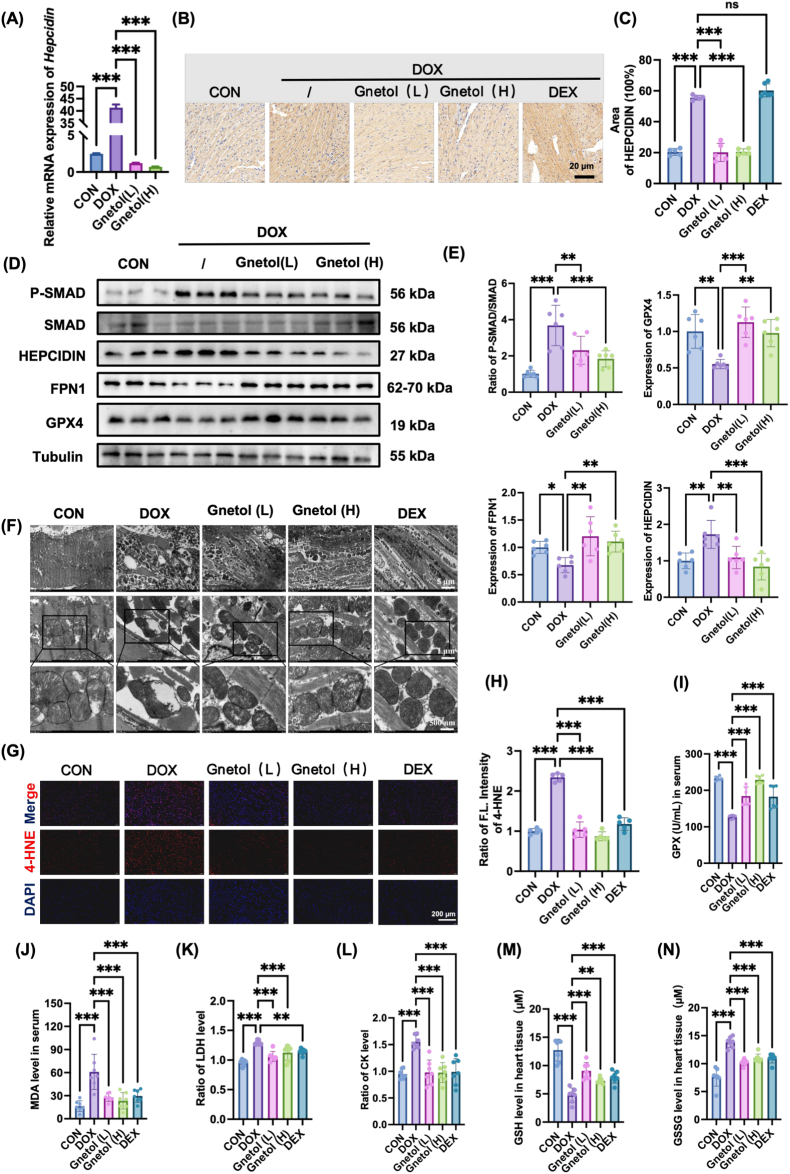
Gnetol attenuates myocardial ferroptotic injury *in vivo* by modulating the SMAD–hepcidin–FPN1 axis. (A) Relative mRNA expression levels of hepcidin in cardiac tissues. (B) Representative immunohistochemical staining of hepcidin in heart sections. (C) Quantitative analysis of hepcidin-positive staining area. (D) Western blot analysis of phosphorylated SMAD1/5/9, total SMAD1/5/9, hepcidin, FPN1, and GPX4 in cardiac tissues. (E) Densitometric quantification of protein expression shown in (D). (F) Transmission electron microscopy images showing mitochondrial ultrastructure. (G) Representative immunofluorescence images of 4-hydroxynonenal (4-HNE) staining. (H) Quantitative analysis of 4-HNE fluorescence intensity. (I–L) Serum biochemical indicators of oxidative injury and myocardial damage. (M, N) Levels of GSH and GSSG in cardiac tissues. Data are presented as mean ± SD (n = 3). ∗P < 0.05, ∗∗P < 0.01,∗∗∗P < 0.001.

Based on the *in vitro* findings, hepcidin expression was further examined in cardiac tissues. DOX treatment markedly upregulated myocardial hepcidin mRNA levels, which were reduced by gnetol administration (Fig. 6A). Immunohistochemical analysis further confirmed increased hepcidin accumulation in the myocardium after DOX treatment, while gnetol significantly reduced the expression of hepcidin (Fig. 6B and C). Moreover, Western blot analysis showed that SMAD1/5/9 phosphorylation was increased following DOX treatment, while gnetol significantly inhibited the activation of SMAD without altering total SMAD protein levels (Fig. 6D and E). In parallel, gnetol reduced the protein expression of hepcidin and restored the level of FPN1, suggesting attenuation of iron accumulation in cardiac tissue. At the same time, gnetol significantly restored the expression of GPX4 in the myocardium and thereby increasing resistance to ferroptotic injury. Immunofluorescence analysis further supported restoration of GPX4 and FPN1 expression in the hearts treated with gnetol (Figure S12 and Figure S13). Transmission electron microscopy (TEM) revealed mitochondrial ultrastructural features characteristic of ferroptosis. Mitochondria in DOX-treated myocardial cells were swollen, cristae structure was disrupted,and the membrane integrity was damaged. These ultrastructural abnormalities were partially alleviated following gnetol treatment (Fig. 6F). Lipid peroxidation was further evaluated by 4-hydroxynonenal (4-HNE) immunofluorescence staining [39]. DOX induced increased 4-HNE accumulation in cardiac tissues, whereas gnetol significantly reduced lipid peroxidation signals (Fig. 6G and H). In line with these findings, biochemical analyses showed that gnetol normalized markers of oxidative damage and myocardial injury (including glutathione peroxidase (GPX), malondialdehyde (MDA), lactate dehydrogenase (LDH), and creatine kinase (CK)) in the serum (Fig. 6I–L). Additionally, gnetol improved myocardial redox balance by increasing the level of reduced glutathione (GSH) and reducing the level of oxidized glutathione (GSSG) (Fig. 6M and N). In summary, these findings suggest that modulation of the SMAD–hepcidin–FPN1 axis contributes to gnetol-mediated restoration of cysteine-dependent redox balance and attenuation of myocardial ferroptosis *in vivo*.

## 4 Discussion

In this study, we established a probe-enabled strategy that links early redox alterations with mechanistic insight and experimental intervention in anthracycline-induced cardiotoxicity. Several key findings emerge. First, we developed a cysteine-activatable fluorescent probe, CCP, which enables sensitive and selective imaging of intracellular cysteine dynamics in biological systems. Second, using CCP-based imaging, we identified intracellular cysteine depletion as an early molecular alteration during DOX-induced cardiac injury. Notably, the decline in cardiac CCP signal was detectable at three weeks *in vivo*, preceding the onset of echocardiographically measurable systolic dysfunction at four weeks. Third, by leveraging CCP as a functional readout for screening, we identified gnetol as an effective modulator of intracellular cysteine homeostasis that attenuates DOX-induced cardiomyocyte injury and improves cardiac structure and function in mice. Finally, our data suggest that gnetol mitigates ferroptotic myocardial injury via modulation of the cysteine-dependent antioxidant defense system and iron metabolism regulation.

Current clinical surveillance of anthracycline cardiotoxicity relies largely on functional imaging parameters, such as ejection fraction, which typically reflect relatively late stages of myocardial injury [5]. Our findings show cysteine depletion as an early molecular event before overt dysfunction. Importantly, cysteine loss is not merely a nonspecific stress response but lies at the intersection of redox buffering and ferroptosis regulation [9,40]. As the limiting substrate for glutathione synthesis, cysteine directly governs GPX4-mediated detoxification of lipid peroxides, thereby influencing cellular susceptibility to ferroptotic injury. These findings highlight intracellular cysteine as a biologically interpretable early redox feature of DOX-induced cardiotoxicity.

Ferroptosis has emerged as a critical contributor to DOX-induced cardiomyocyte damage. *In vitro*, gnetol restored mitochondrial membrane potential, reduced reactive oxygen species accumulation, limited lipid peroxidation, and attenuated intracellular iron accumulation, all of which are characteristic features of ferroptosis suppression [41]. Mechanistic analyses further implicate the SMAD–hepcidin–ferroportin axis as a key regulatory pathway underlying these effects. Hepcidin is a central regulator of systemic and cellular iron homeostasis and promotes ferroportin internalization and degradation, thereby restricting cellular iron export [37,38]. In our study, DOX activated SMAD1/5/9 signaling and upregulated hepcidin expression, leading to reduced ferroportin levels and iron retention, whereas gnetol counteracted these changes in both cardiomyocytes and cardiac tissue. In parallel, gnetol restored GPX4 expression and glutathione-dependent antioxidant capacity, linking iron handling with cysteine-dependent redox defense. Together, these findings position cysteine as an upstream metabolic node integrating iron metabolism and antioxidant protection in the regulation of ferroptotic injury.

The protective effects of gnetol were further supported by *in vivo* evidence. In a DOX-induced cardiomyopathy model, gnetol improved cardiac contractile performance, reduced myocardial fibrosis, and attenuated cardiomyocyte atrophy. At the ultrastructural level, mitochondrial integrity was better preserved, while lipid peroxidation and oxidative damage markers were reduced [40,42]. Consistent improvements in myocardial redox balance and serum indicators of cardiac injury further support suppression of ferroptosis *in vivo*. Importantly, histological analysis of major organs revealed no overt pathological alterations under the tested conditions, suggesting an acceptable safety profile in this experimental setting.

Several limitations should be acknowledged. Although gnetol exhibited cardioprotective effects in preclinical models, further studies are required to define its pharmacokinetic properties, optimal dosing, and long-term safety. In addition, the potential impact of gnetol on the antitumor efficacy of doxorubicin warrants careful evaluation. With respect to broader applicability, our experiments were performed primarily in male C57BL/6 mice using a single DOX administration regimen; extension to other strains, female animals, and clinically relevant chronic dosing schedules will be important in future work. Mechanistically, although our data support that gnetol restores intracellular cysteine homeostasis and suppresses ferroptotic injury, additional genetic validation targeting cysteine transporters or key metabolic enzymes would further strengthen causal inference and help to more clearly distinguish cysteine depletion from downstream GSH depletion. Finally, future studies exploring potential direct interactions between gnetol and candidate protein targets within the ferroptosis pathway may refine the molecular framework proposed here. [43]. Addressing these issues will be important for future studies aimed at extending cysteine-targeted redox modulation beyond the current experimental setting.

In summary, this work identifies intracellular cysteine depletion as an early redox alteration in DOX-induced cardiotoxicity and demonstrates that cysteine-targeted imaging can facilitate mechanism-guided discovery of ferroptosis-modulating agents. By linking early redox changes to iron metabolism and antioxidant defense, our findings provide a mechanistic framework for understanding and experimentally modulating ferroptotic cardiac injury.

## 5 Conclusion

In summary, this study identifies intracellular cysteine depletion as an early redox alteration during DOX-induced cardiotoxicity and demonstrates the potential of cysteine-activatable imaging to detect early molecular changes and facilitate mechanism-guided experimental discovery. We show that gnetol attenuates DOX-induced cardiomyopathy by inhibiting ferroptosis, through modulation of the SMAD-hepcidin-FPN1 axis and reinforcement of the glutathione-GPX4 antioxidant defense system. Together, these findings establish a mechanistic link between cysteine homeostasis, iron metabolism, and ferroptotic injury in the heart, and provide an experimental framework for studying and modulating redox-driven cardiac damage associated with anthracycline treatment.

## Ethics approval and consent to participate

All experimental procedures were approved by the Experimental Animal Ethics Committee of Wuxi Medical College of Jiangnan University (Permission Code: JN.No20241115c0450530[596]) and conducted in accordance with the National Institute of Laboratory Animal Health guidelines.

## Consent for publication

Not applicable.

## Funding

The authors would like to acknowledge the 10.13039/501100005145Basic Research Program of Jiangsu Province (BK20231034), the 10.13039/501100001809National Natural Science Foundation of China (Grant No. 22407050, 22307006, 82200403), the 10.13039/501100002949Jiangsu Province Young Scientific and Technological Talents Promotion Programme‌ (JSTJ-2025-486) and the 10.13039/501100018537National Science and Technology Major Project of the Ministry of Science and Technology of China (2023ZD0504601) and the Leading Innovative Talent Introduction and Cultivation Project of Changzhou City (CQ20240112).

## CRediT authorship contribution statement

**Yan Chen:** Investigation, Methodology, Visualization, Writing – original draft. **Bo Zhang:** Conceptualization, Funding acquisition, Writing – review & editing. **Yufan Wei:** Investigation, Methodology, Software. **Yanfa Dai:** Data curation, Investigation. **Baoyue Zhang:** Data curation, Investigation. **Jing Li:** Data curation, Investigation. **Ke-Jia Wu:** Funding acquisition, Supervision. **Ning Sun:** Funding acquisition, Investigation, Supervision, Writing – review & editing. **Chenwen Shao:** Conceptualization, Funding acquisition, Supervision, Writing – review & editing.

## Declaration of competing interest

The authors declare that they have no known competing financial interests or personal relationships that could have appeared to influence the work reported in this paper.

## Data Availability

Data will be made available on request.
