# Supplementary material for: Cysteine imaging reveals early redox dysregulation and identifies gnetol as a ferroptosis-modulating agent in doxorubicin cardiotoxicity
Source: Redox Biol. 2026 Mar 21;92:104131. doi: 10.1016/j.redox.2026.104131 (PMC13049667; doi:10.1016/j.redox.2026.104131)

psmad-cell

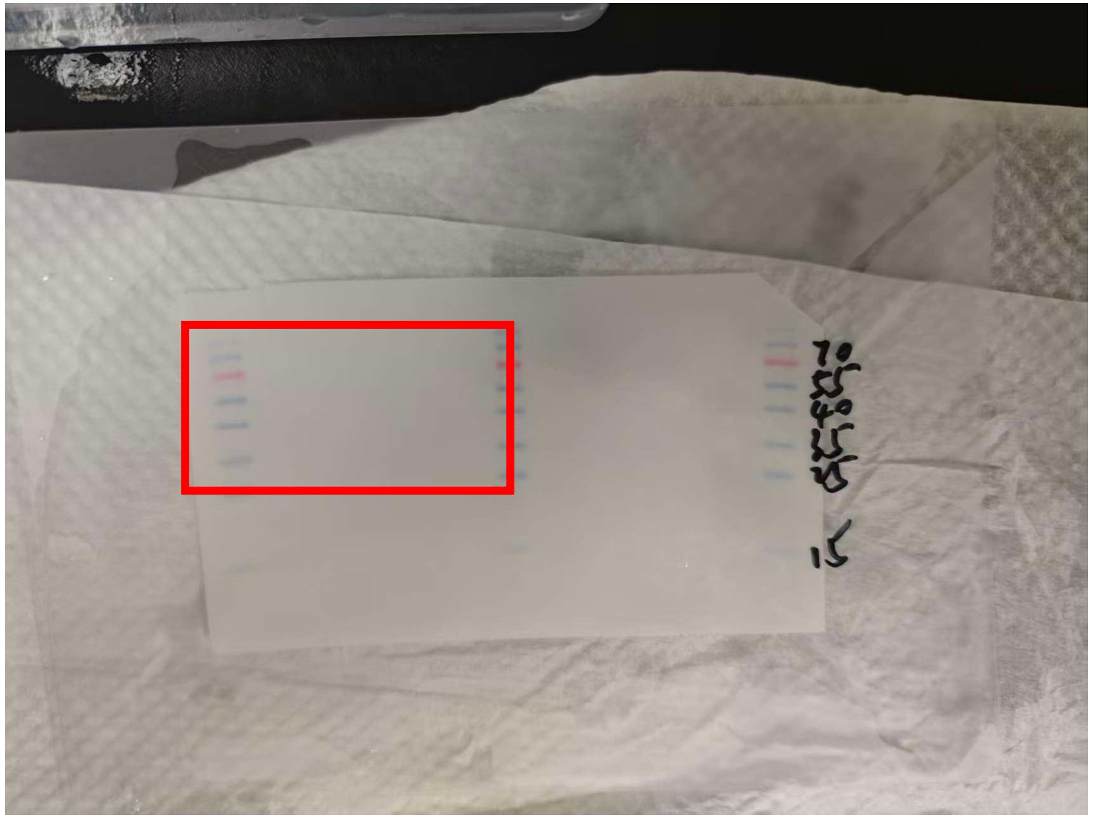

psmad-g
56kda

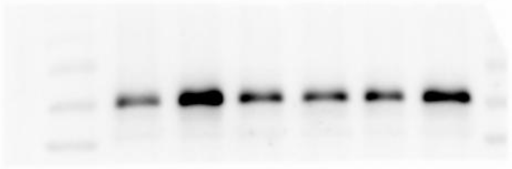

psmad-m

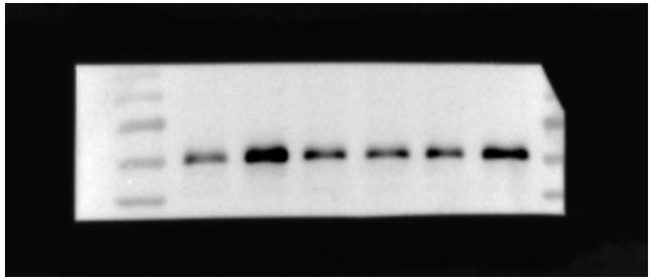

tubulin-g
55kda

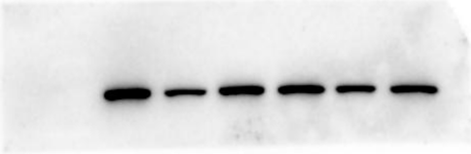

tubulin-m

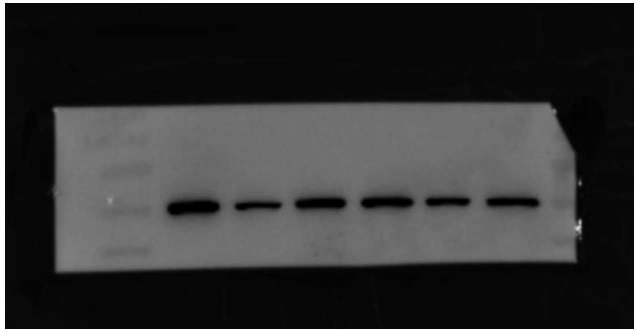

smad-cell
8.22

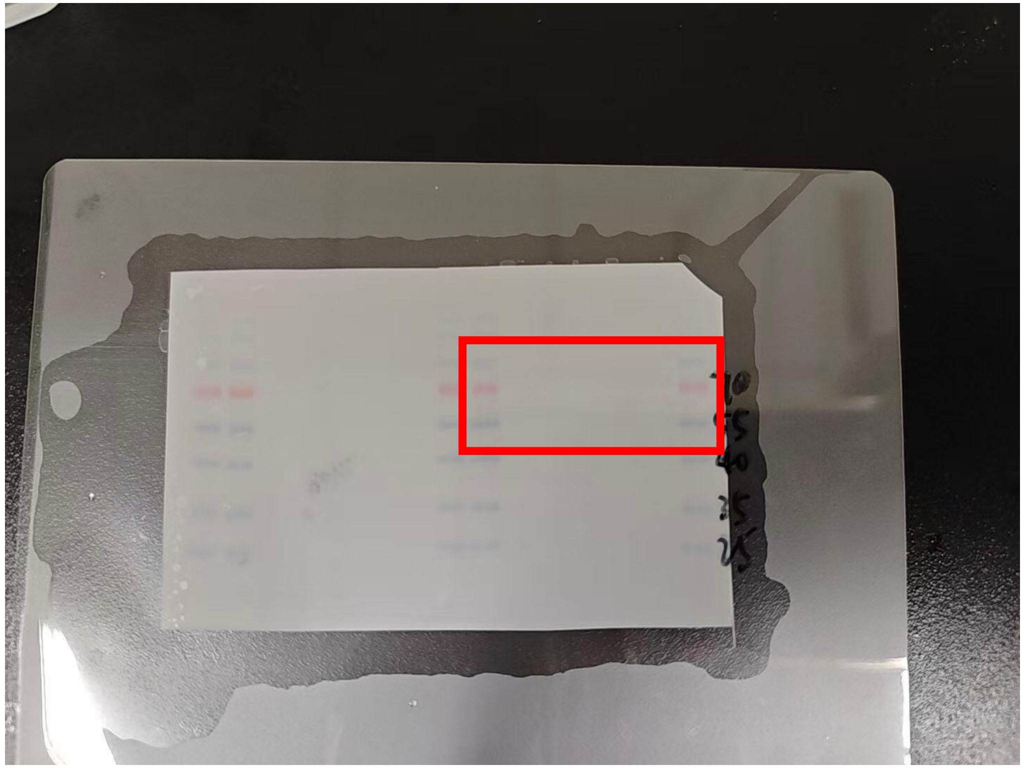

smad-g
56kda

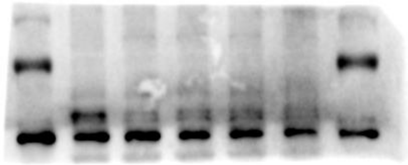

smad-m

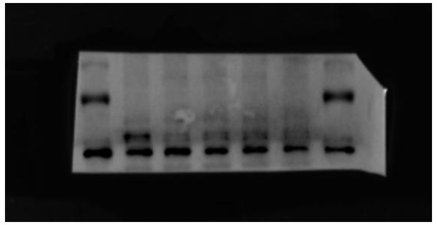

tubulin-g
55kda

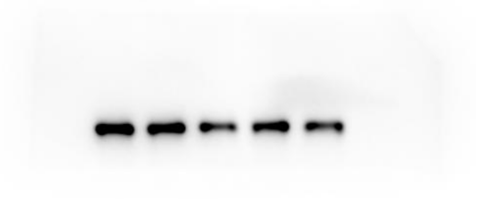

tubulin-m

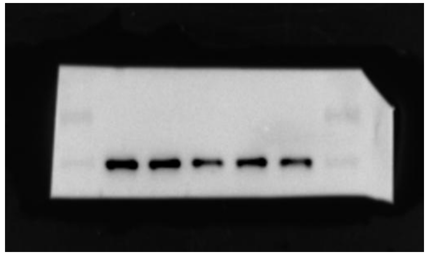

hepcidin-cell

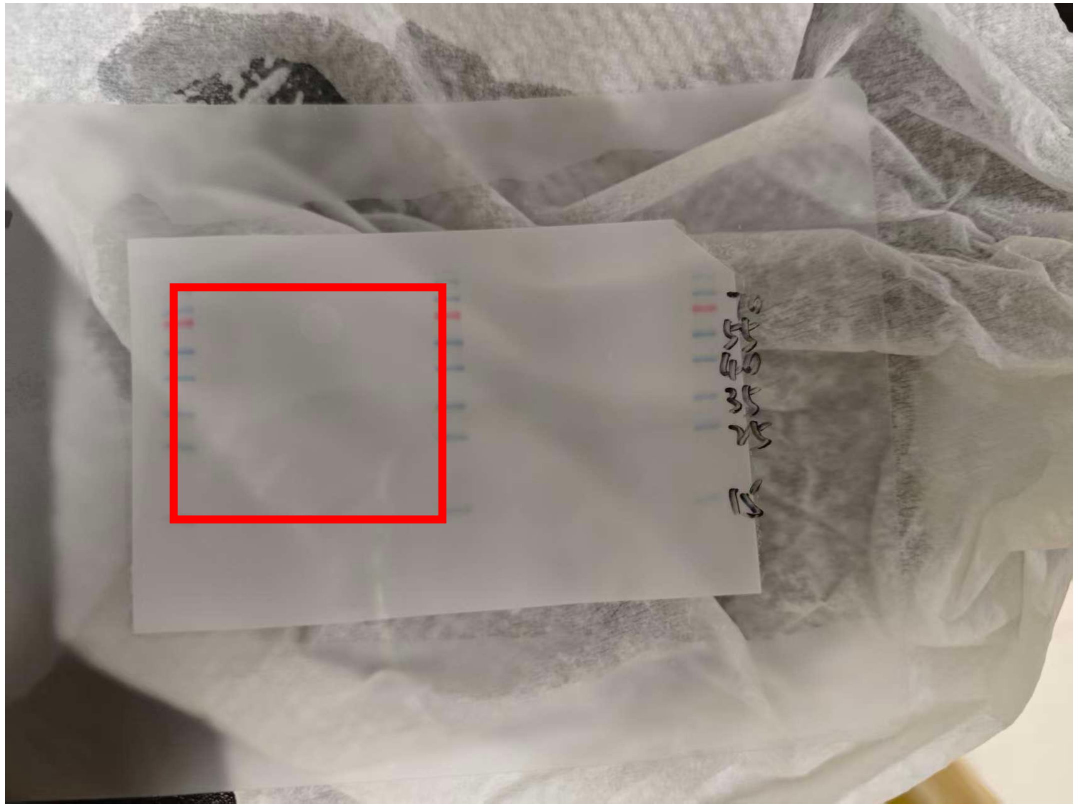

hepcidin-g
27kda

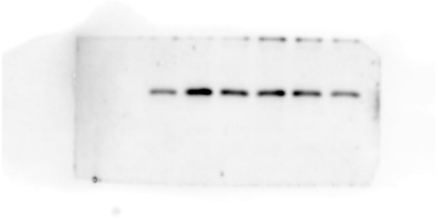

hepcidin-m

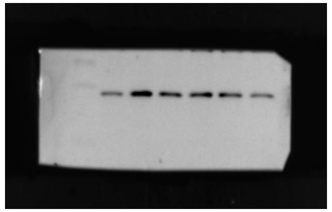

tubulin-g
55kda

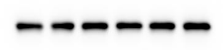

tubulin-m

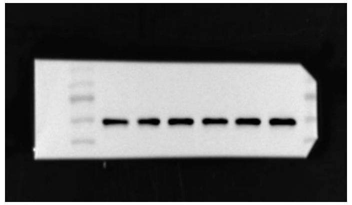

fpn-cell 9.4

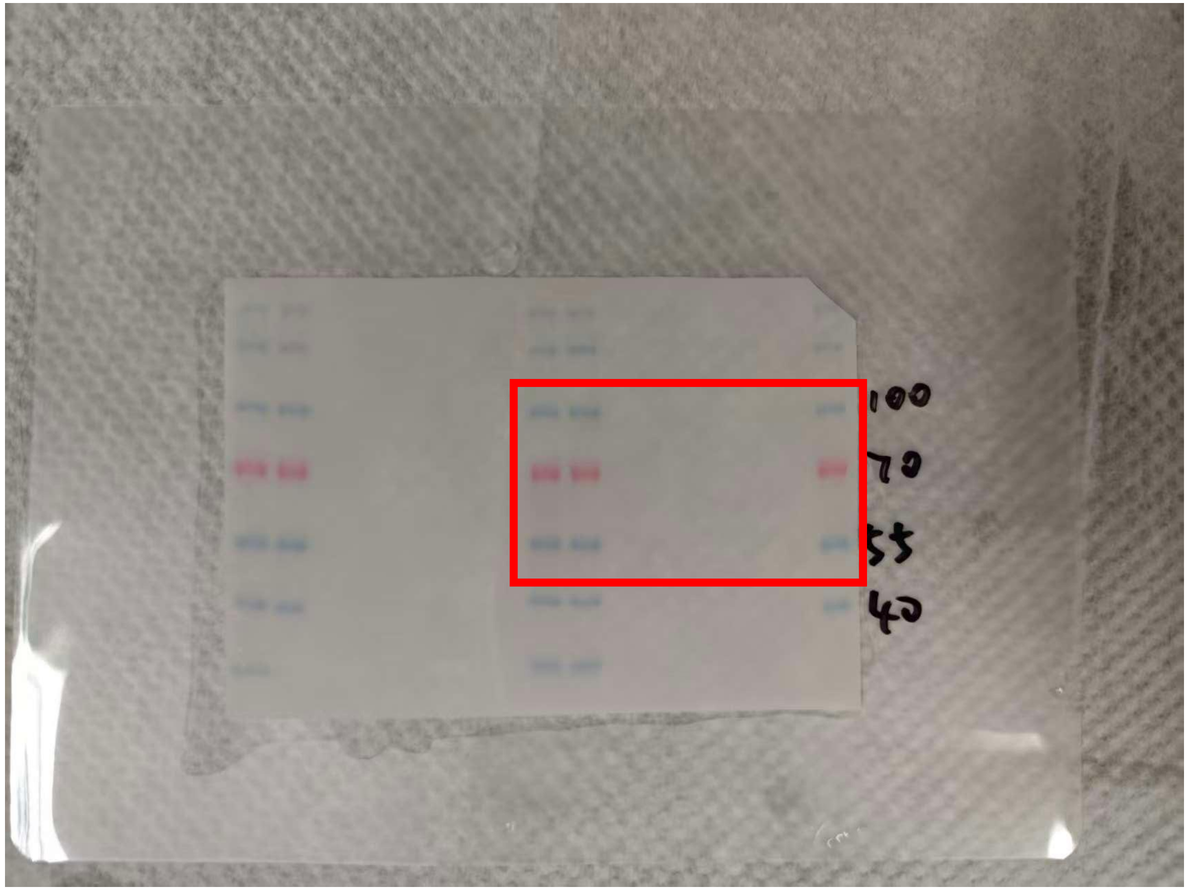

fpn-g
62-
70kda

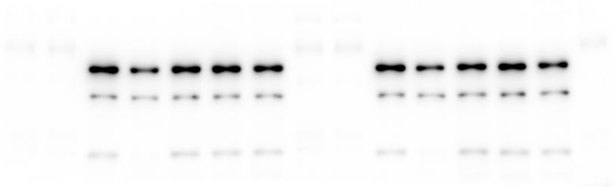

fpn-m
62-
70kda

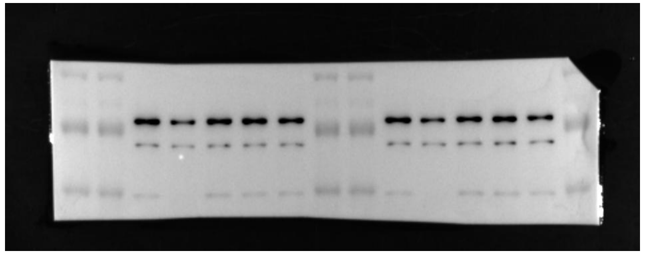

tubulin-g
55kda

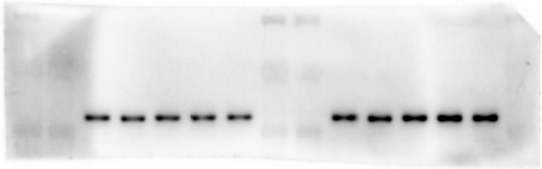

tubulin-
m

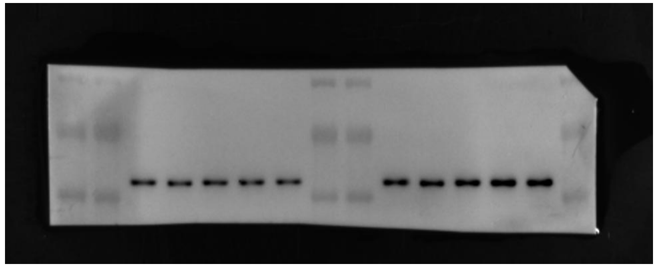

gpx4-cell
7.20

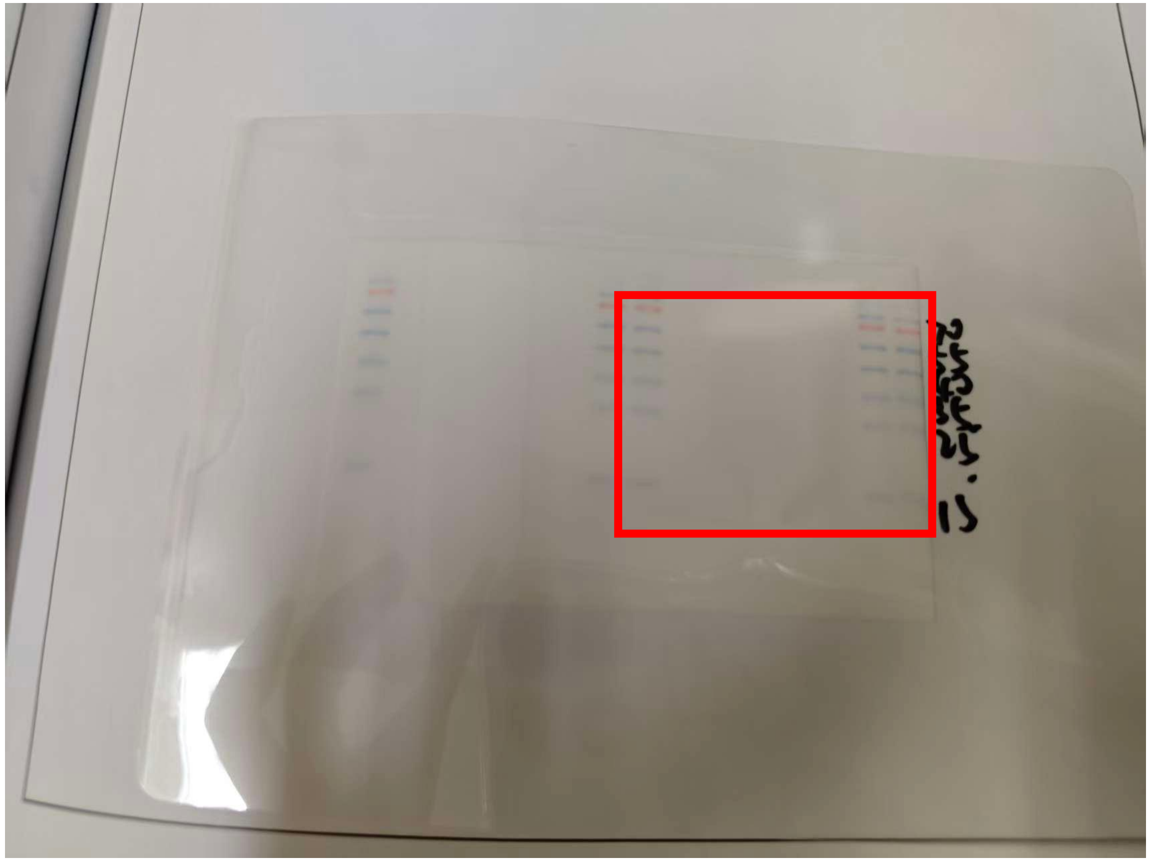

gpx4-g
19kda

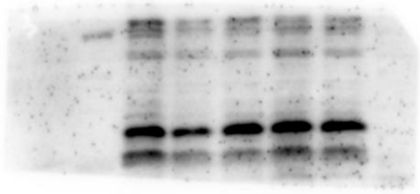

gpx4-m

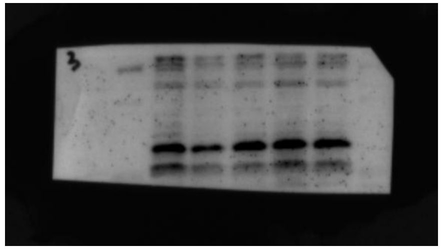

tubulin-g
55kda

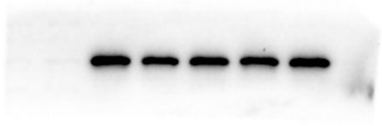

tubulin-
m

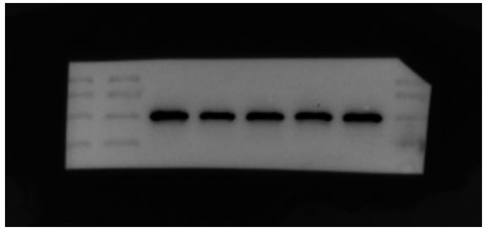

psmad-
animal

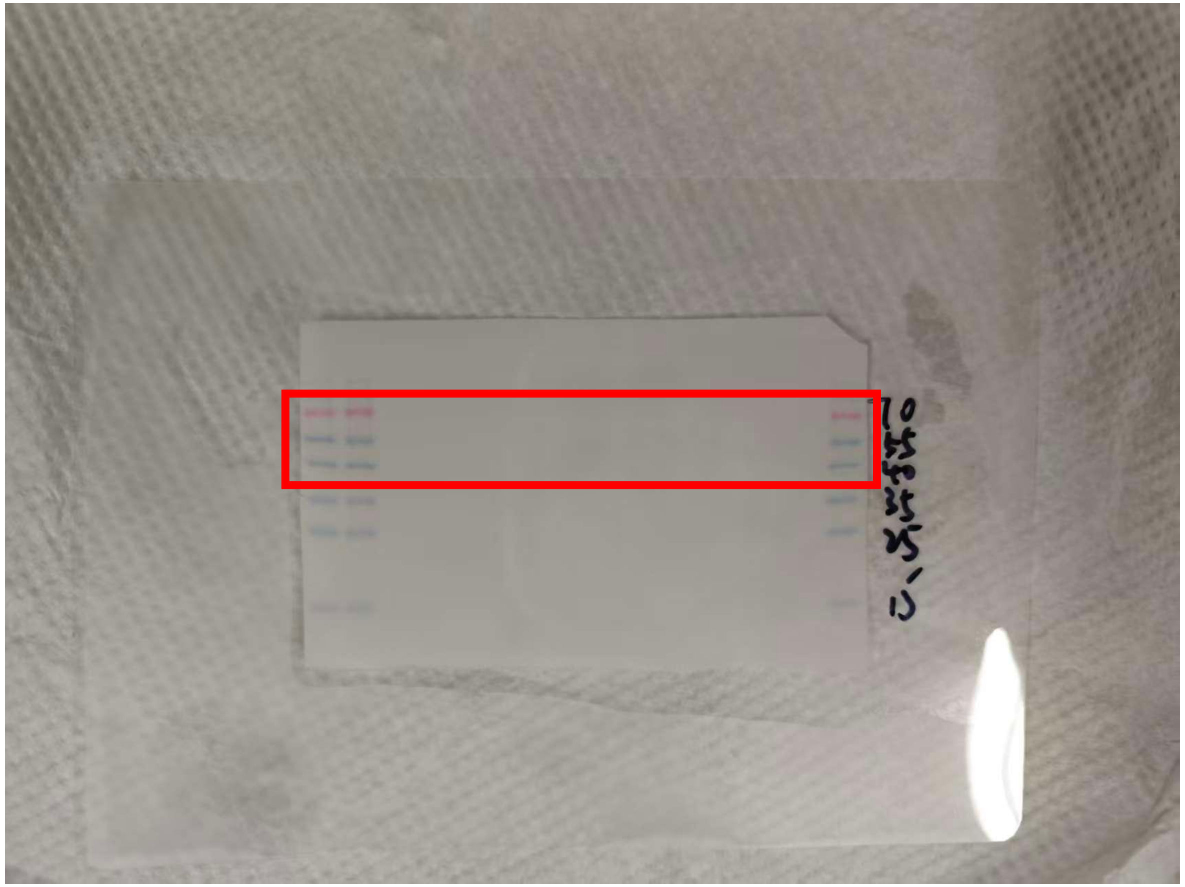

psmad-g
56kda

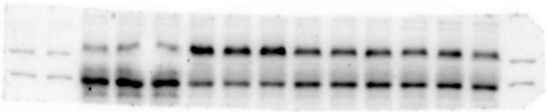

psmad-m

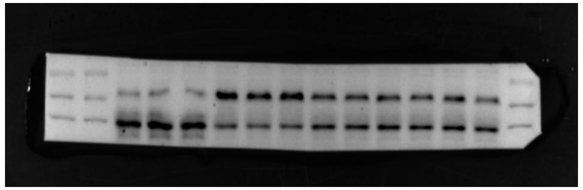

tubulin-g
55kda

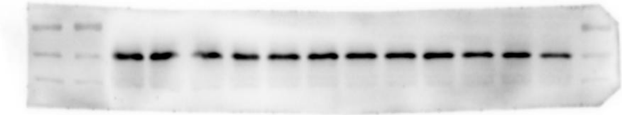

tubulin-m

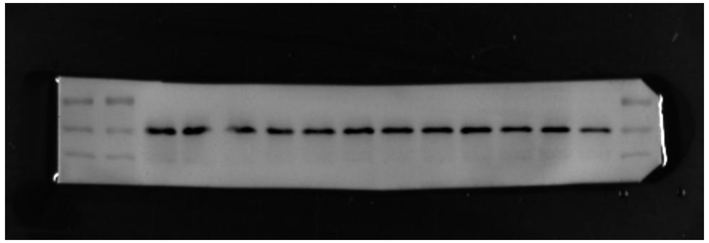

smad-animal
9.16

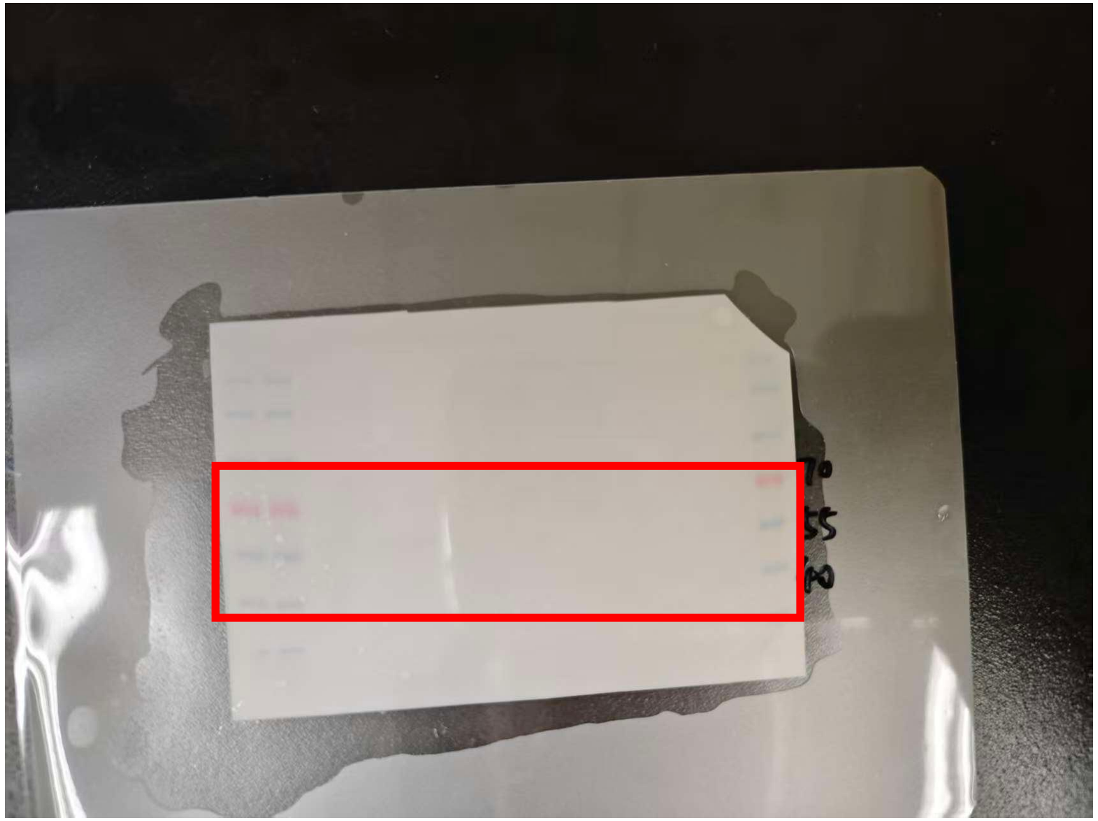

smad-g
56kda

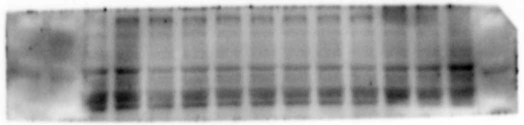

smad-m

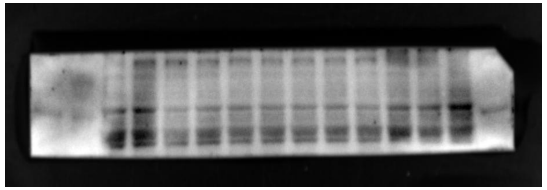

tubulin-g
55kda

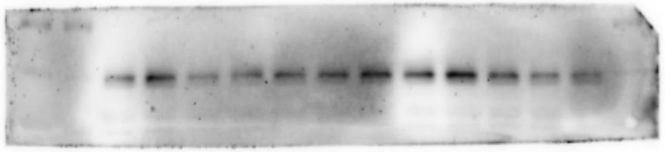

tubulin-m

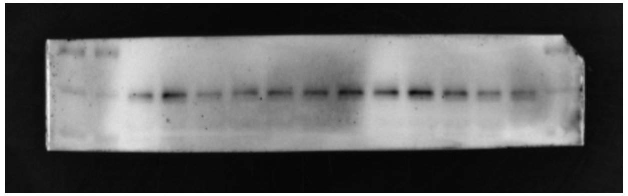

hepcidin-animal
7.28

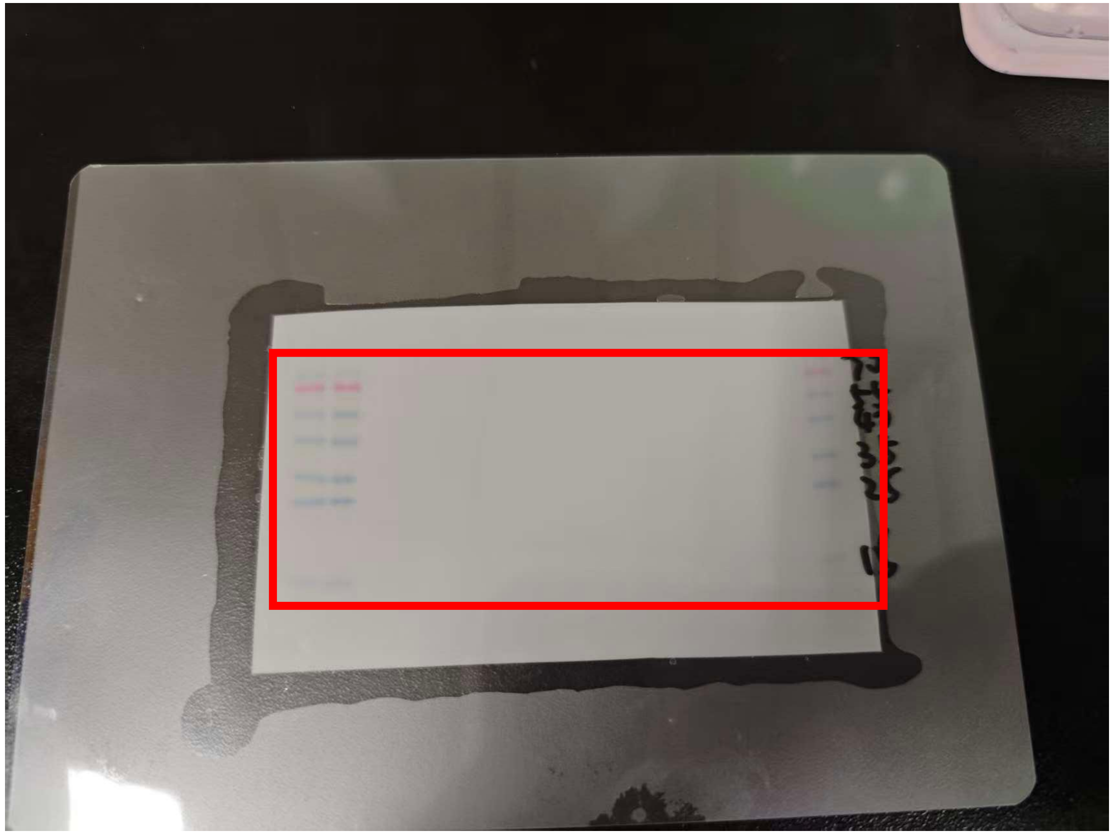

hepcidin-g
27kda

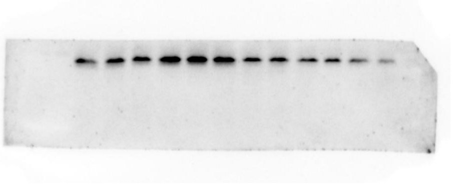

hepcidin-m

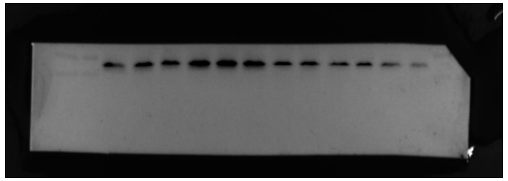

tubulin-g
55kda

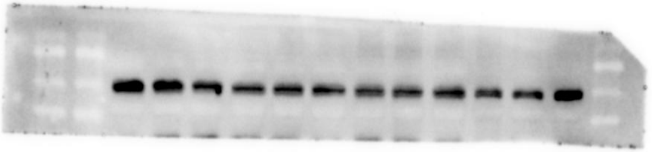

tubulin-m

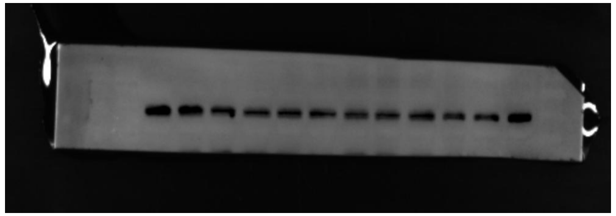

gpx4-animal
9.10

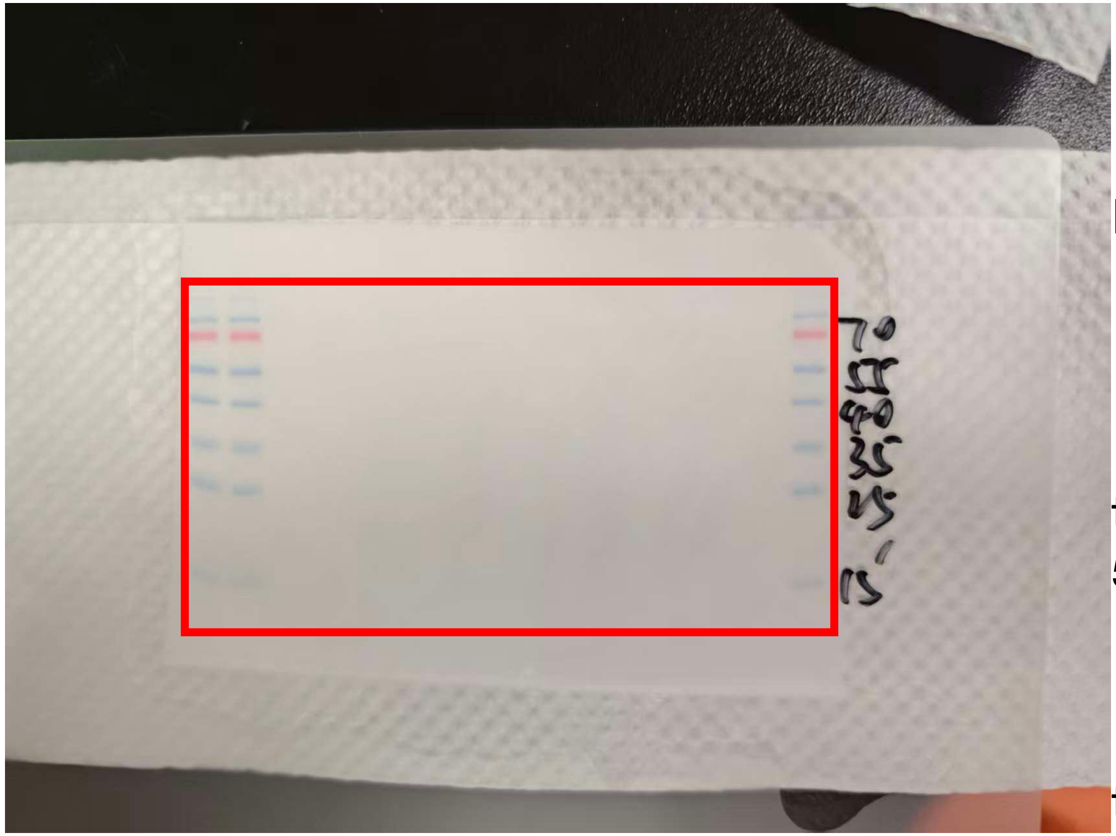

gpx4-g
19kda

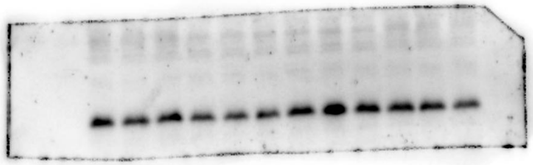

px4-m

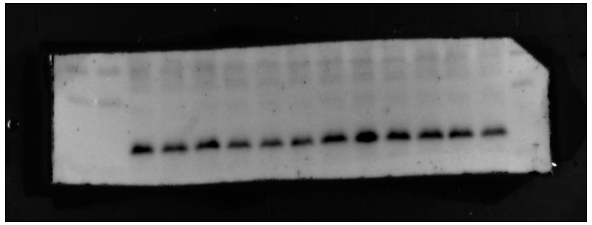

tubulin-g
55kda

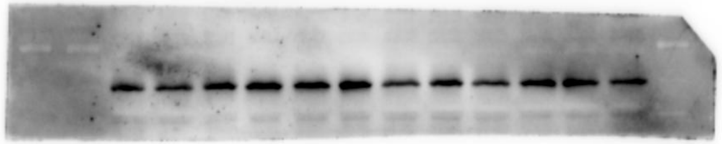

tubulin-m

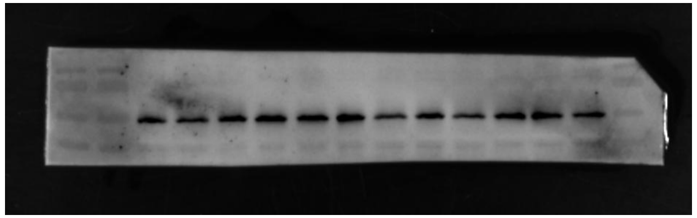

fpn-animal
8.15

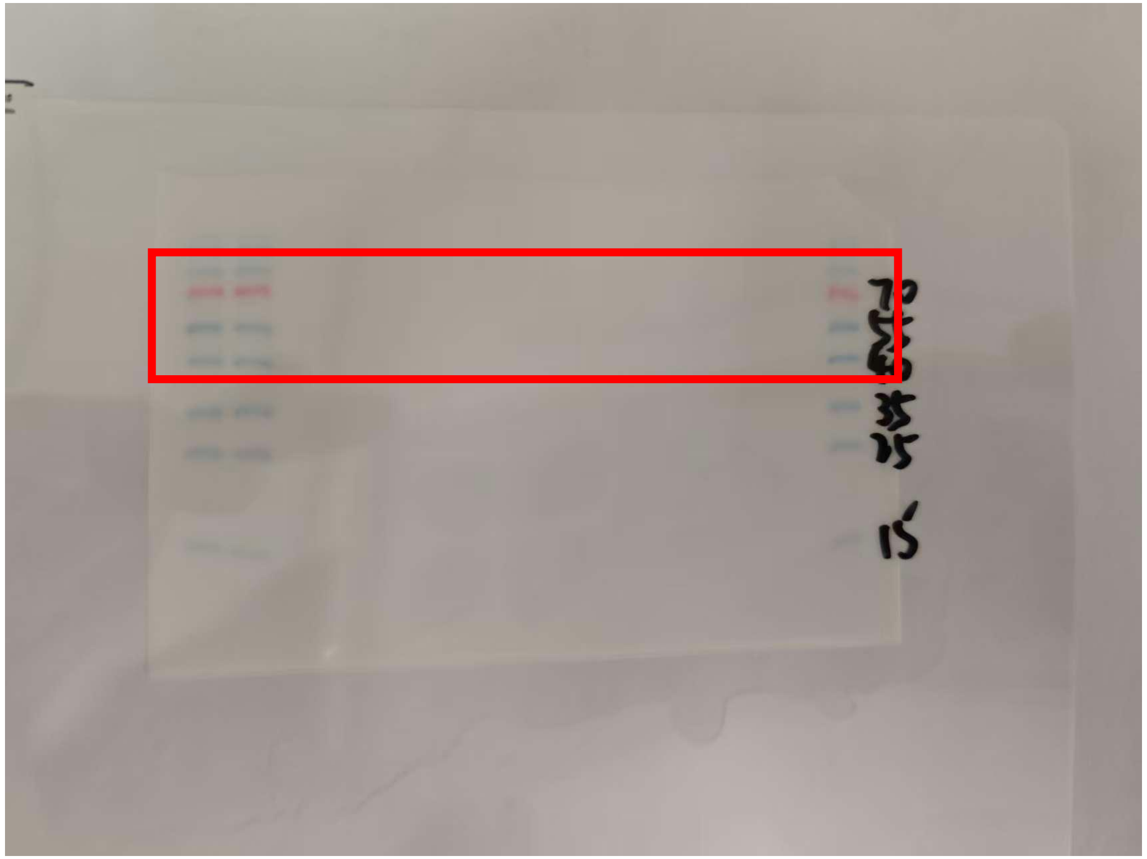

fpn-g
62-
70kda

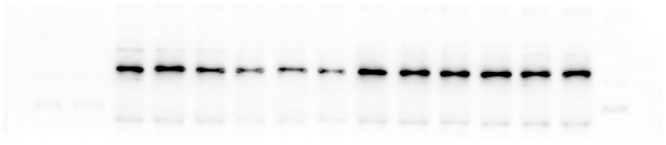

fpn-m
62-
70kda

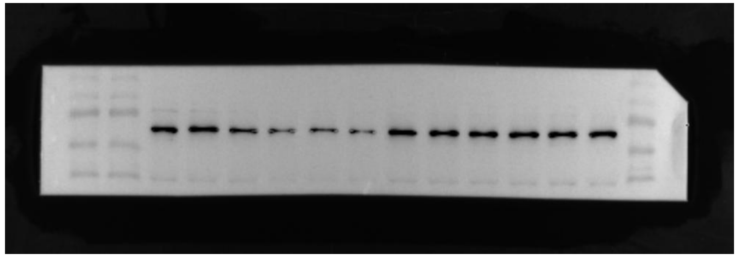

tubulin-g
55kda

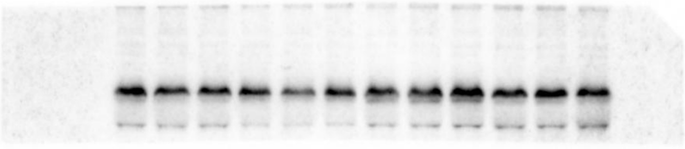

tubulin-
m

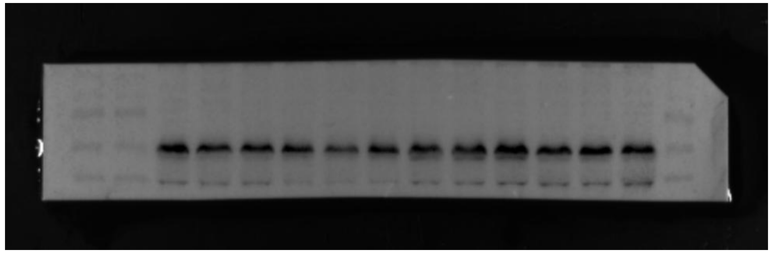

Supplement: Multimedia component 1 [file mmc1.pdf]
