# Supplementary material for: Cysteine imaging reveals early redox dysregulation and identifies gnetol as a ferroptosis-modulating agent in doxorubicin cardiotoxicity
Source: Redox Biol. 2026 Mar 21;92:104131. doi: 10.1016/j.redox.2026.104131 (PMC13049667; doi:10.1016/j.redox.2026.104131)
Supplement: Multimedia component 2 [file mmc2.docx]

**Cysteine imaging reveals early redox dysregulation and identifies gnetol as a ferroptosis-modulating agent in doxorubicin cardiotoxicity**

Yan Chen^1,3,#^, Bo Zhang^2,#,^*, Yufan Wei^1,#^, Yanfa Dai^1^, Baoyue Zhang^1^, Jing Li^1^, Ke-Jia Wu^1,3^*, Ning Sun^1,3^*, Chenwen Shao^1,3^*

1 Wuxi School of Medicine, Jiangnan University, Wuxi, Jiangsu, 214122, China

2 School of Pharmacy, Changzhou University, Changzhou, 213164, China

3 MOE Medical Basic Research Innovation Center for Gut Microbiota and Chronic Diseases, Wuxi School of Medicine, Jiangnan university, Wuxi, Jiangsu 214122, China

^#^The authors contributed equally to this work

*Corresponding authors: Bo Zhang, [zhangbo1027@cczu.edu.cn;](mailto:zhangbo1027@cczu.edu.cn;) Ning Sun, [sunning@jiangnan.edu.cn;](mailto:sunning@jiangnan.edu.cn;) Ke-Jia Wu: [kj-wu@jiangnan.edu.cn;](mailto:kj-wu@jiangnan.edu.cn;) Chenwen Shao, [shaochenwen@jiangnan.edu.cn](mailto:shaochenwen@jiangnan.edu.cn)

**
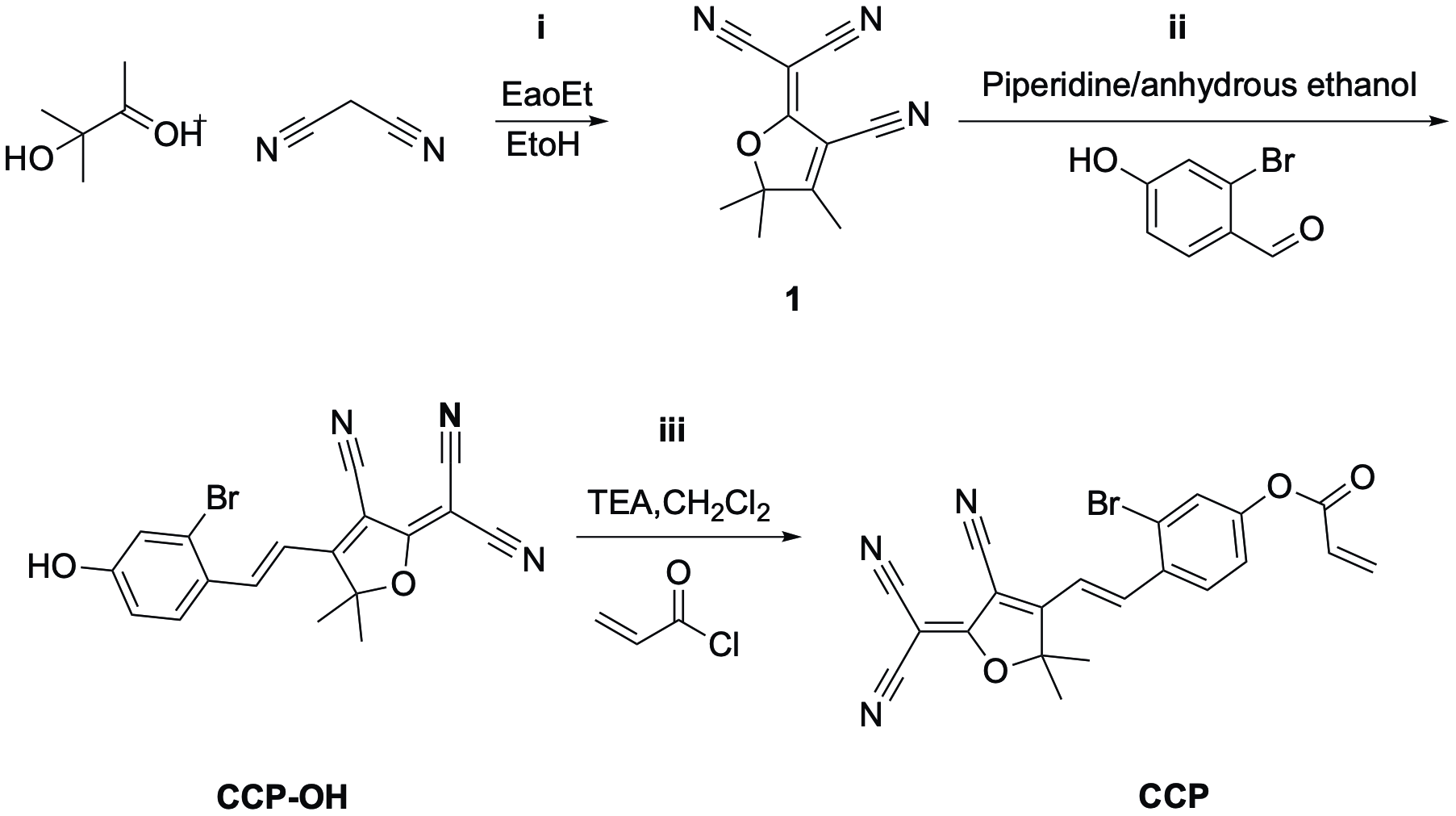
**

**Scheme S1.** Synthetic route of the cysteine-responsive fluorescent probe CCP.


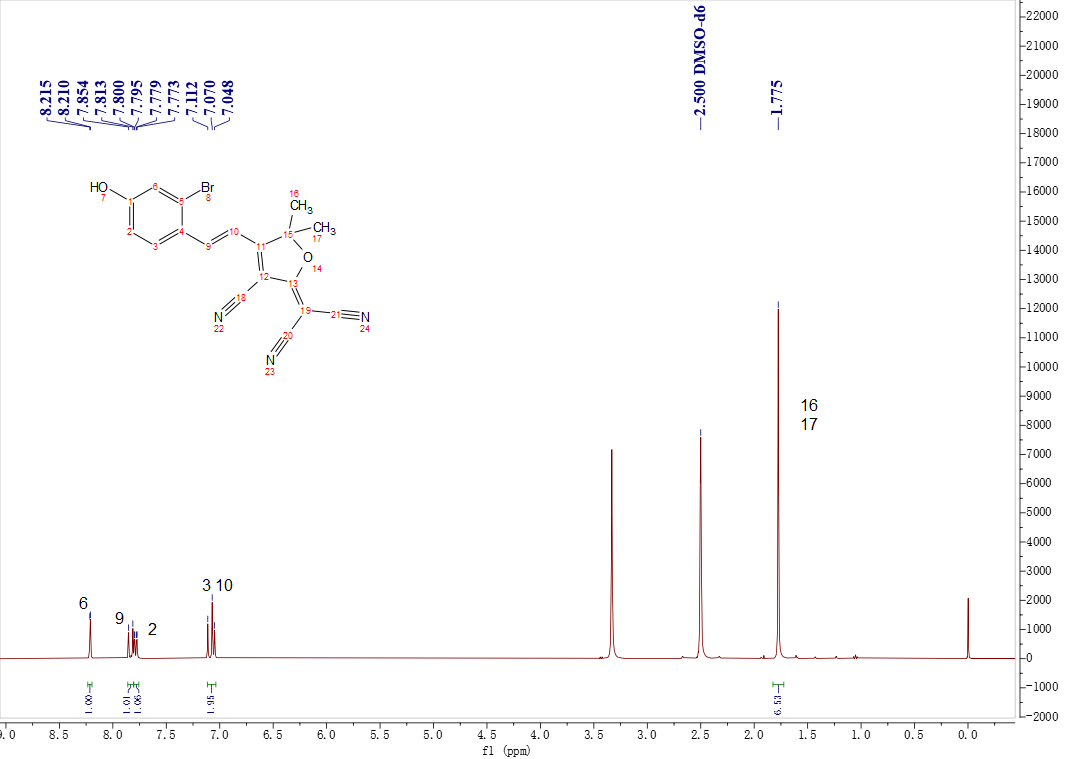


**Figure S1.** ^1^H NMR of CCP-OH (400 MHz, in DMSO-*d_6_*)*.*^1^H NMR (400 MHz, DMSO-*d*_6_) δ 8.21 (d, *J* = 2.1 Hz, 1H, H-6), 7.83 (d, *J* = 16.4 Hz, 1H, H-9), 7.79 (dd, *J* = 8.6, 2.1 Hz, 1H, H-2), 7.14 – 7.03 (m, 2H, H-3, 10), 1.77 (s, 6H, H-16, 17).


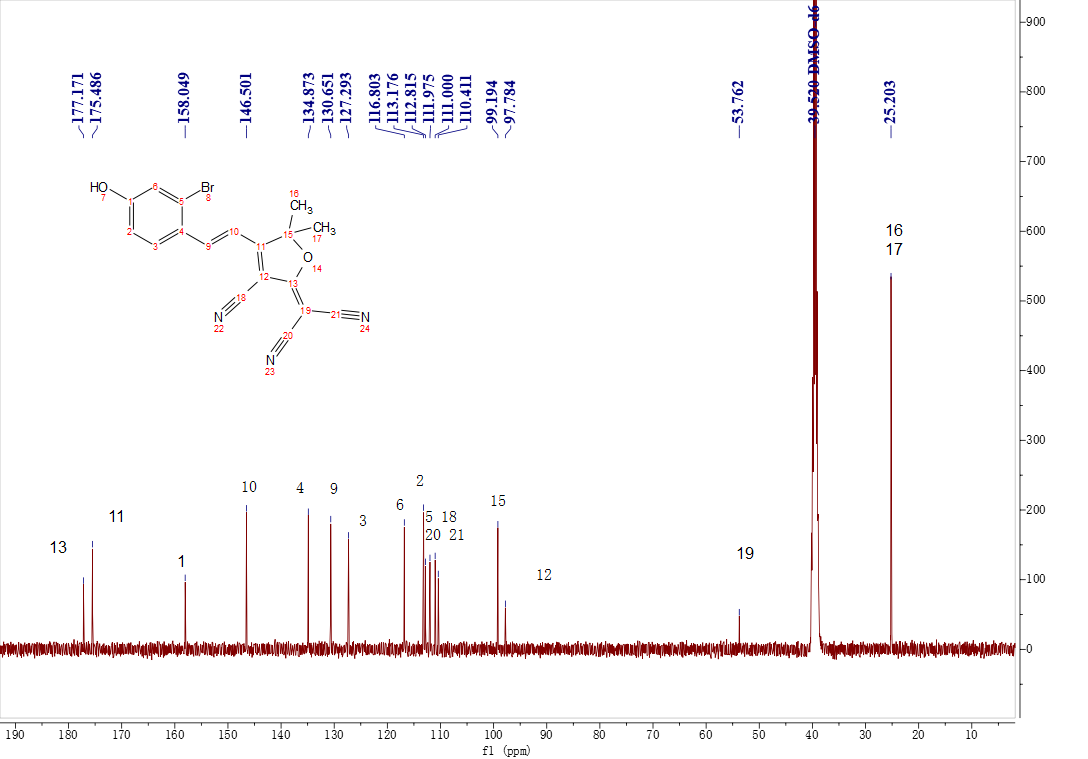


**Figure S2**. ^13^C NMR of CCP-OH (100 MHz, in DMSO-*d_6_*).^13^C NMR (100 MHz, DMSO-*d_6_*) δ 177.17 (C-13), 175.49 (C-11), 158.05 (C-1), 146.50 (C-10), 134.87 (C-4), 130.65 (C-9), 127.29 (C-3), 116.80 (C-6), 113.18 (C-2), 112.81 (C-5), 111.98 (C-18), 111.00 (C-20), 110.41 (C-21), 99.19 (C-15), 97.78 (C-12), 53.76 (C-19), 25.20 (C-16, 17).


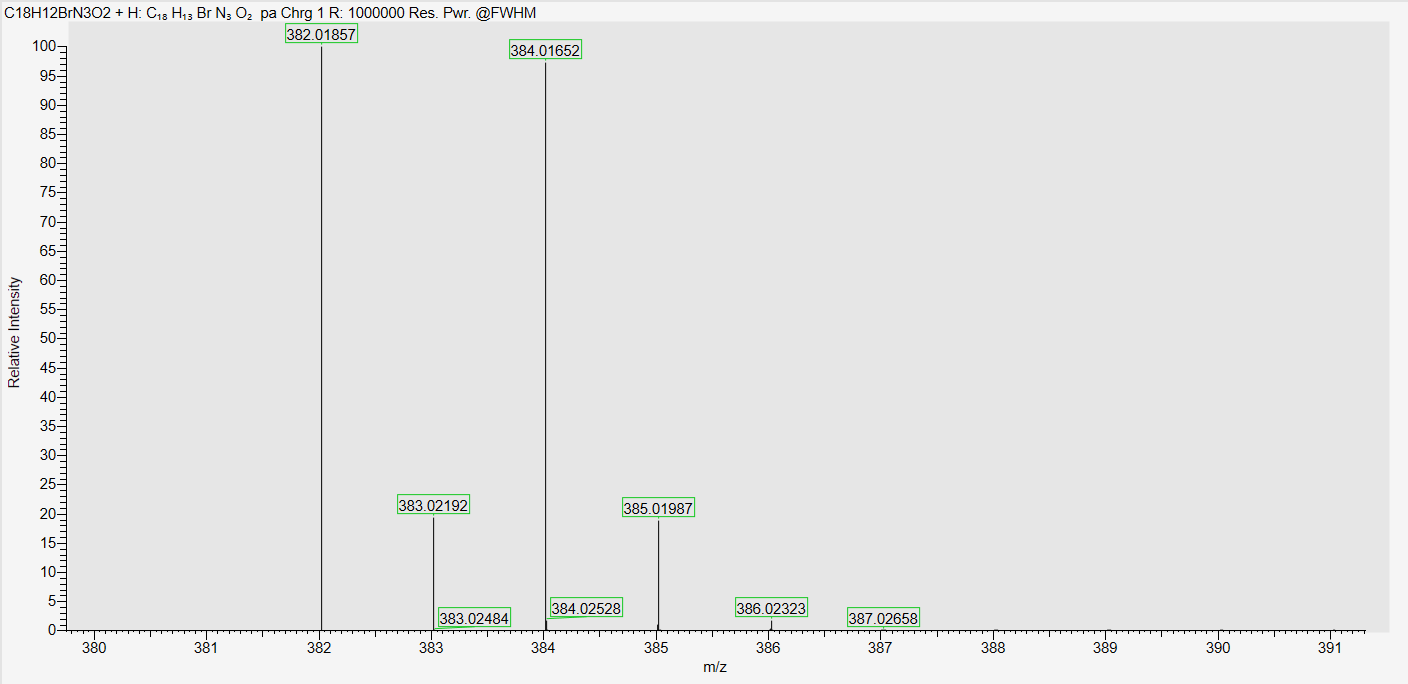


**Figure S3**. The HRMS (ESI, Q Exactive™ Plus) spectrum of CCP-OH (calculated for C_18_H_13_BrN_3_O_2_ [M+H]^+^ 382.0186, found 382.01857).


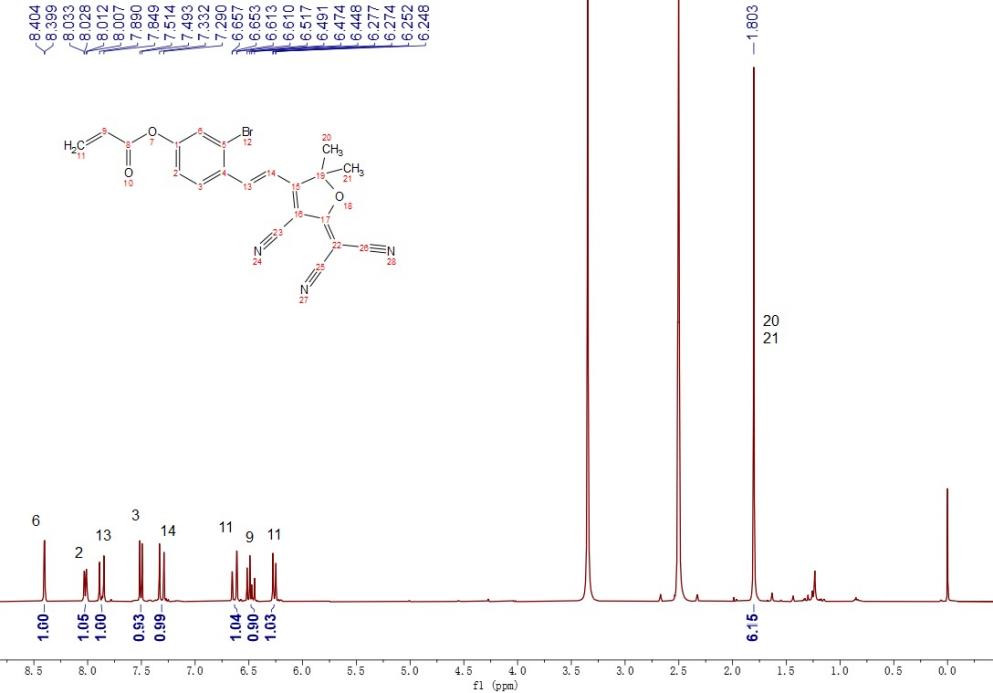


**Figure S4.** ^1^H NMR of CCP (400 MHz, in DMSO-*d_6_*)*.*^1^H NMR (400 MHz, DMSO-*d_6_*) δ 8.40 (d, J = 2.0 Hz, 1H, H-6), 8.02 (dd, J = 8.5, 2.0 Hz, 1H, H-2), 7.87 (d, J = 16.5 Hz, 1H, H-13), 7.50 (d, J = 8.5 Hz, 1H, H-3), 7.31 (d, J = 16.5 Hz, 1H, H-14), 6.63 (dd, J = 17.2, 1.3 Hz, 1H, H-11), 6.48 (dd, J = 17.2, 10.4 Hz, 1H, H-9), 6.26 (dd, J = 10.4, 1.3 Hz, 1H, H-11), 1.80 (s, 6H, H-20, 21).


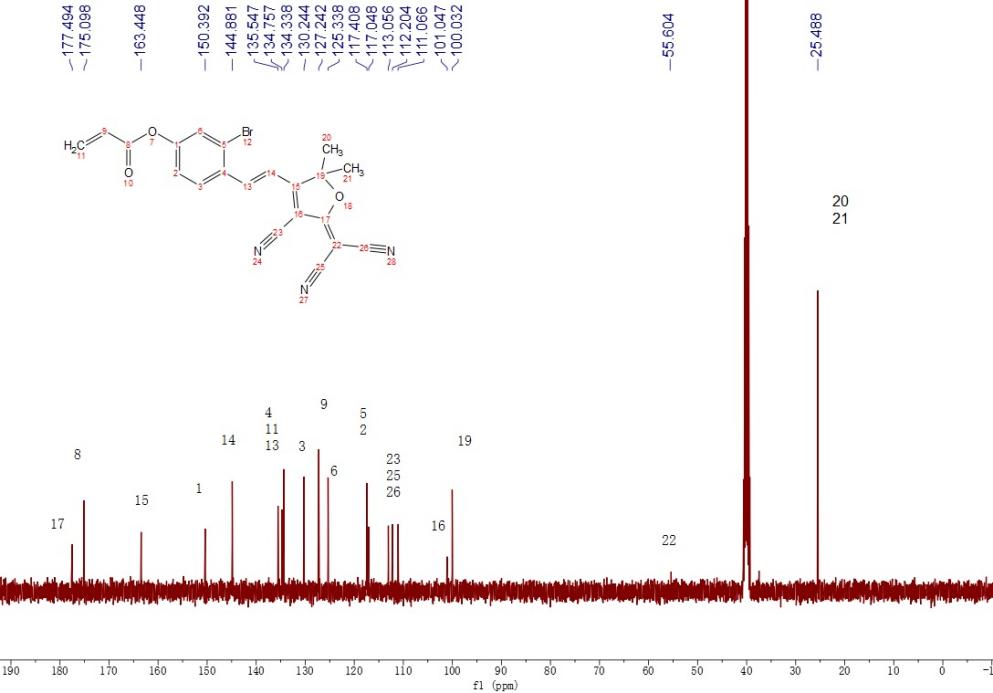


**Figure S5**. ^13^C NMR of CCP (100 MHz, in DMSO-*d6*).^13^C NMR (100 MHz, DMSO-*d6*) δ 177.49 (C-17), 175.10 (C-8), 163.45 (C-15), 150.39 (C-1), 144.88 (C-14), 135.55 (C-11), 134.76 (C-4), 134.34 (C-13), 130.24 (C-3), 127.24 (C-9), 125.34 (C-6), 117.41 (C-2), 117.05 (C-5), 113.06 (C-23), 112.20 (C-25), 111.07 (C-26), 101.05 (C-16), 100.03 (C-19), 55.60 (C-22), 25.49 (C-20, 21).


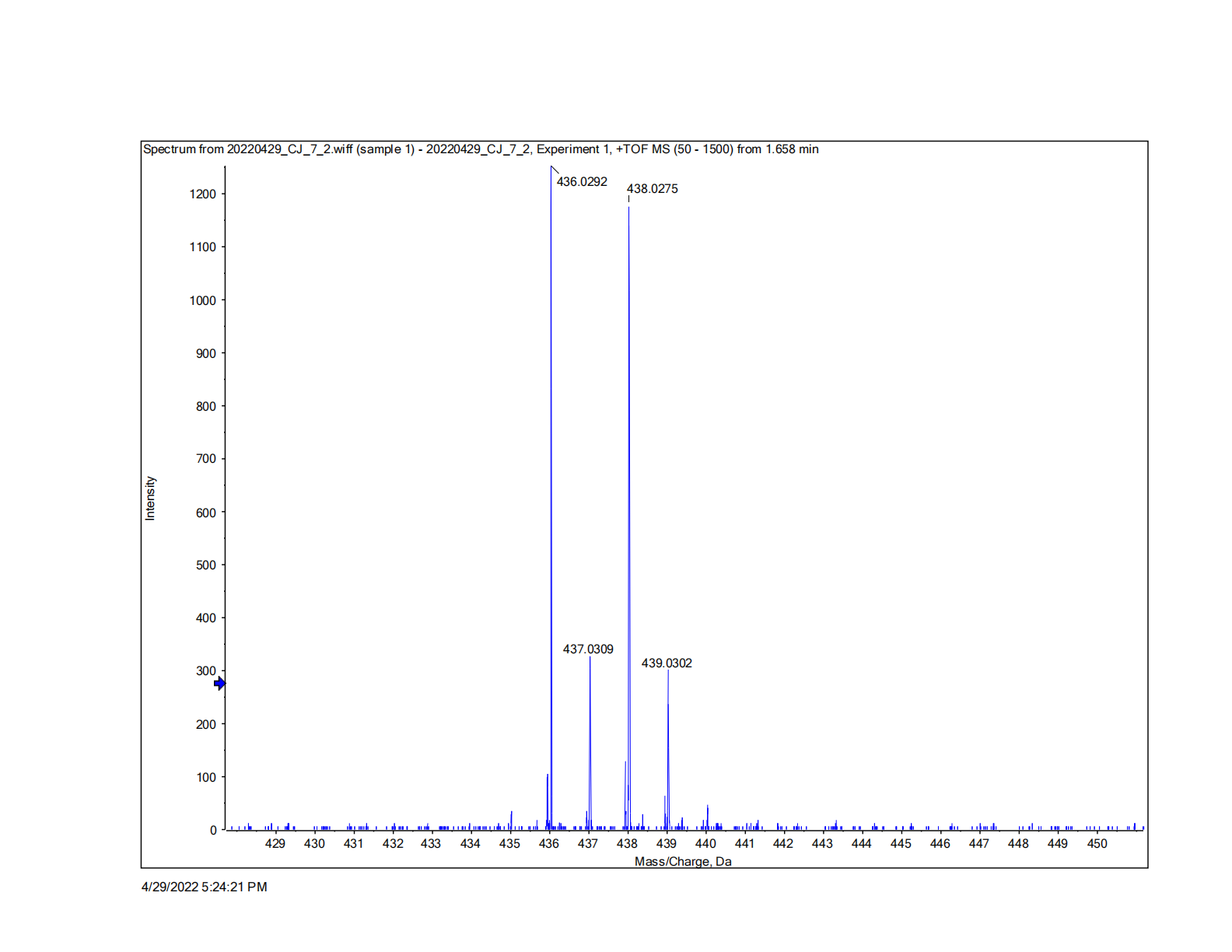


**Figure S6**. The TOF-MS spectrum of CCP (calculated for C_21_H_15_BrN_3_O_3_ [M+H]^+^ 436.0291, found 436.0292).


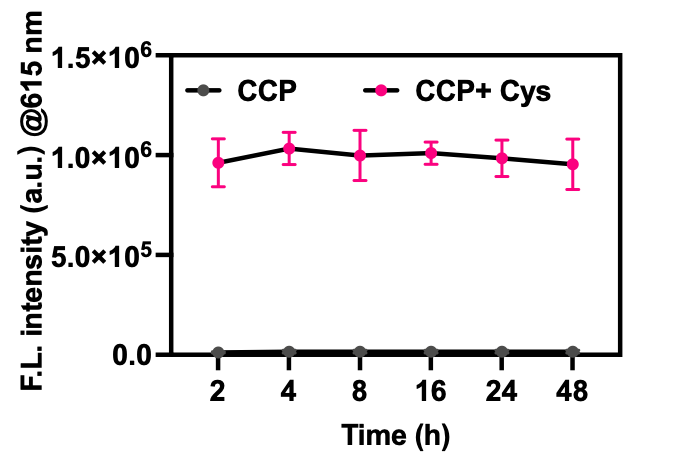


**Figure S7**. Photostability and signal stability of CCP. Time-dependent fluorescence intensity changes of CCP under physiological conditions in the absence of Cys, indicating good probe stability with minimal signal drift. Data are shown as mean ± SD (n = 3).


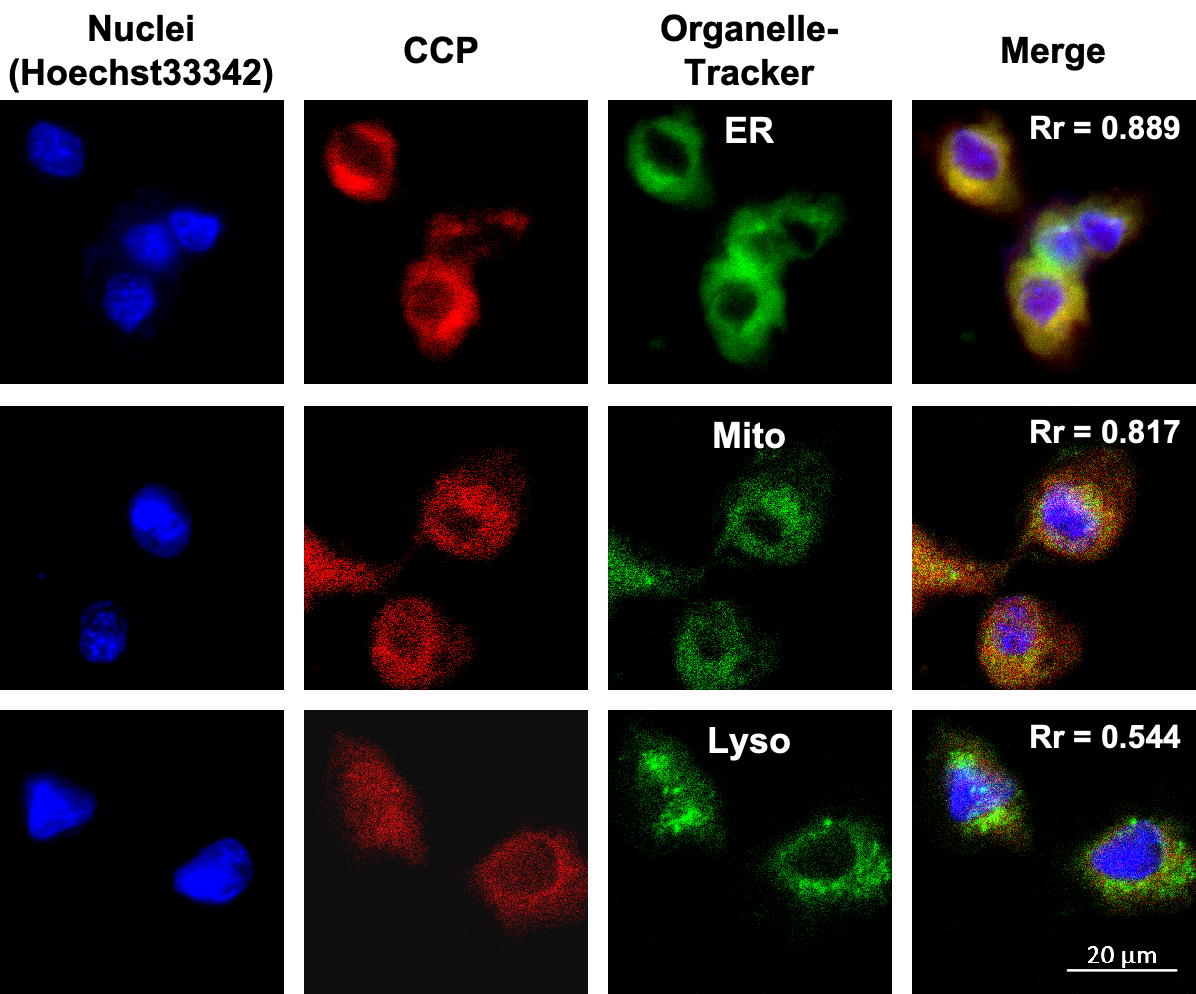


**Figure S8.** Confocal co-localization of CCP with organelle markers. Cells were stained using Beyotime organelle staining kits (Cat. No. C1047, C1048, and C1042, as indicated) and imaged by confocal microscopy. CCP was detected in the red channel with excitation at 565 nm and emission collected at 590–640 nm. Representative images show nuclei (blue), organelle tracker signal (green), CCP (red), and merged images (right). Pearson’s correlation coefficient (Rr) for CCP versus the organelle marker is shown on the merged images. Scale bar, 20 μm


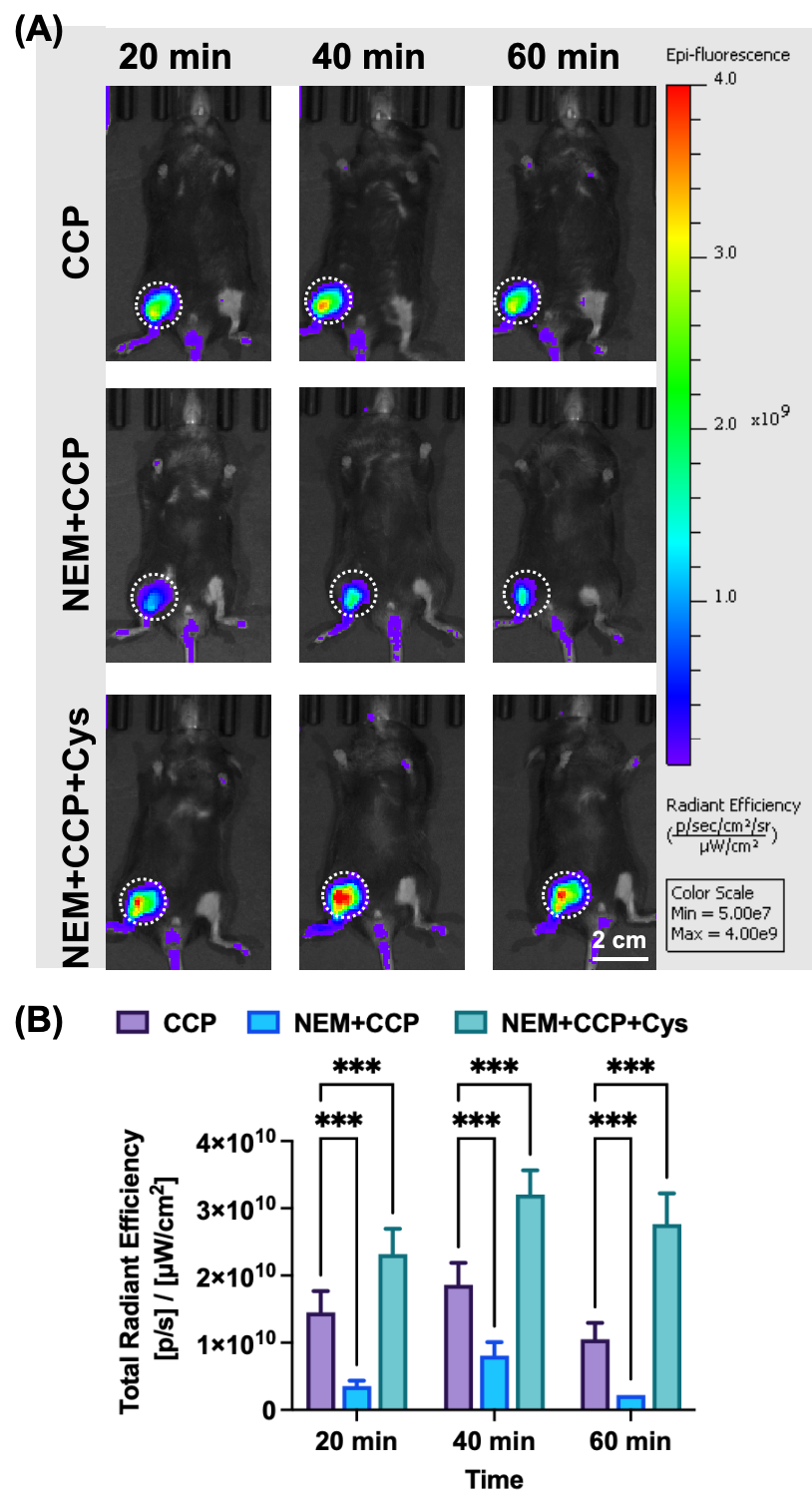


**Figure S9. In vivo fluorescence imaging of the subcutaneous hindlimb signal under thiol blockade and cysteine rescue.** (A) Representative epi-fluorescence images of mice acquired at 20, 40 and 60 min after hindlimb subcutaneous administration of CCP (CCP), N-ethylmaleimide (NEM) pretreatment followed by CCP (NEM+CCP), or NEM pretreatment followed by CCP with exogenous cysteine supplementation (NEM+CCP+Cys). Dashed circles indicate the region of interest (ROI) used for quantification. (B) ROI-based radiant efficiency quantification shown as mean ± SD. (n = 5 mice per group). Scale bar, 2 cm.


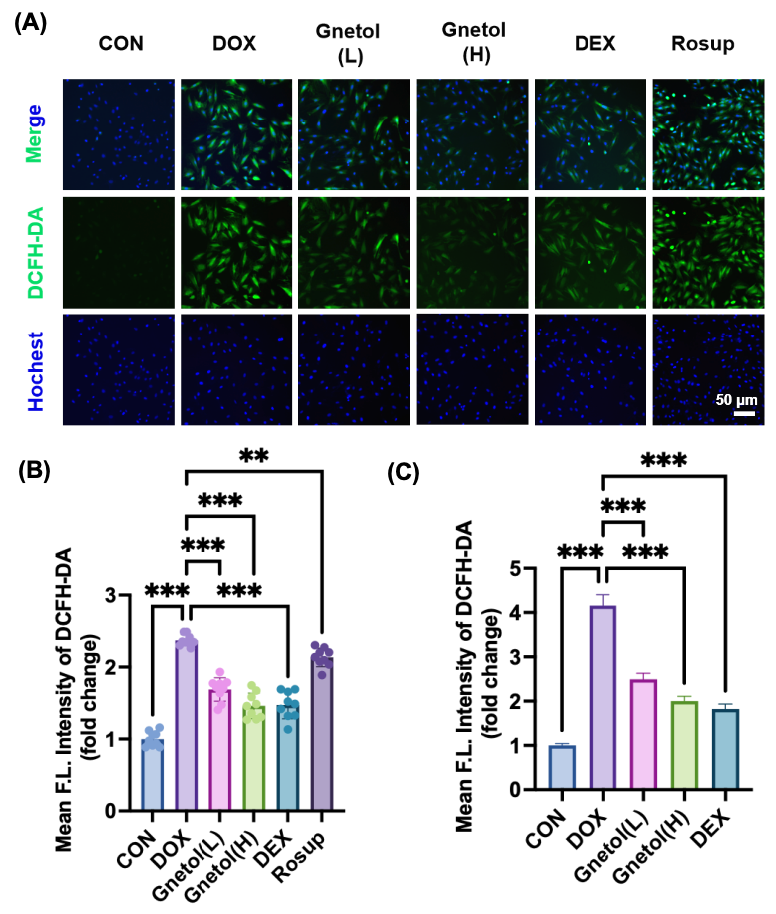


**Figure S10.** DCFH-DA–based detection of intracellular ROS in DOX-injured cells and its attenuation by Gnetol. (A) Representative fluorescence images of cells stained with DCFH-DA (green) to indicate total intracellular reactive oxygen species (ROS) and Hoechst 33342 (blue) to label nuclei under the indicated treatments (CON, DOX, Gnetol (L), Gnetol (H), DEX, and Rosup). Merged images are shown in the top row. (B) Quantification of DCFH-DA mean fluorescence intensity presented as fold change relative to CON. Scale bar, 50 μm. (C) ROS levels were quantified by flow cytometry following DCFH-DA staining. Statistical significance is denoted as *P < 0.05, **P < 0.01, ***P < 0.001.


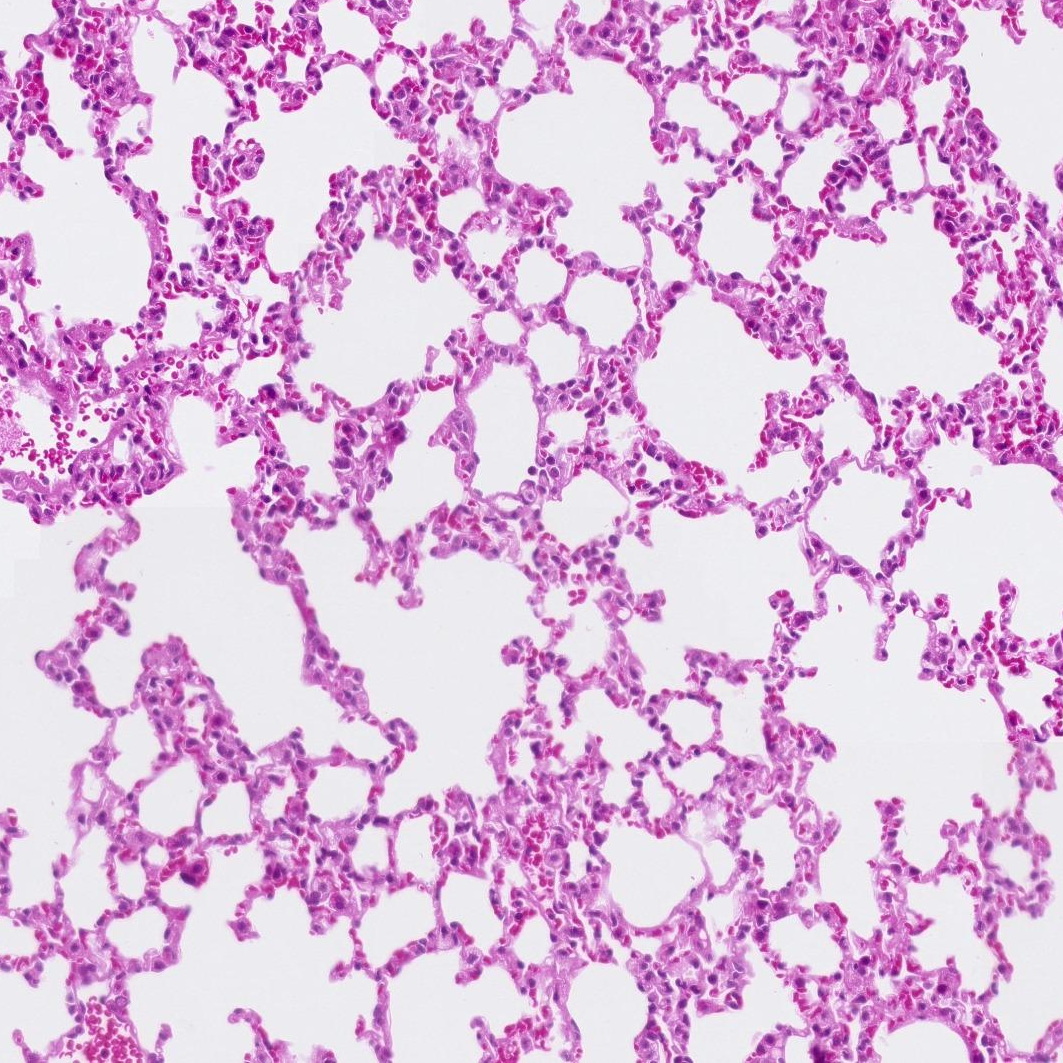

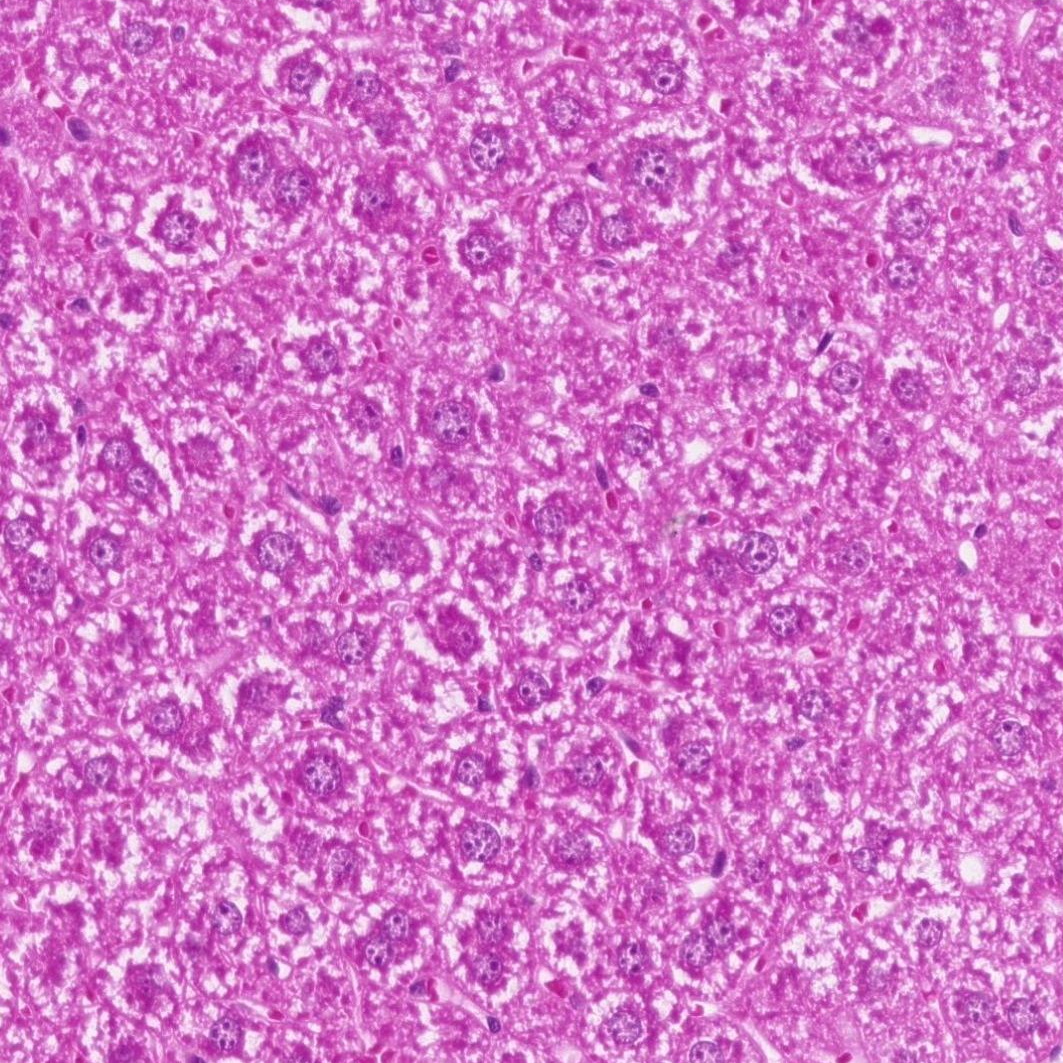

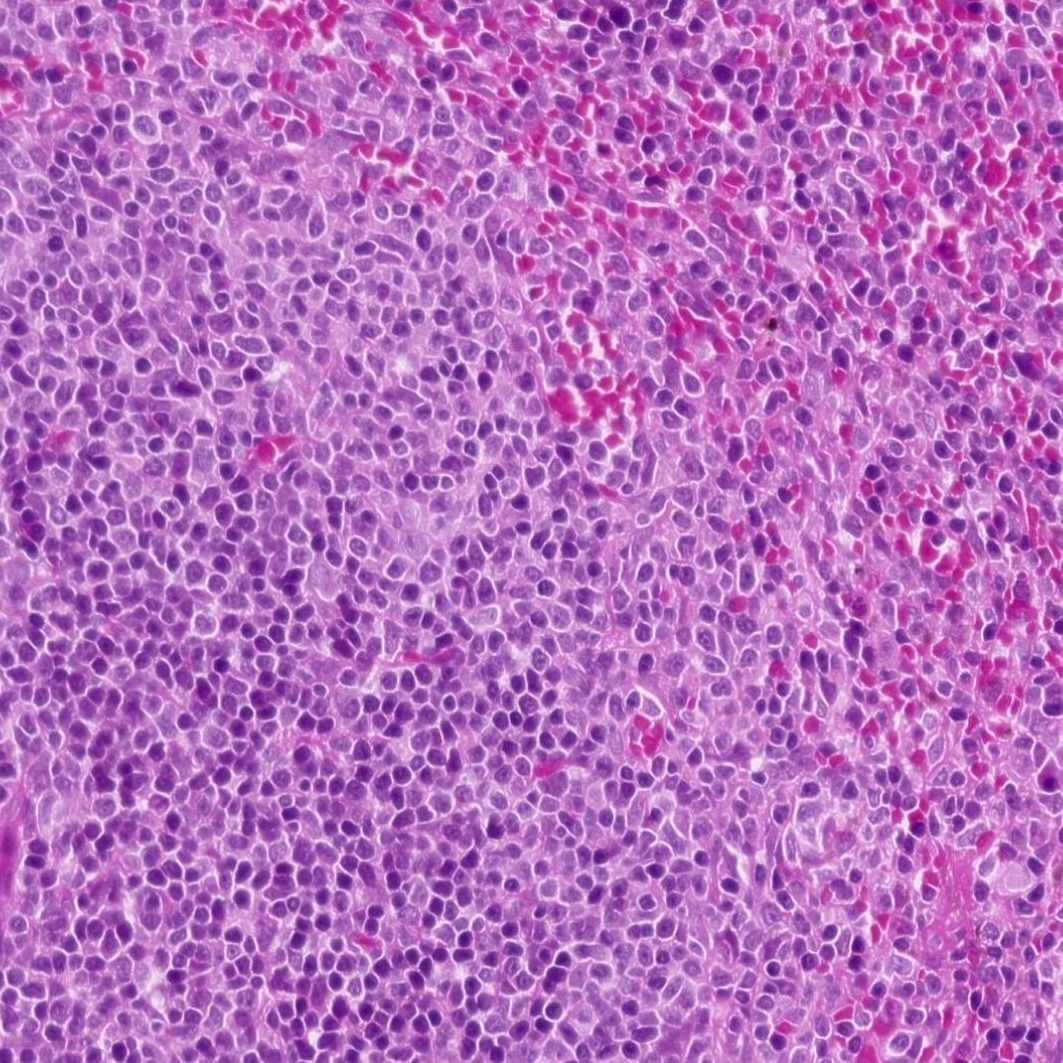

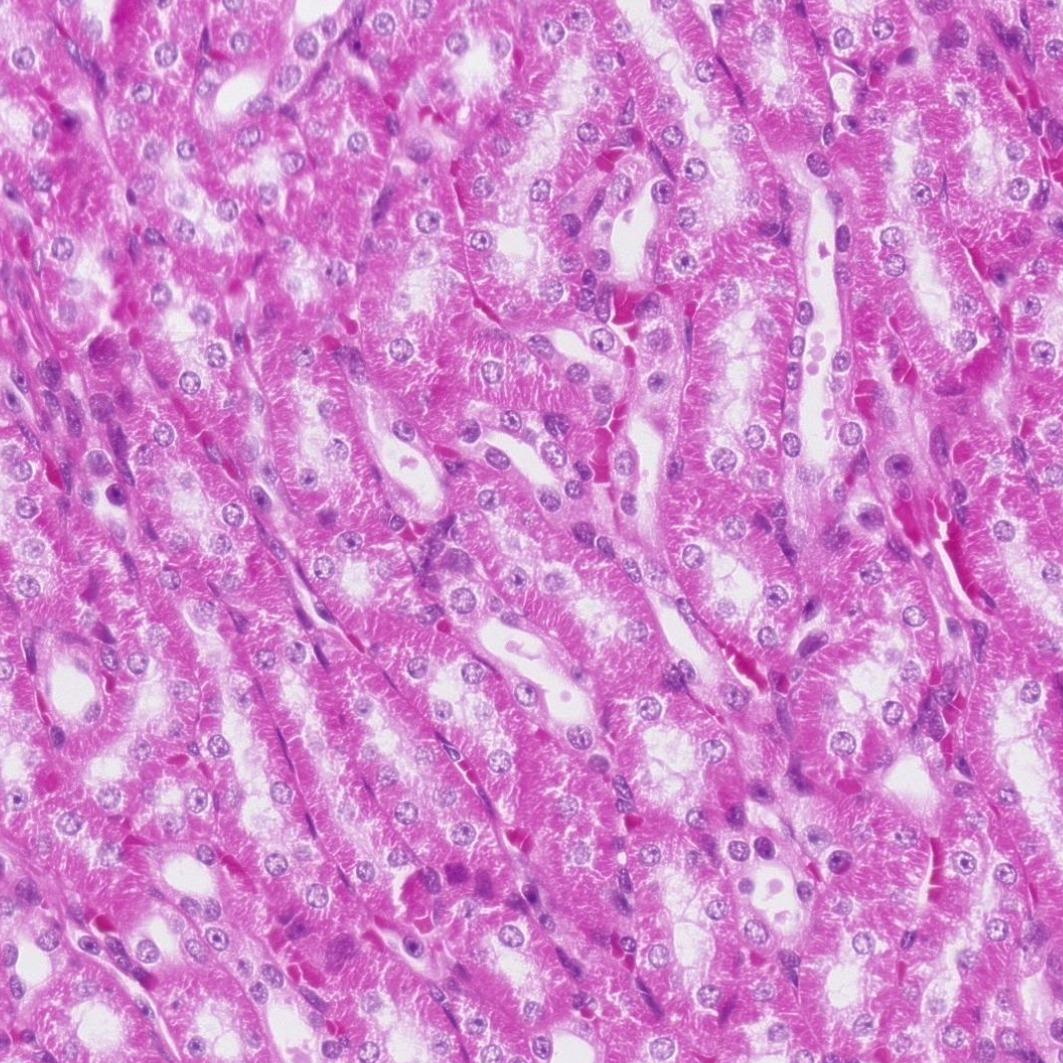

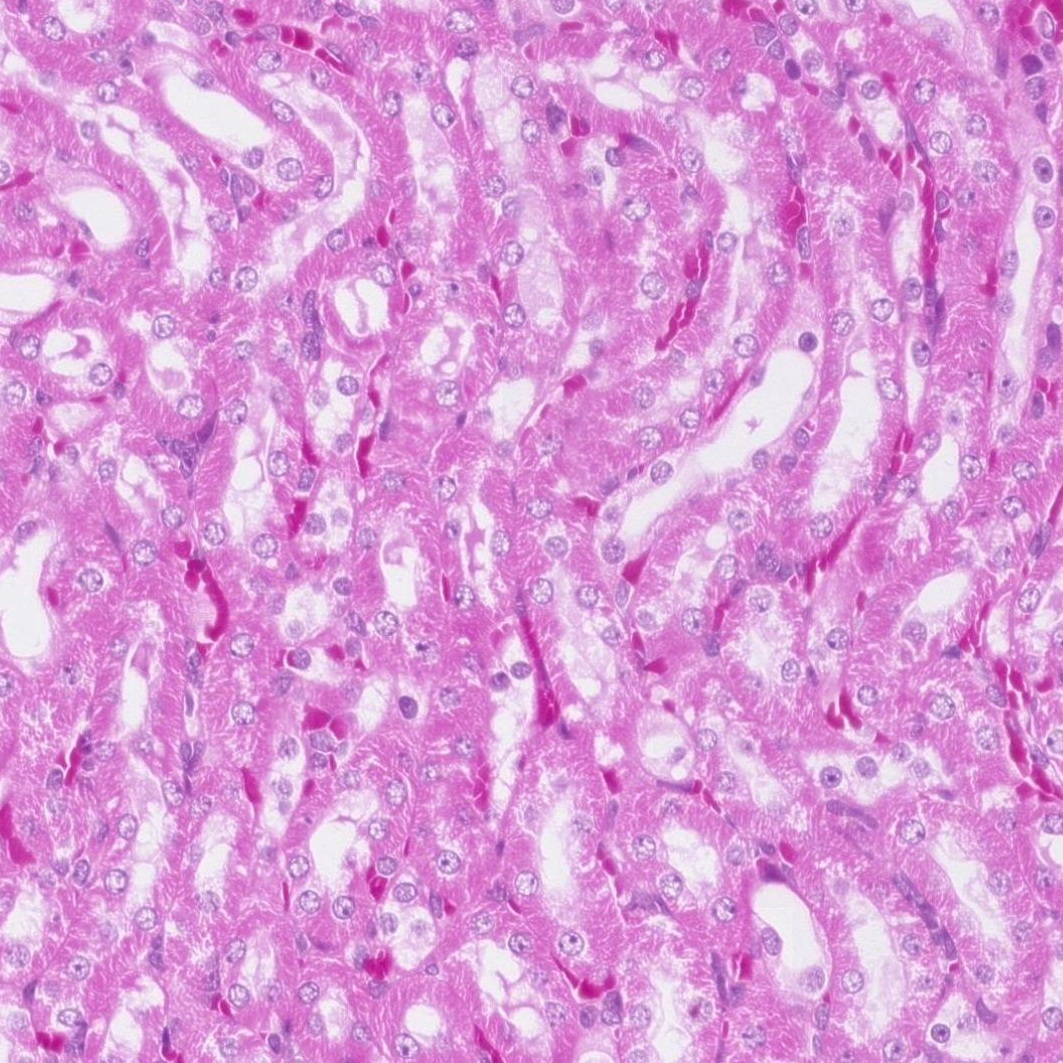

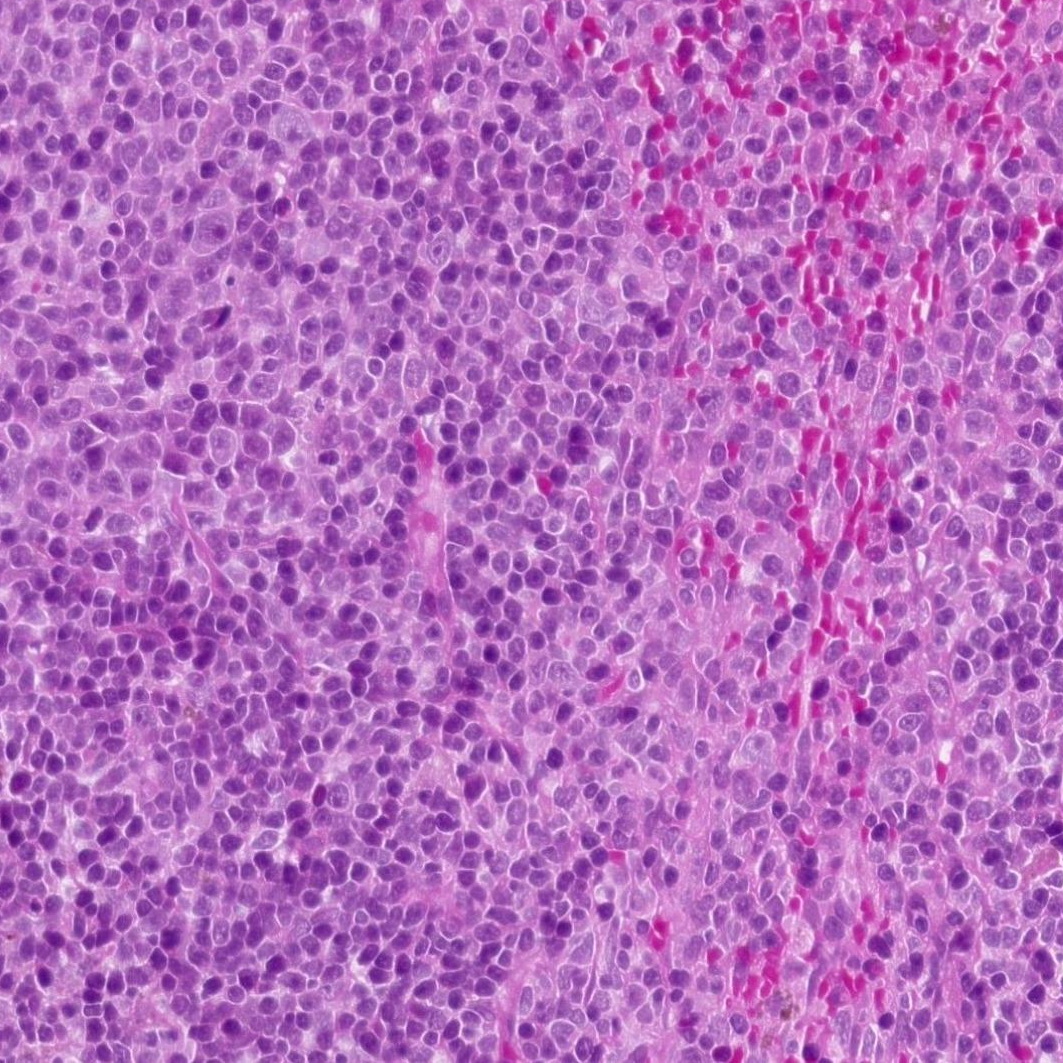

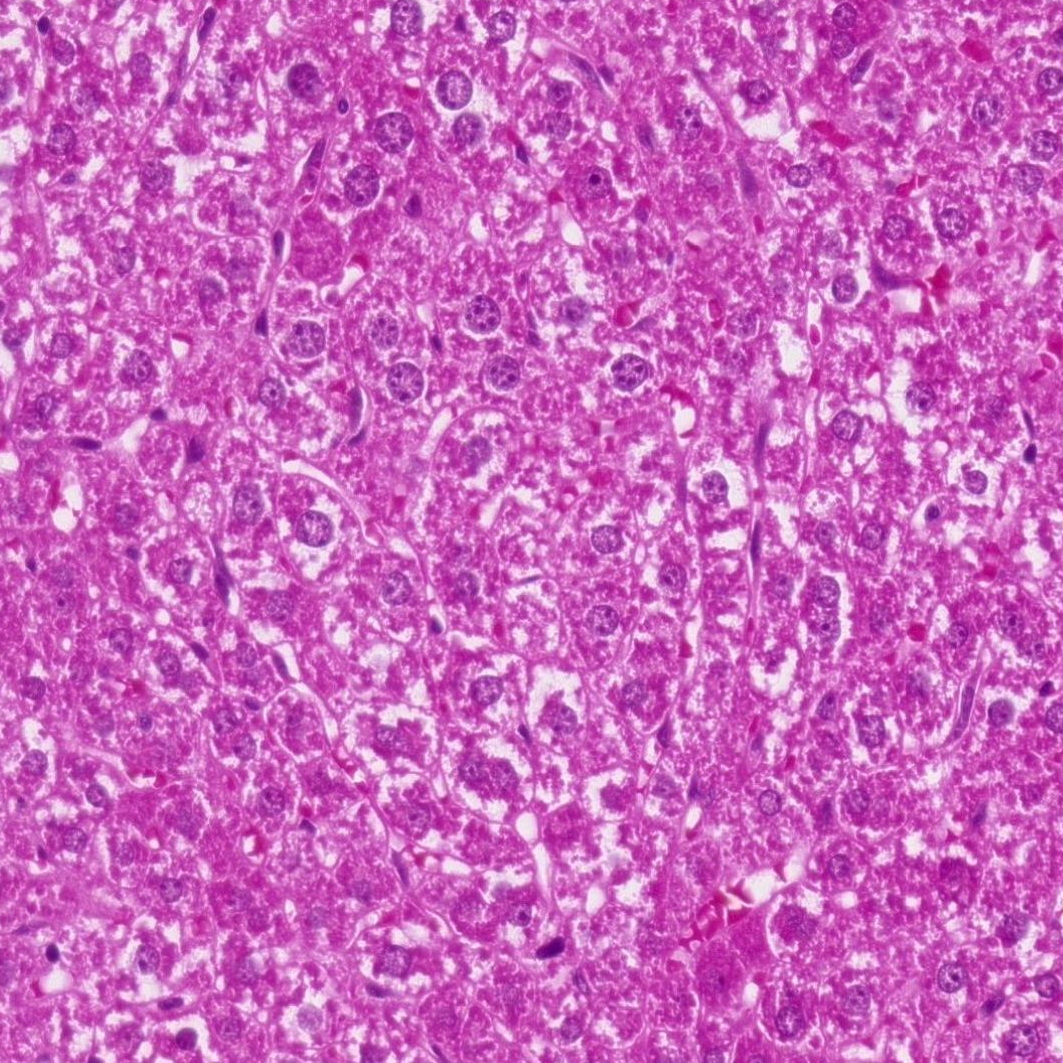

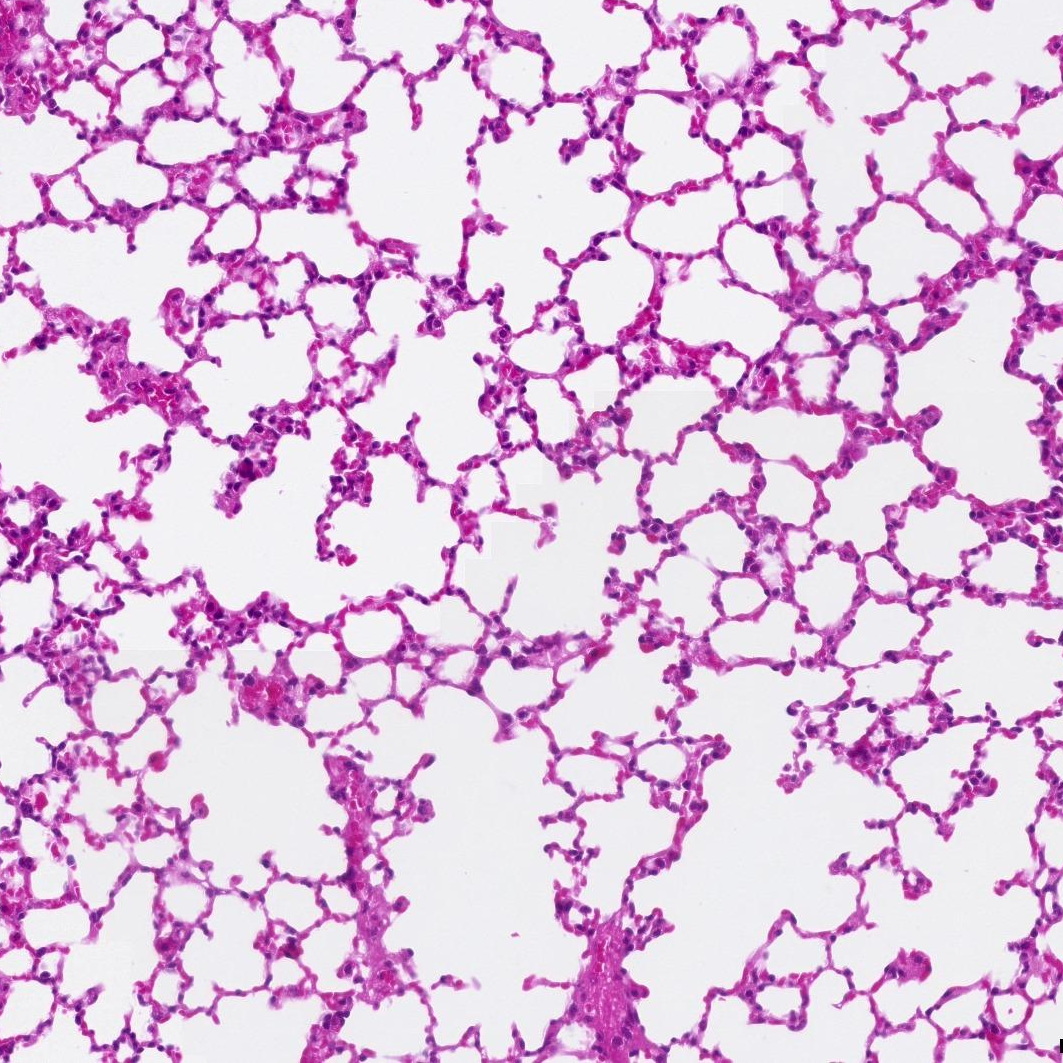

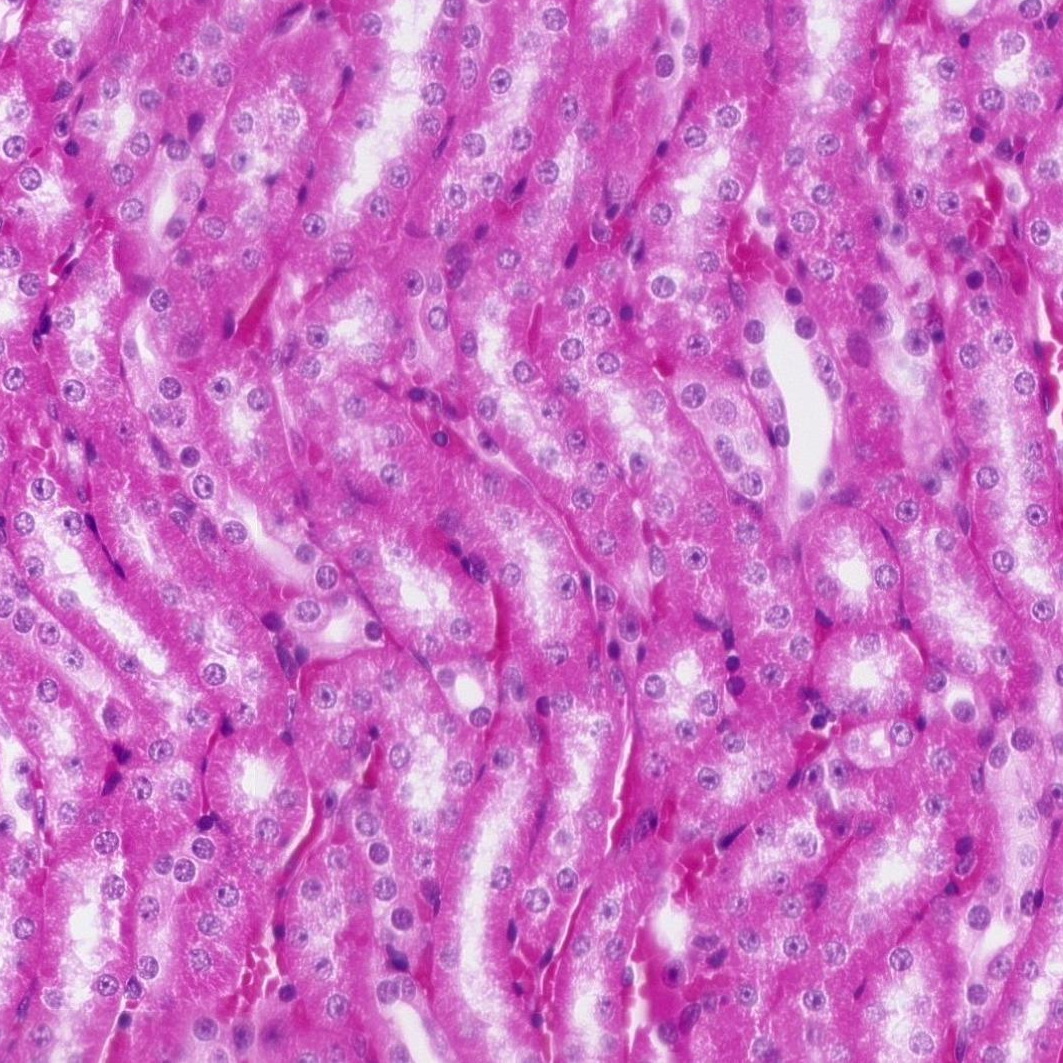

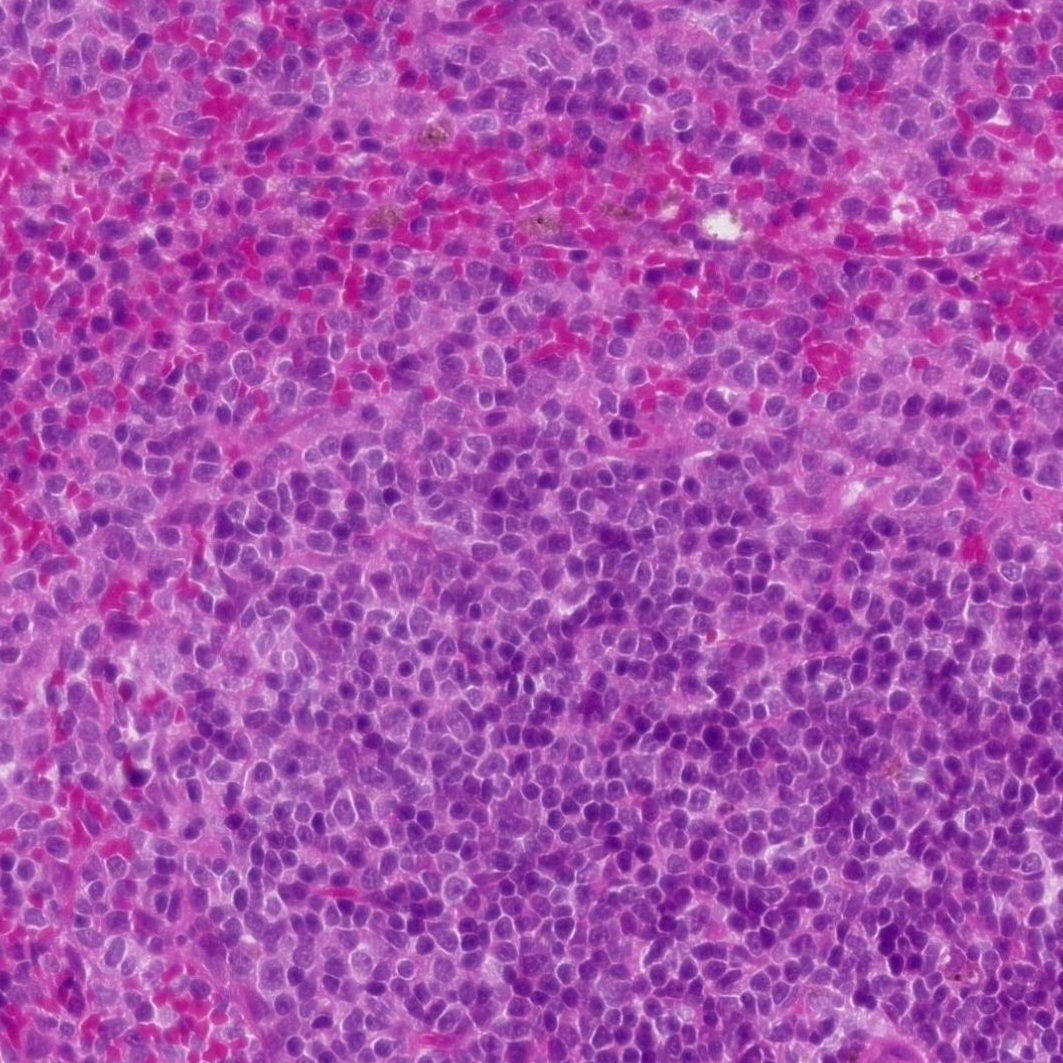

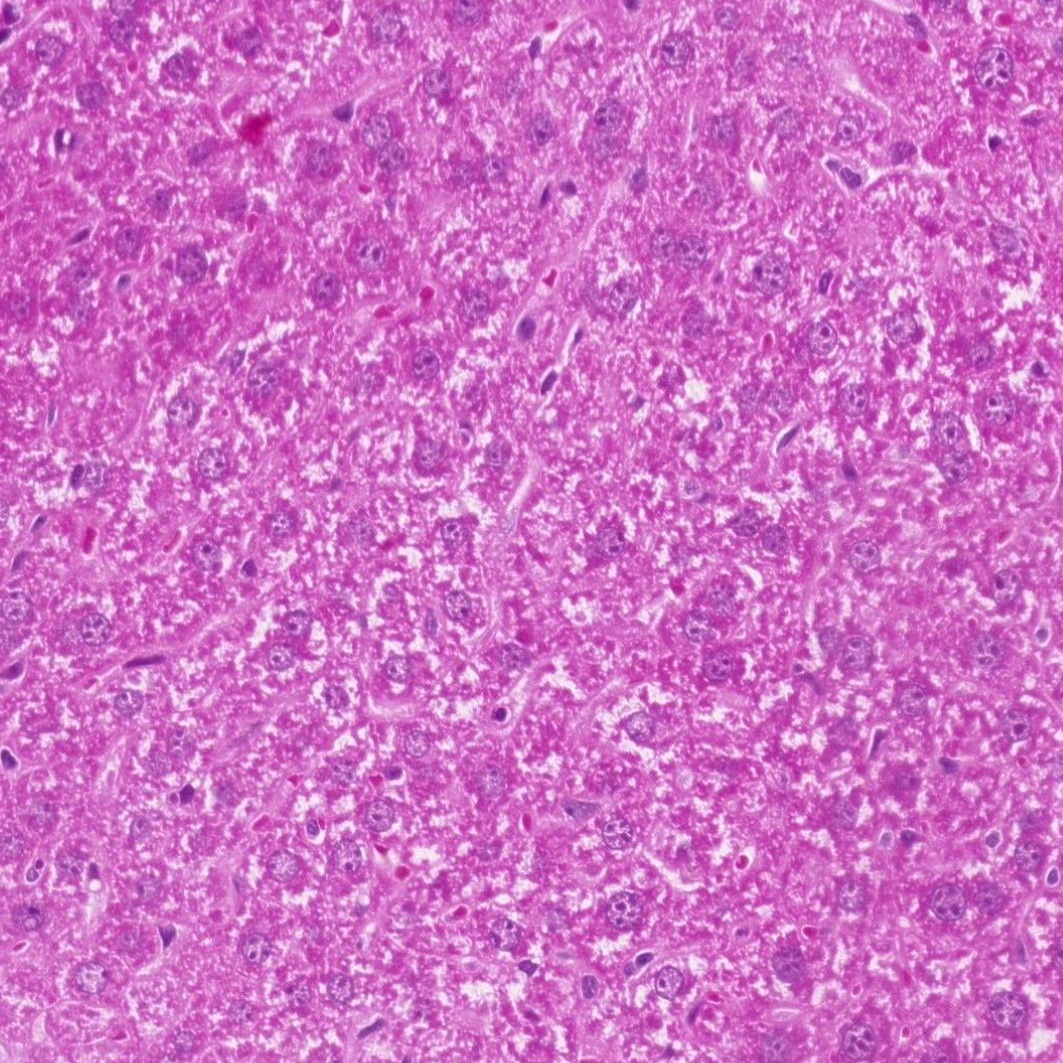

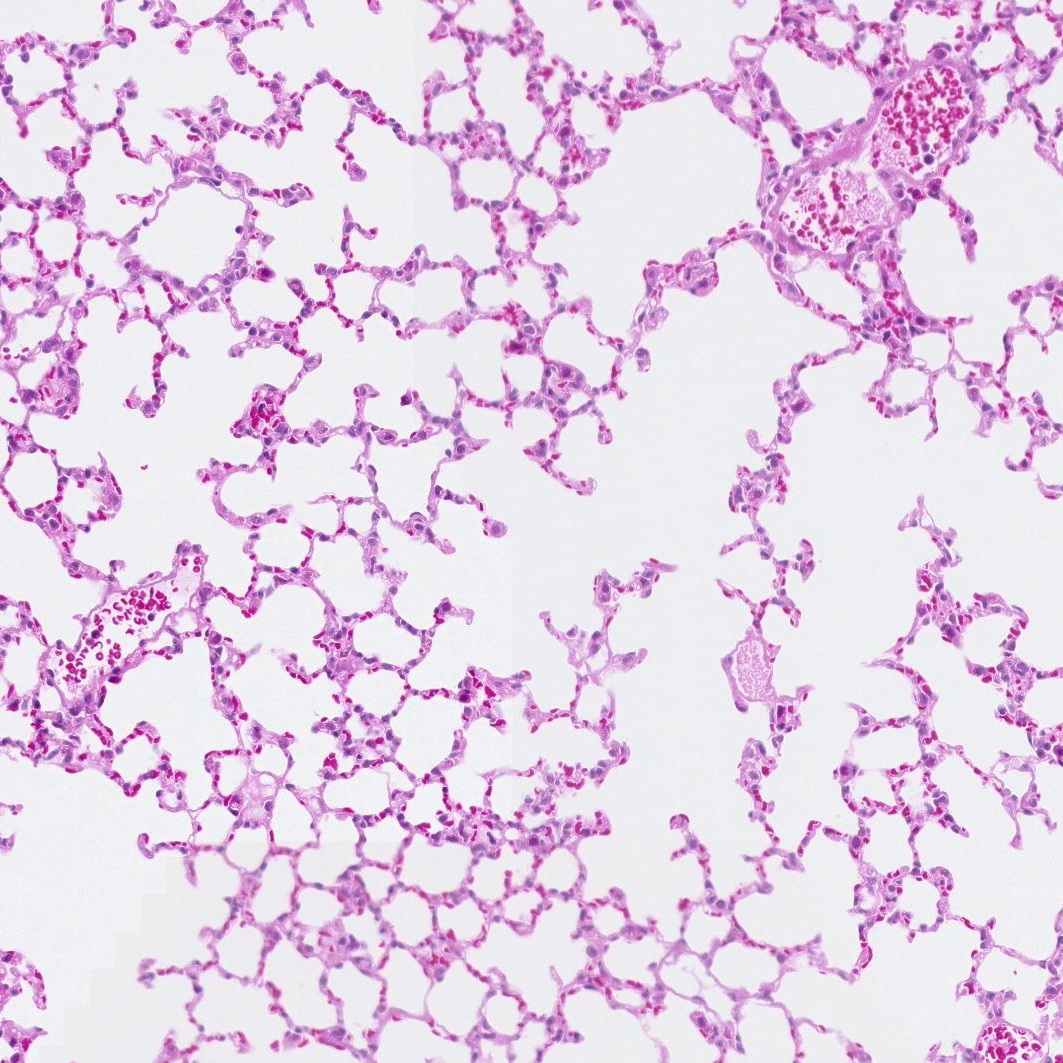

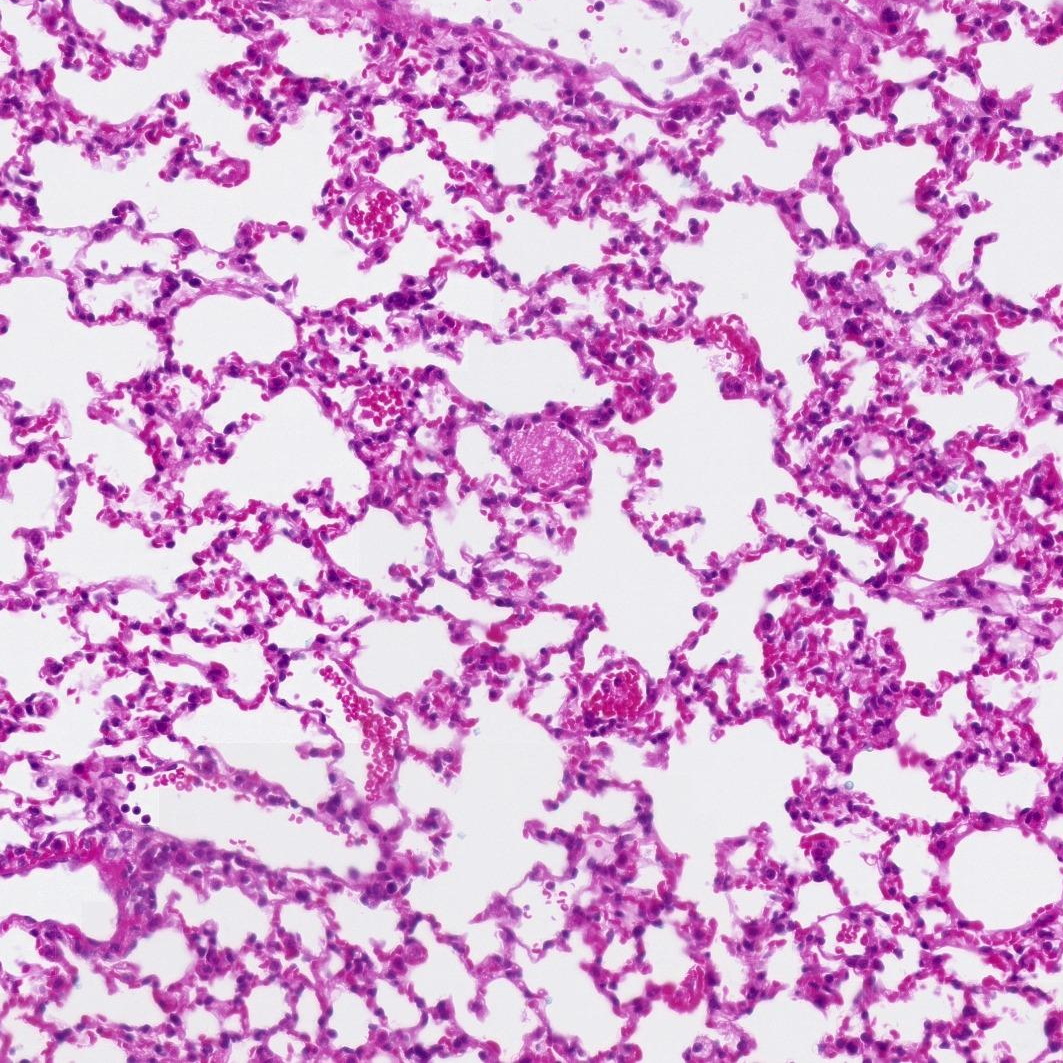

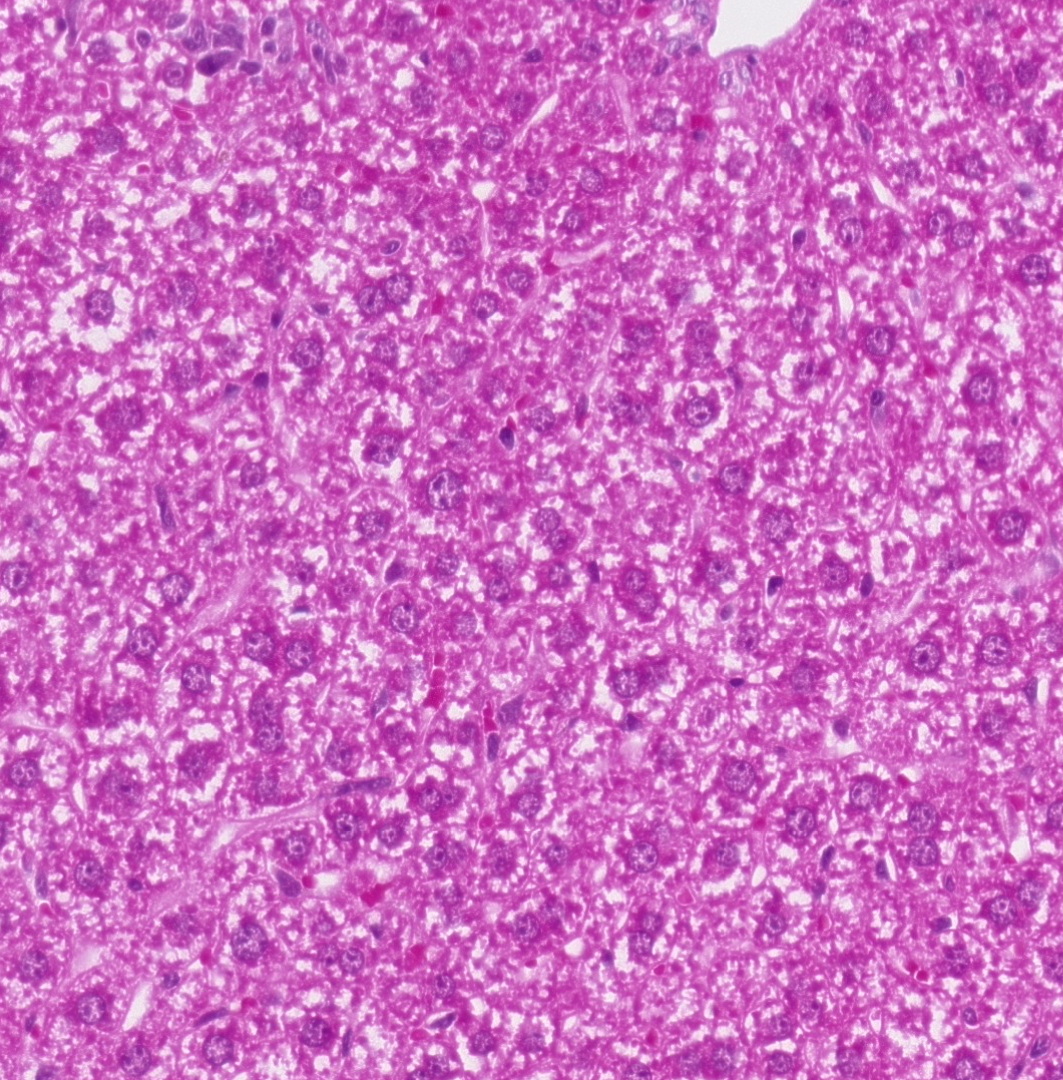

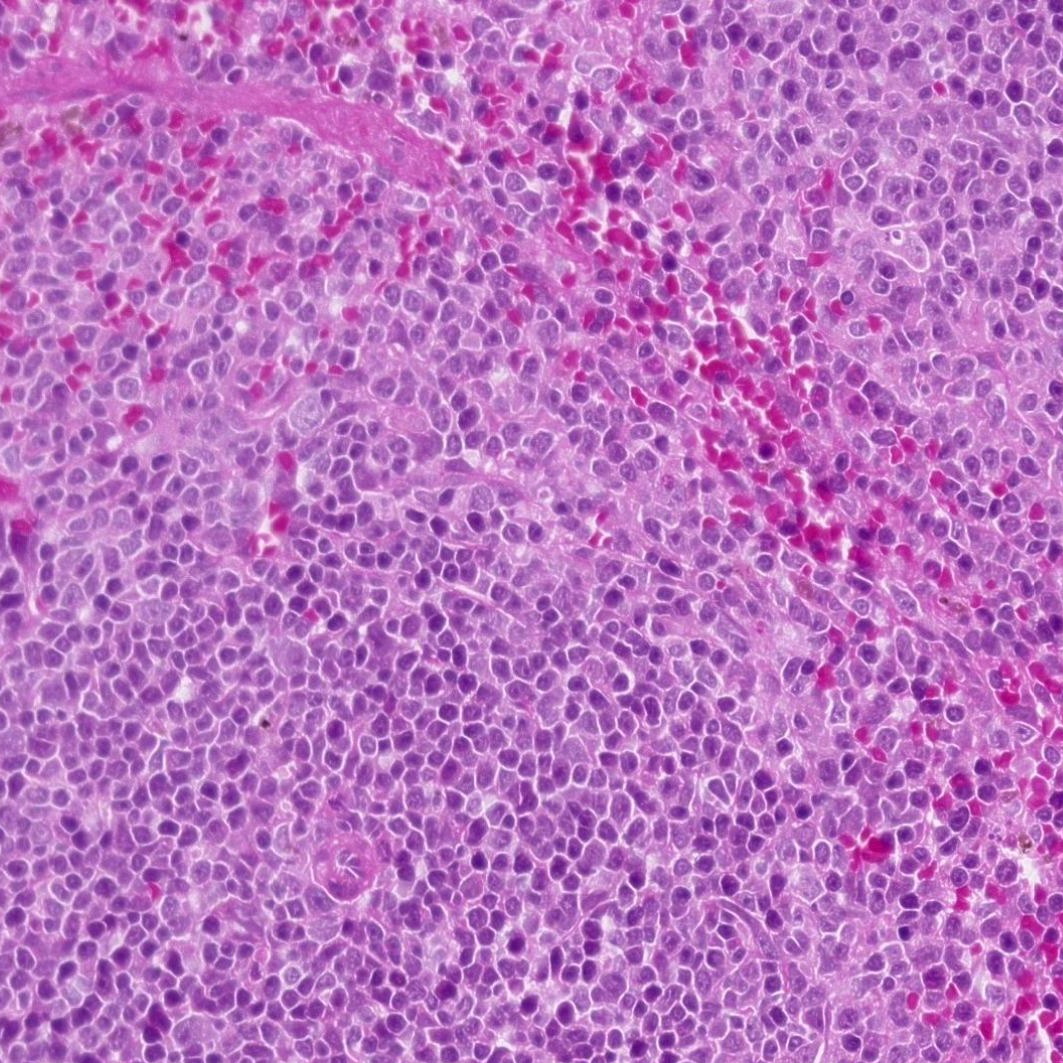

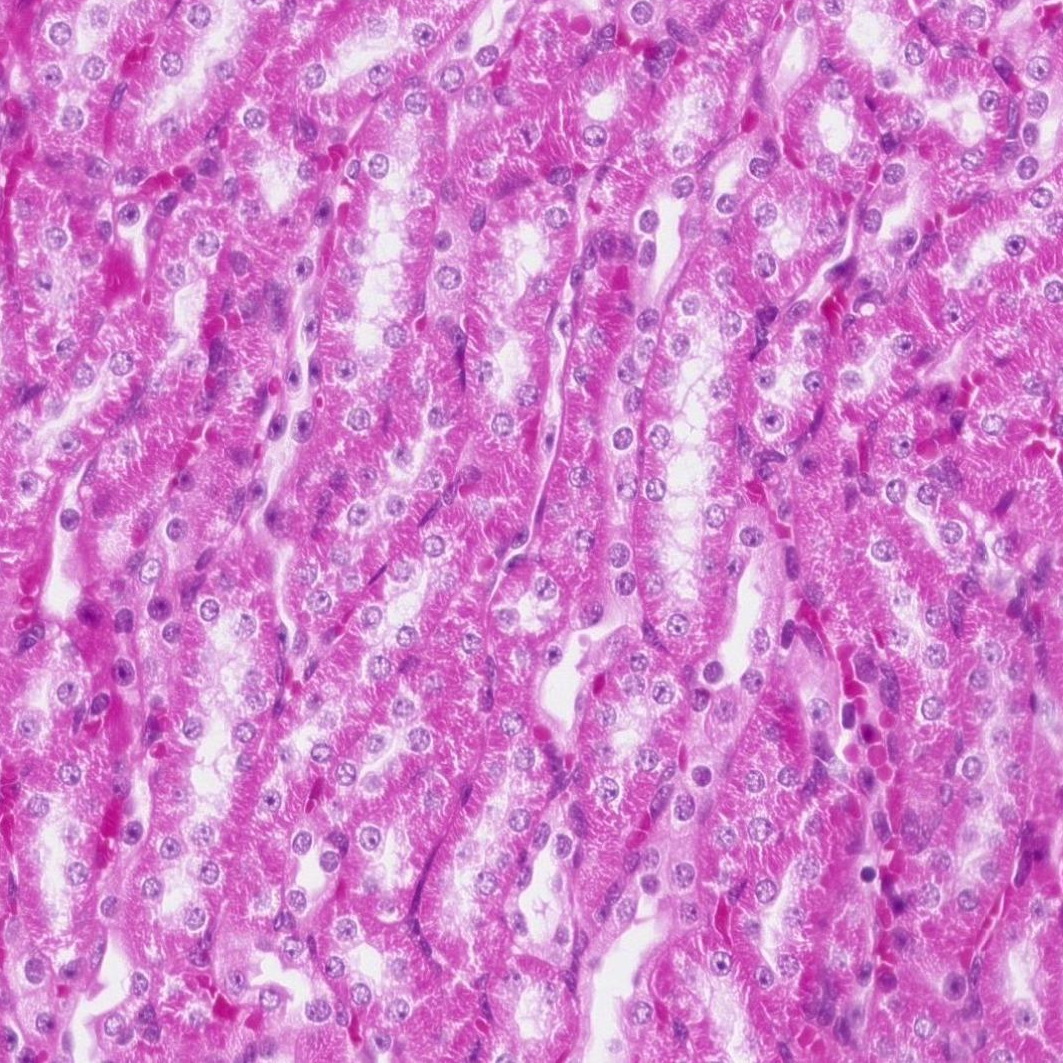

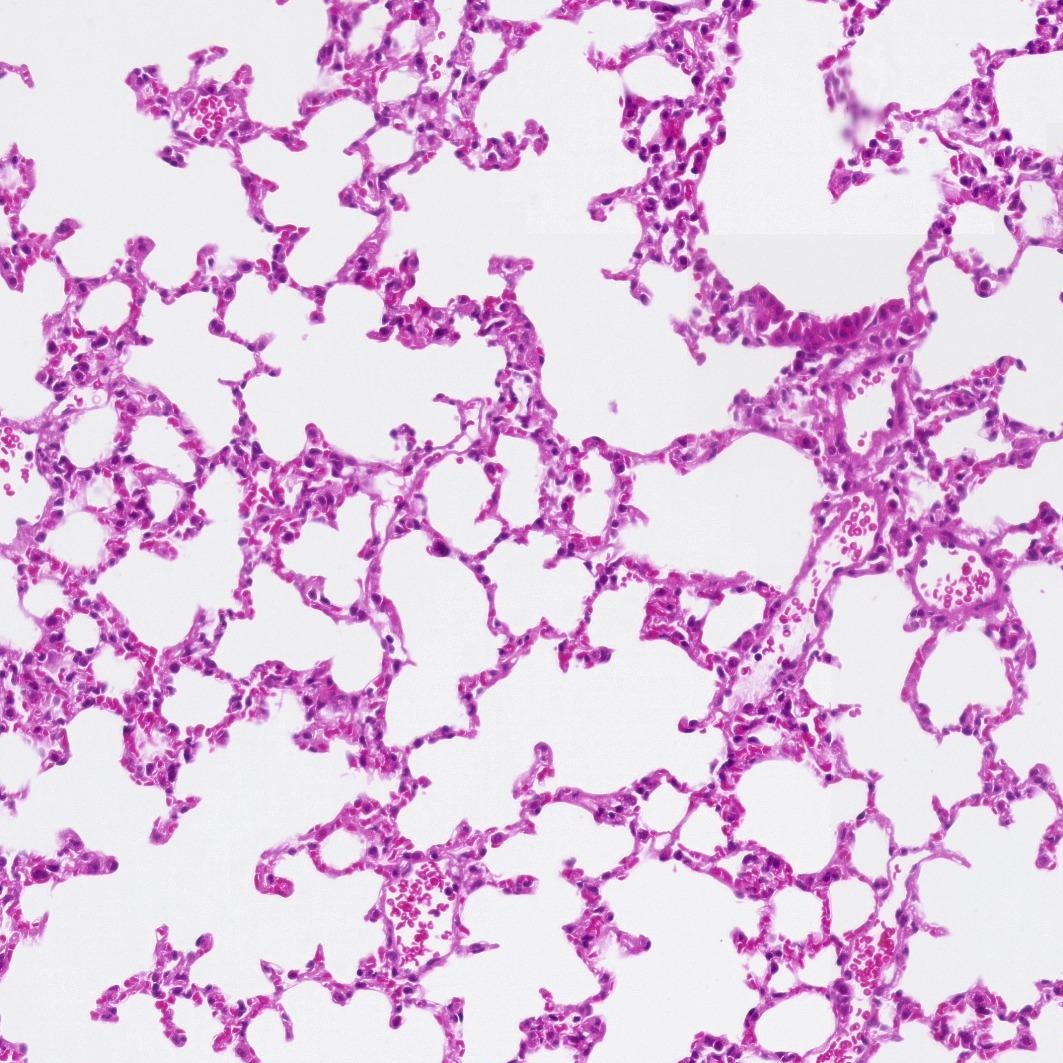

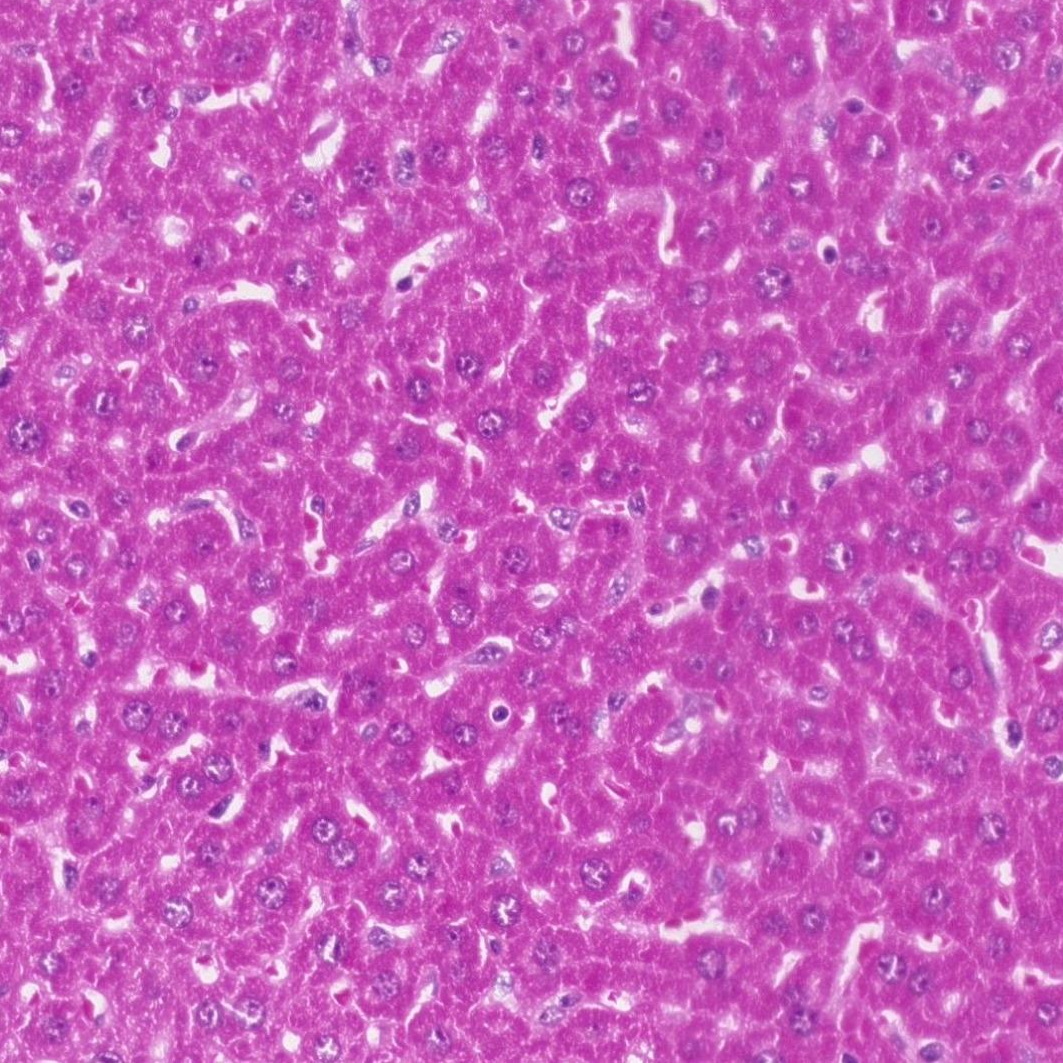

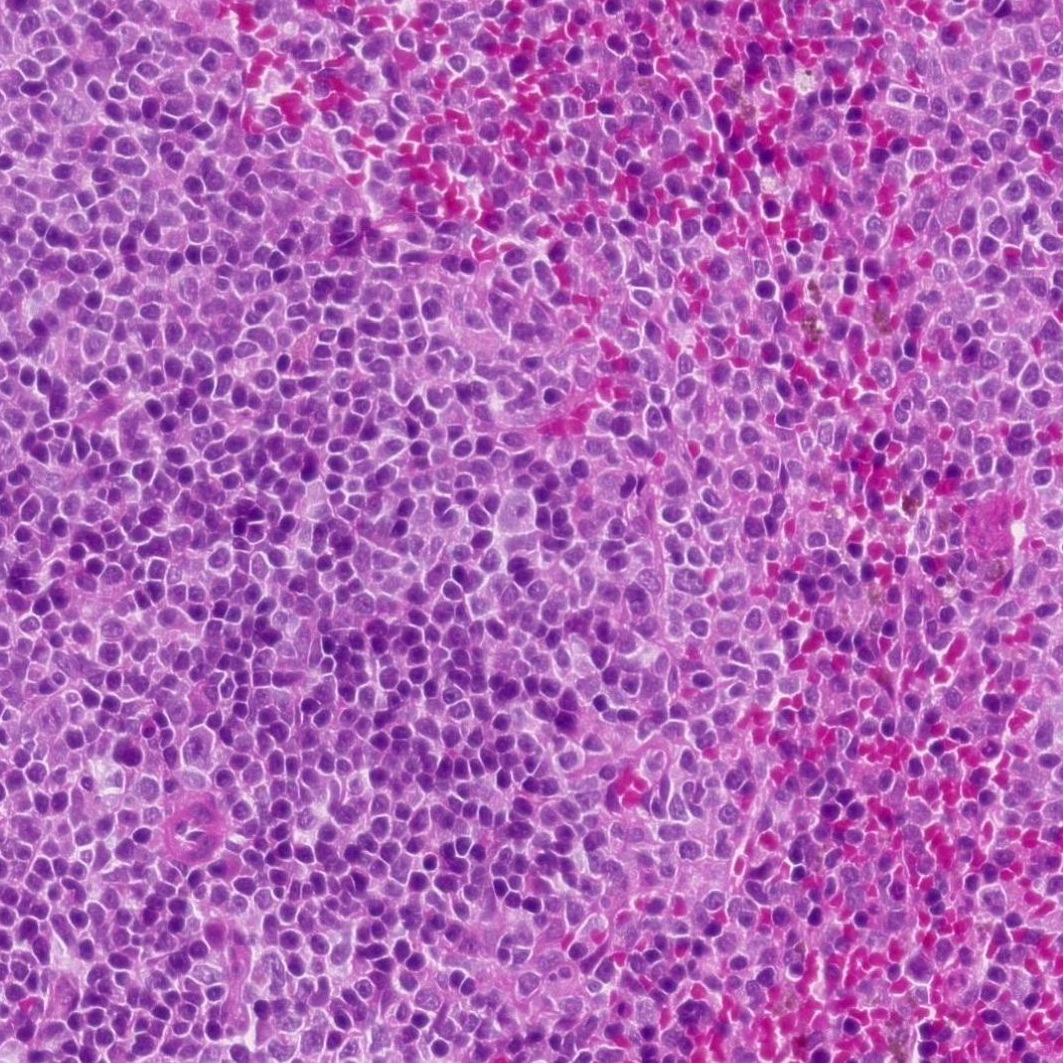

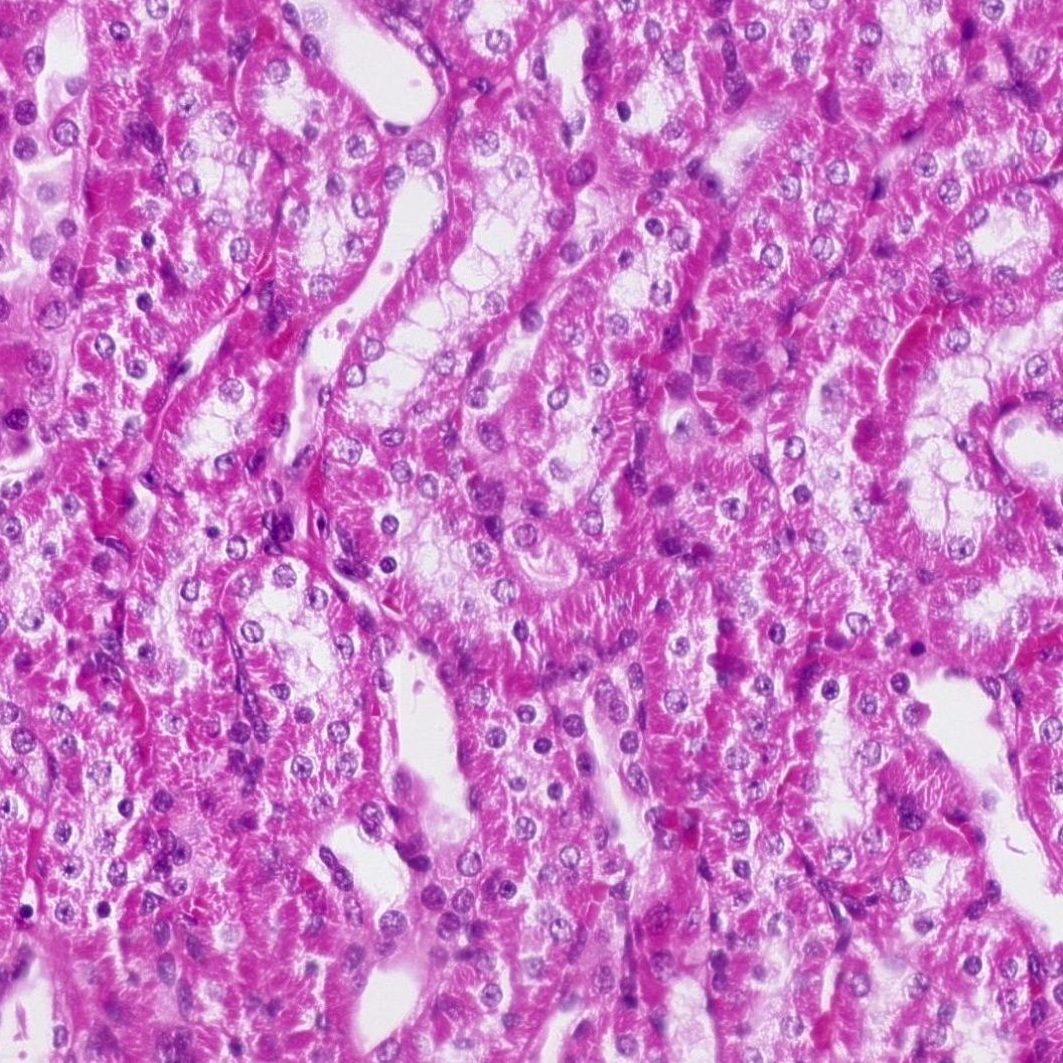


**Liver**

**Spleen**

**Lung**

**Kidney**

**Gnetol (L)**

**(L)**

**Gnetol (H)**

**(H)**

**DEX**

**DOX**

**CON**

**20 μm**

**Figure S11**. Hematoxylin and eosin staining of major organs (liver, spleen, lung, and kidney) from control DOX and gnetol-treated mice.


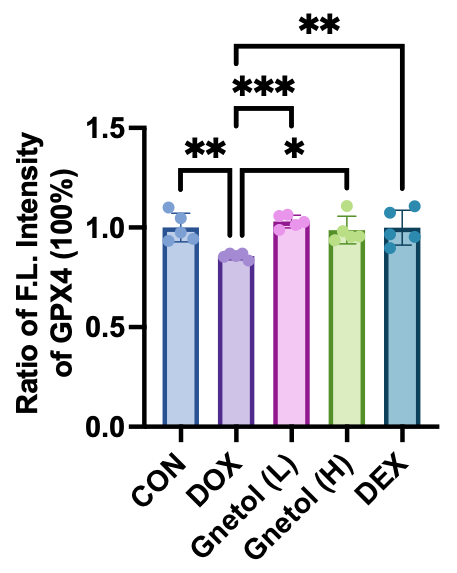


**Gnetol (L)**

**Gnetol (H)**

**DEX**

**DOX**

**CON**


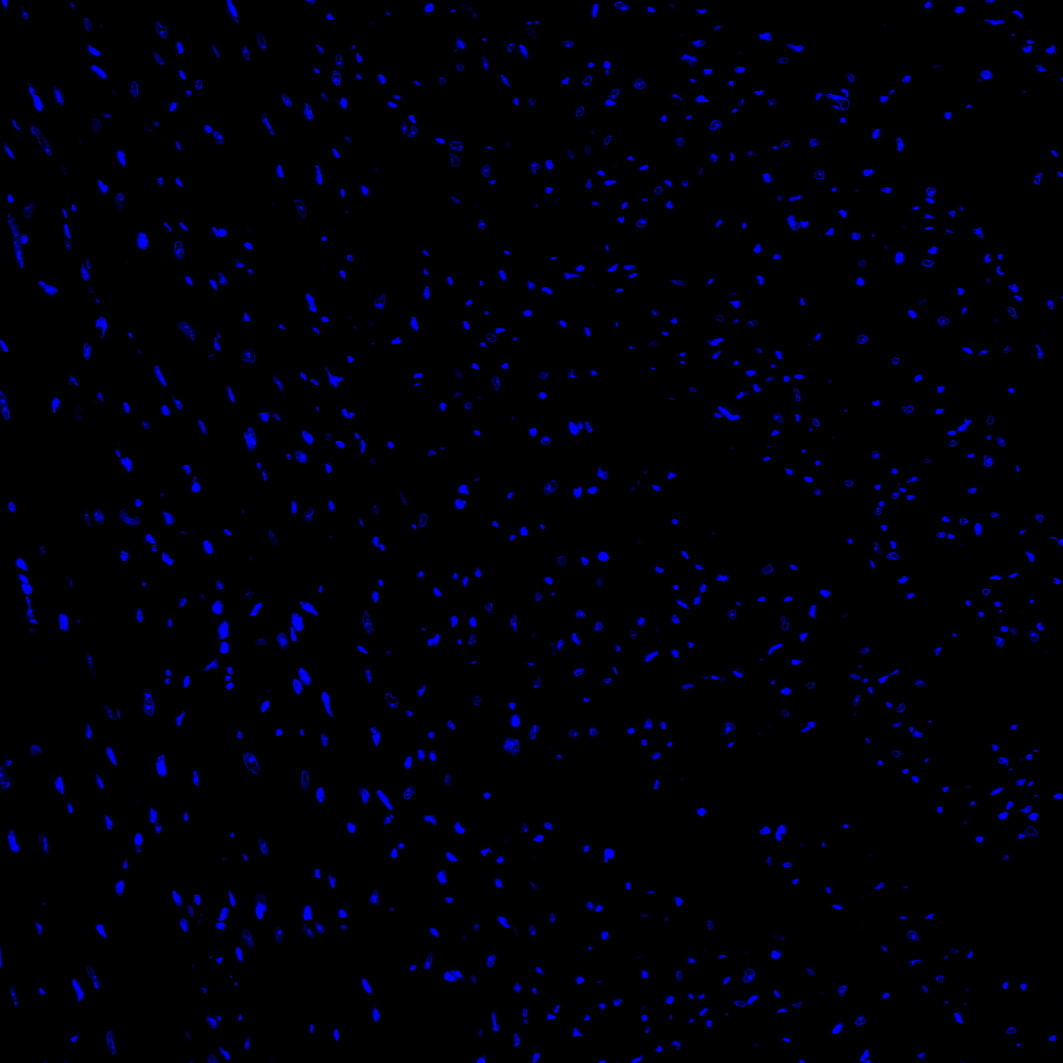

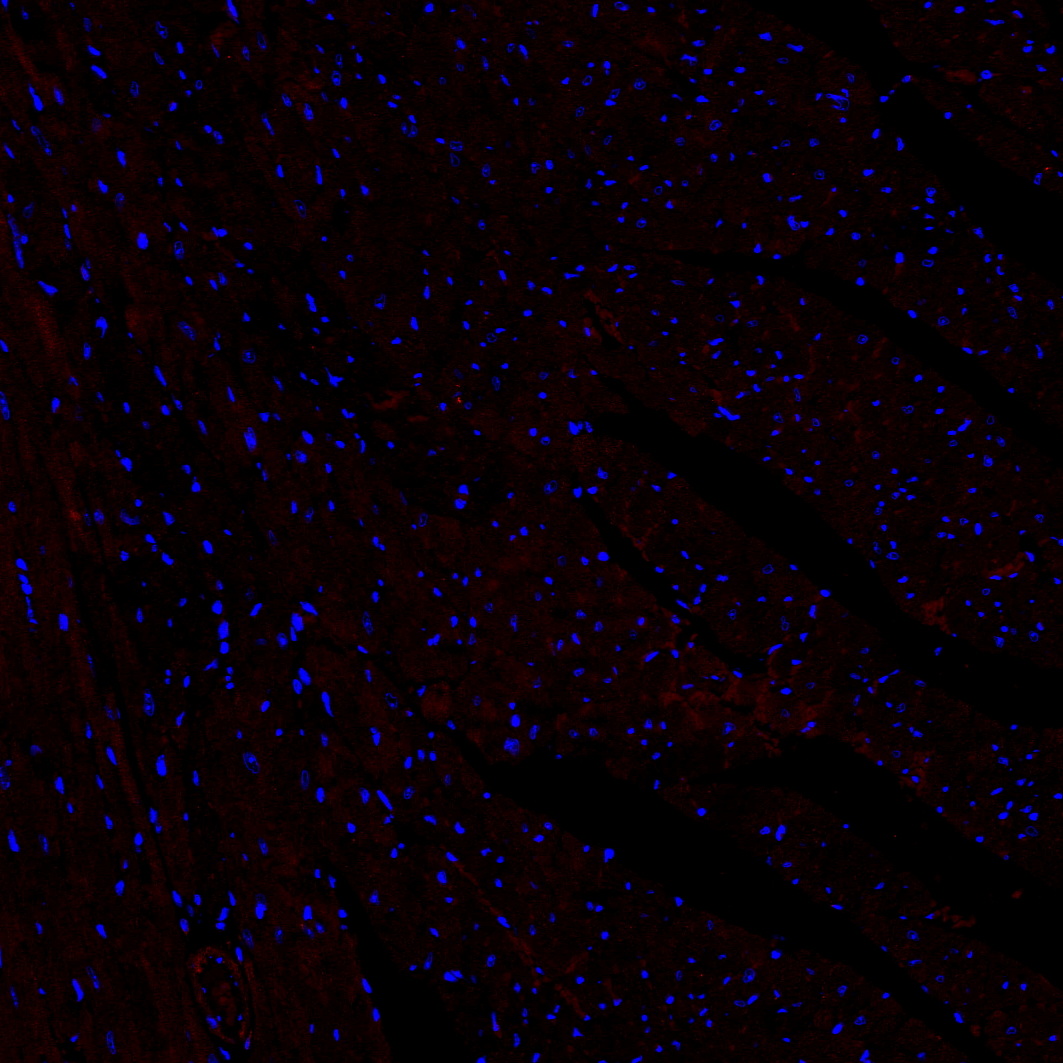

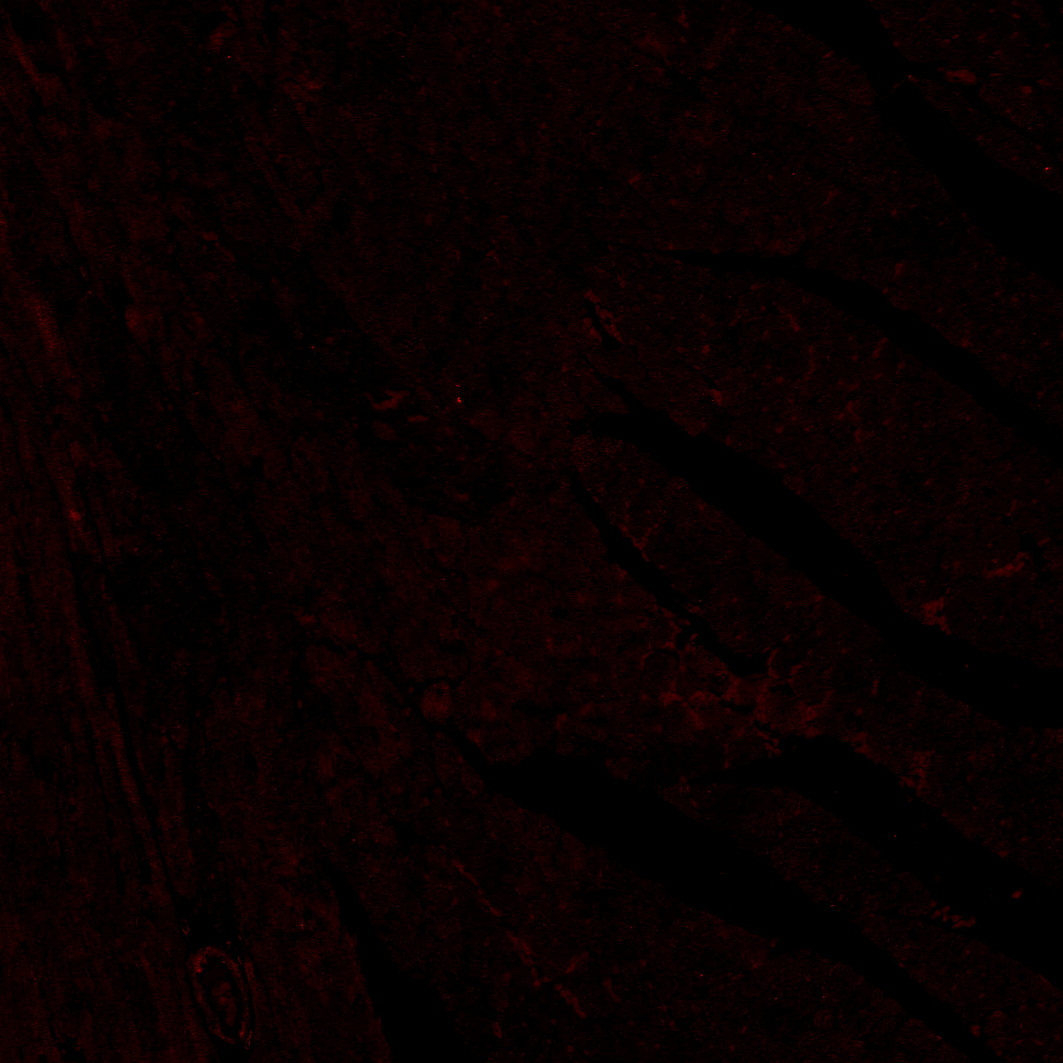

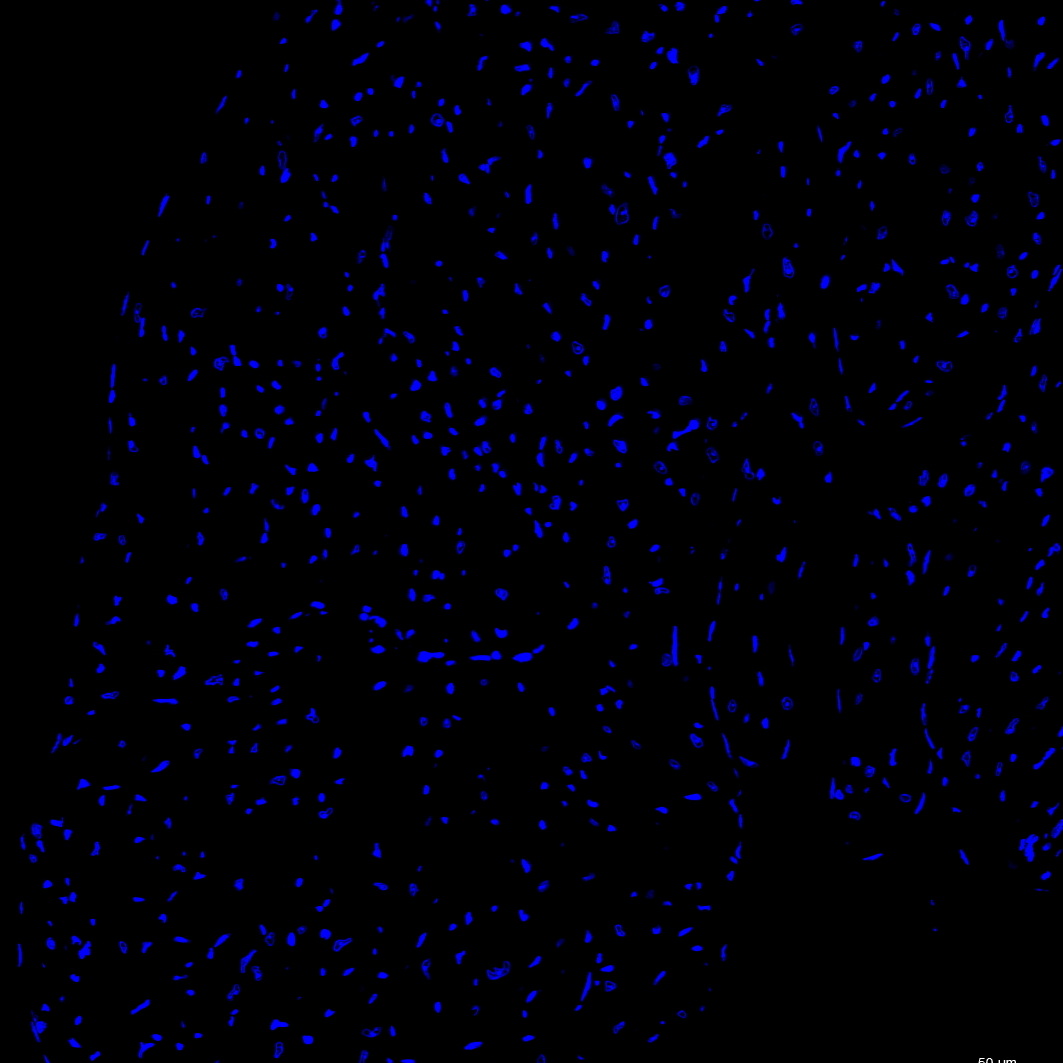

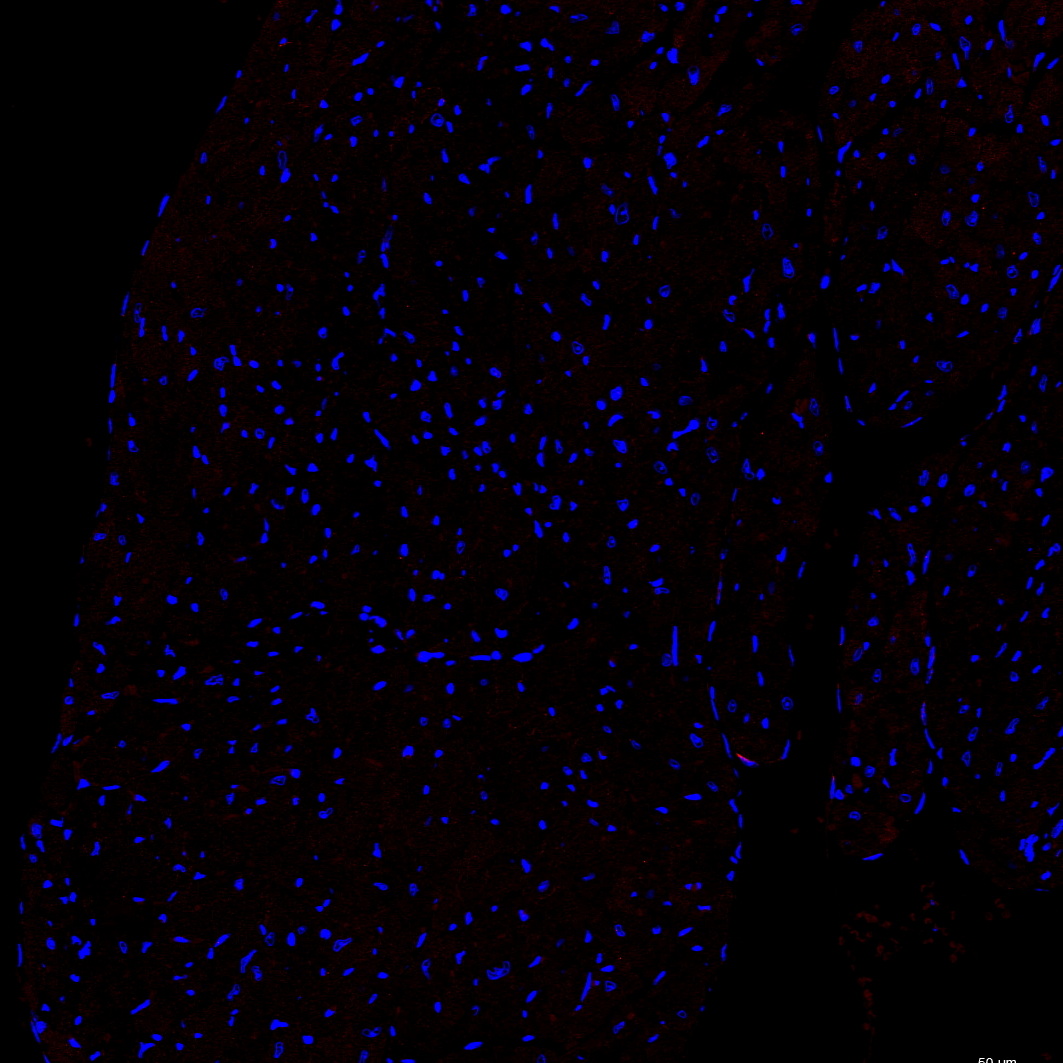

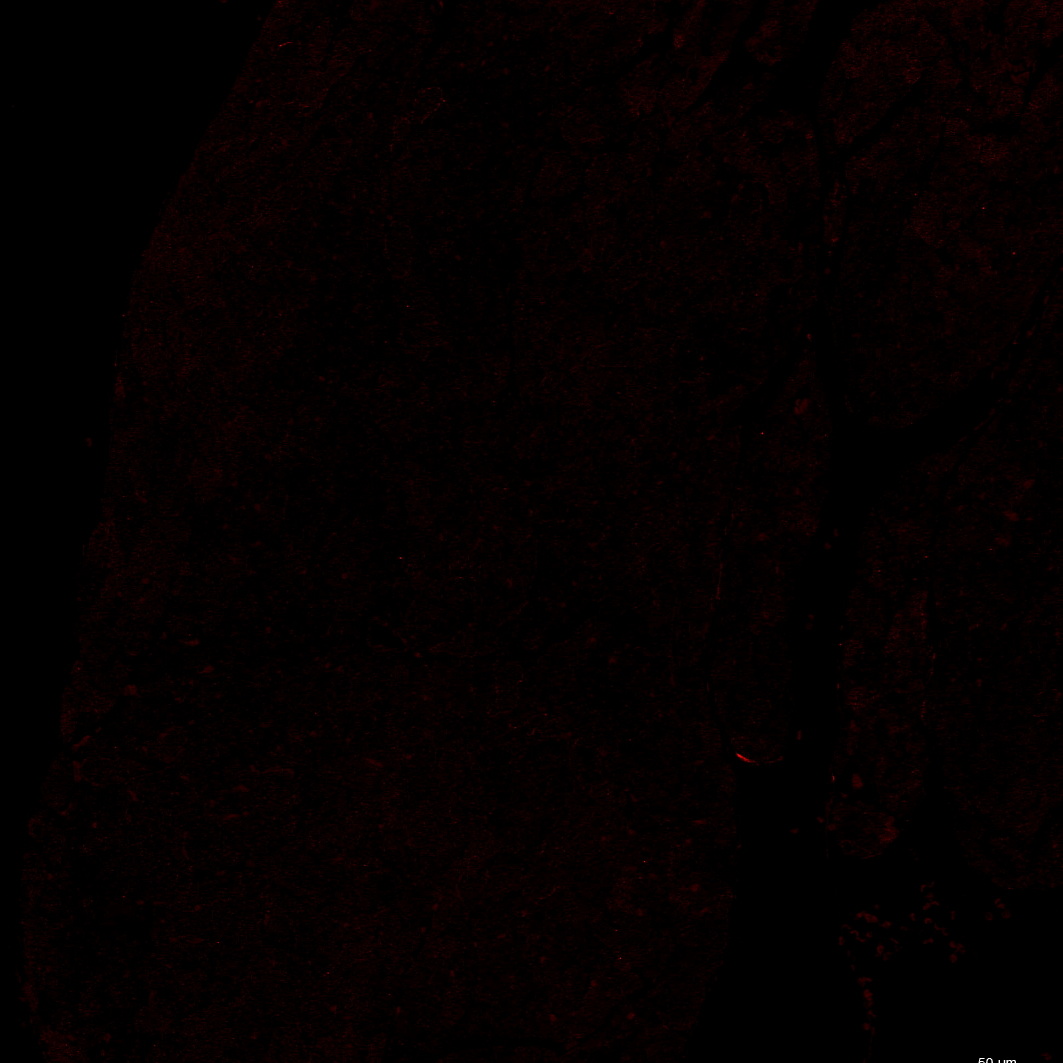

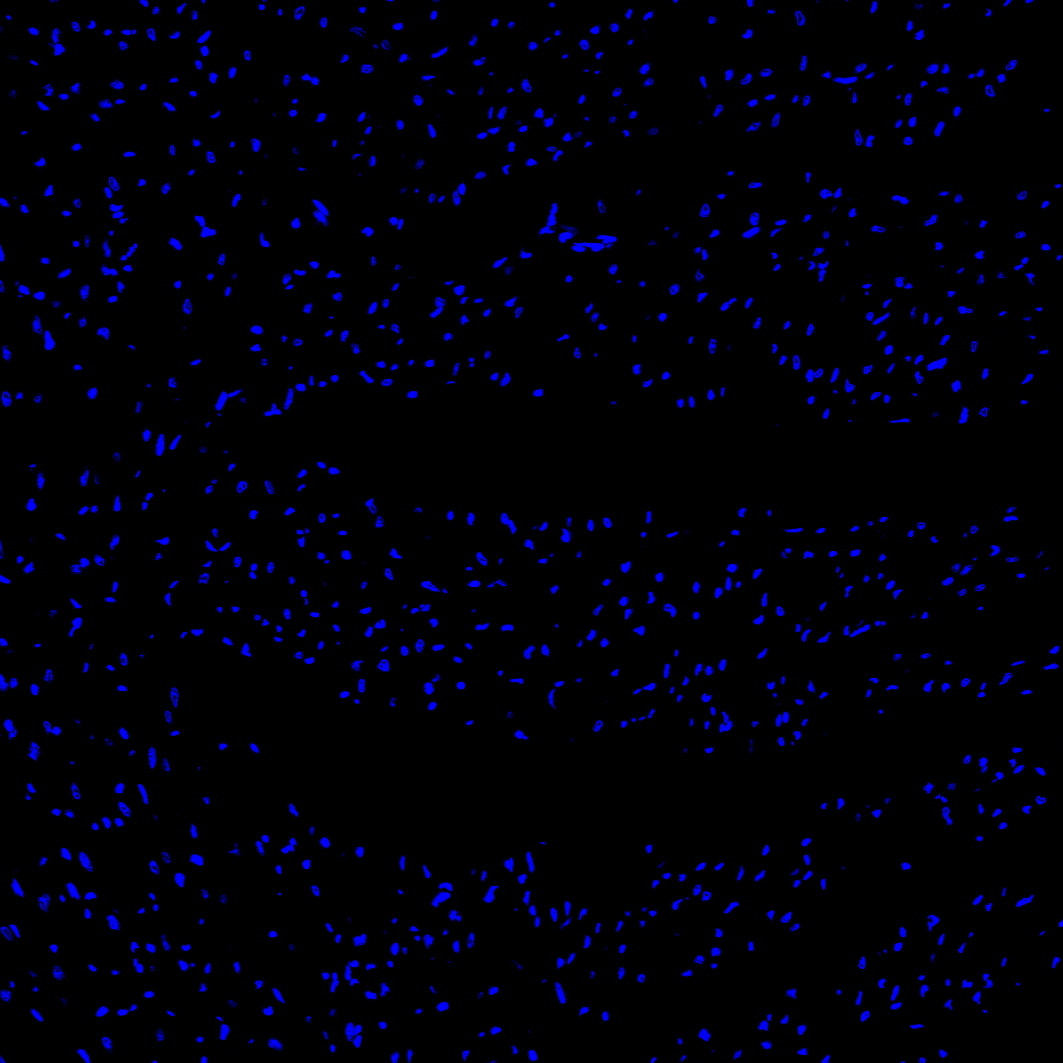

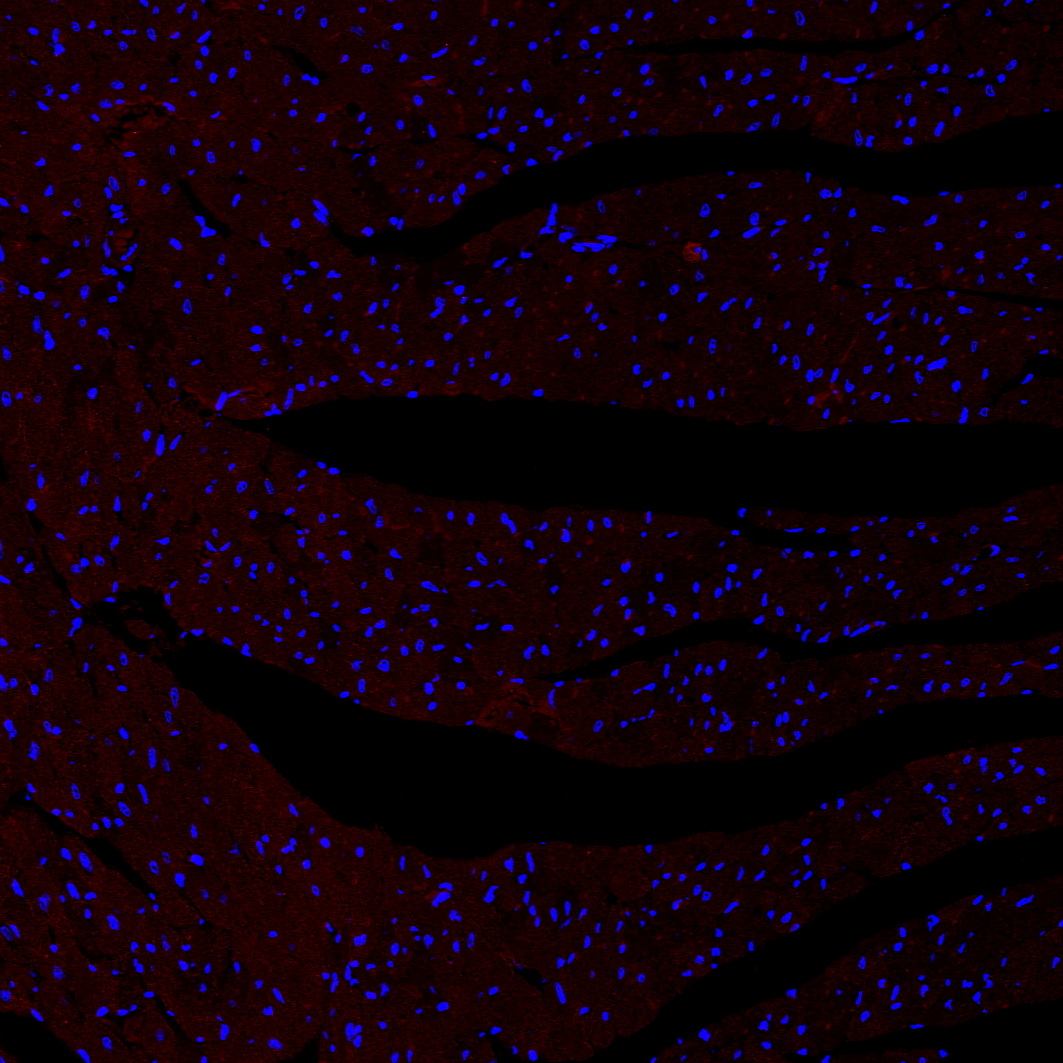

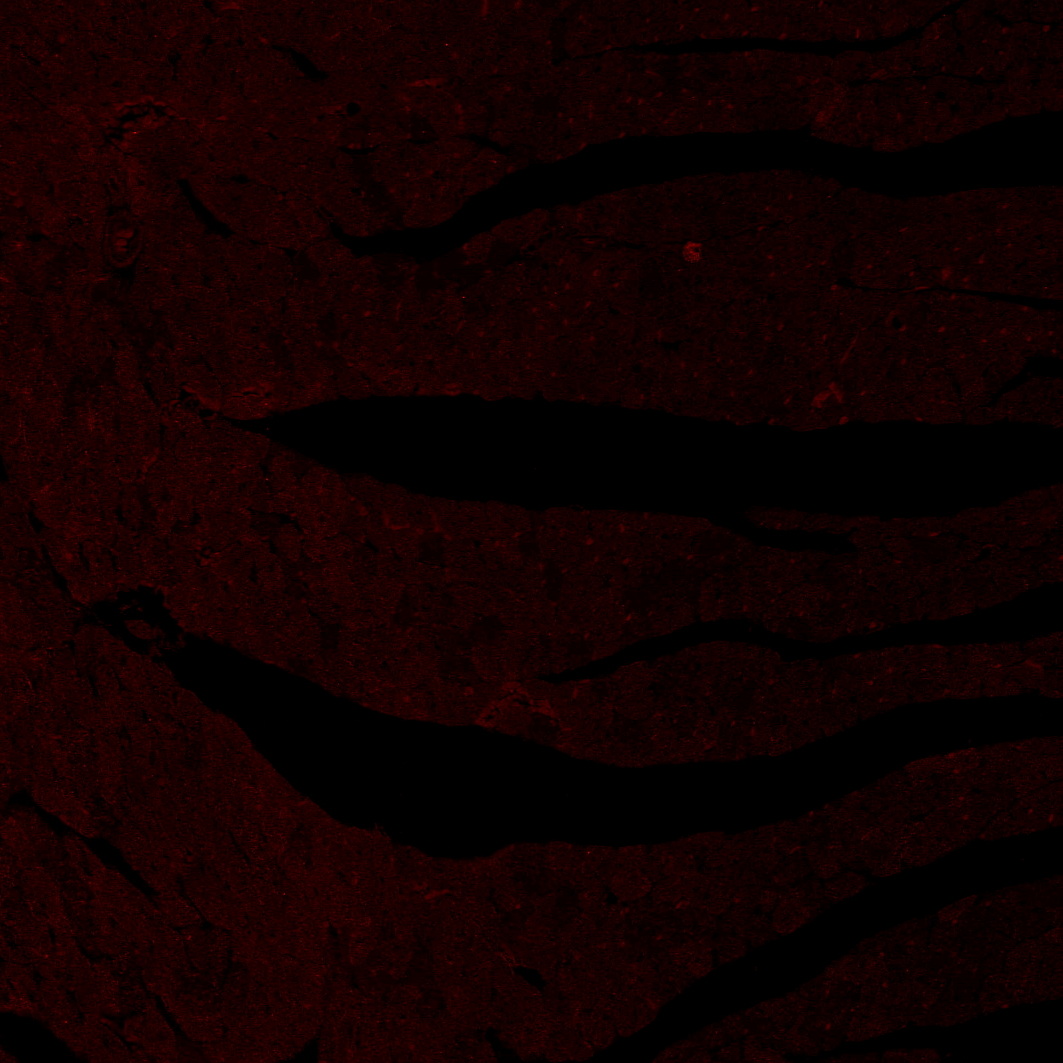

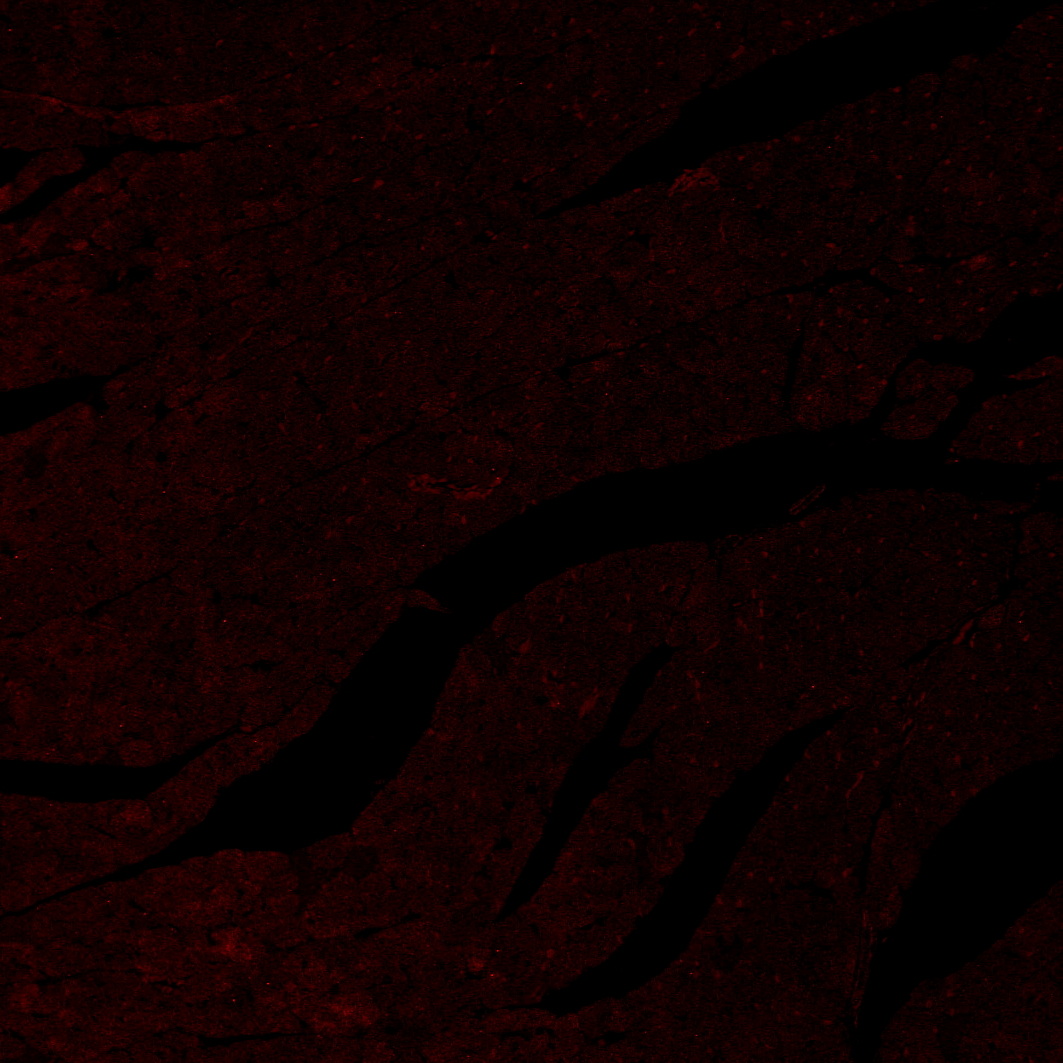

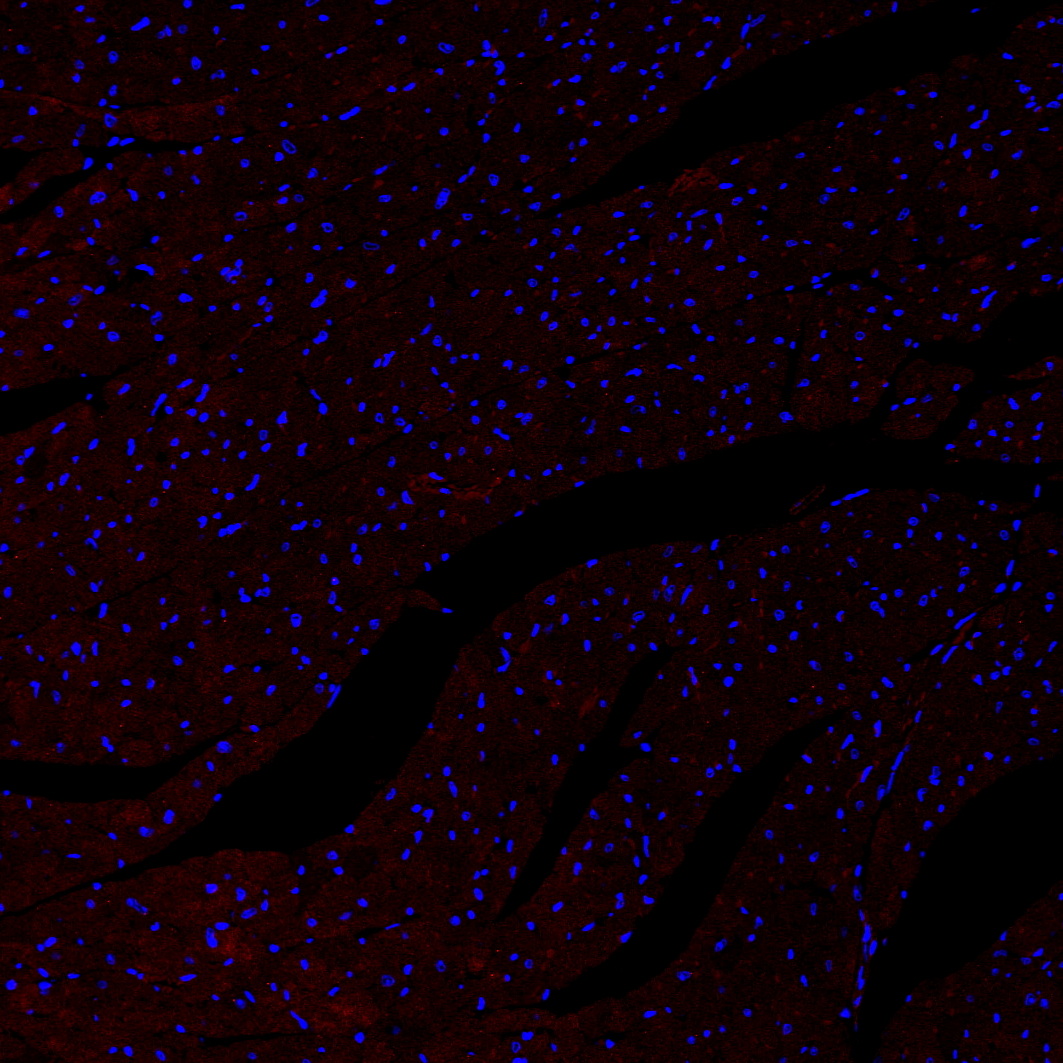

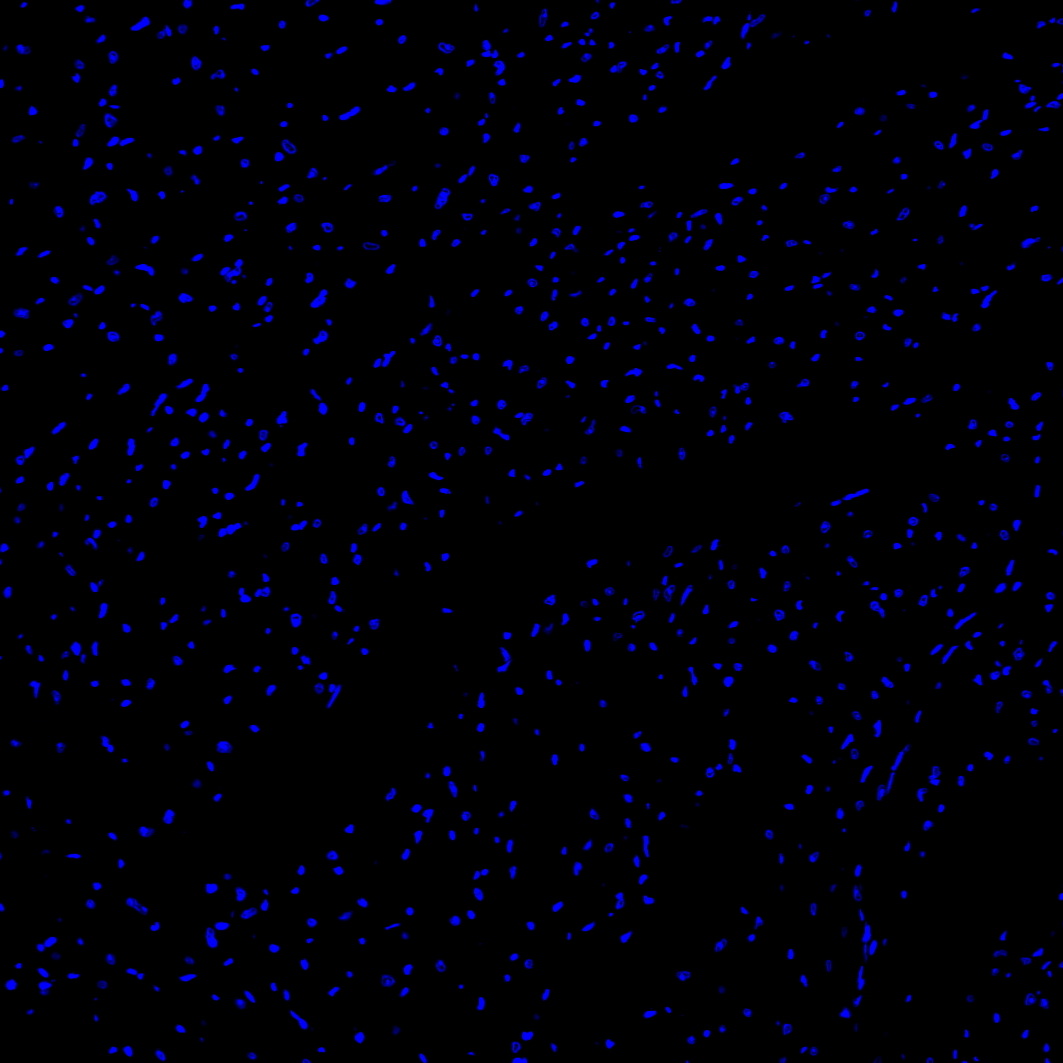

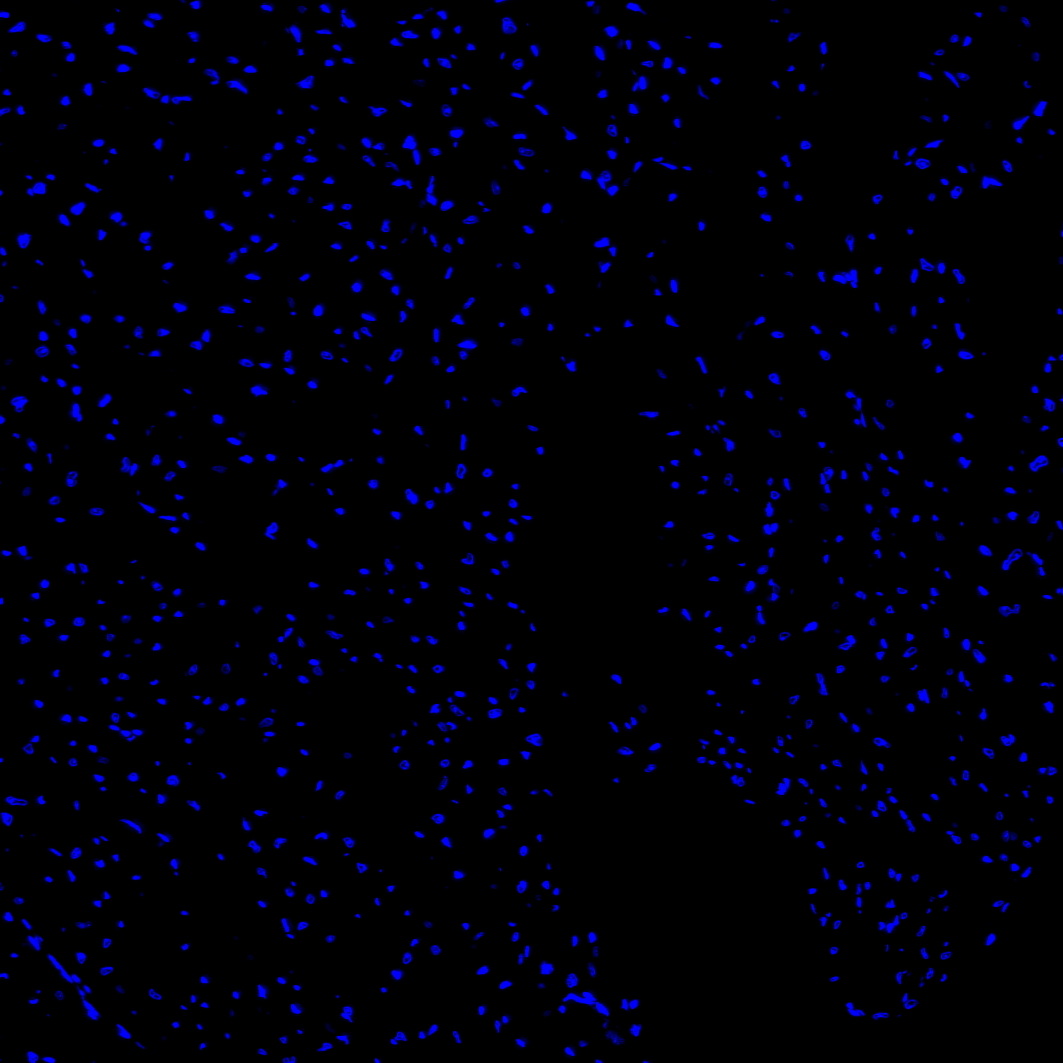

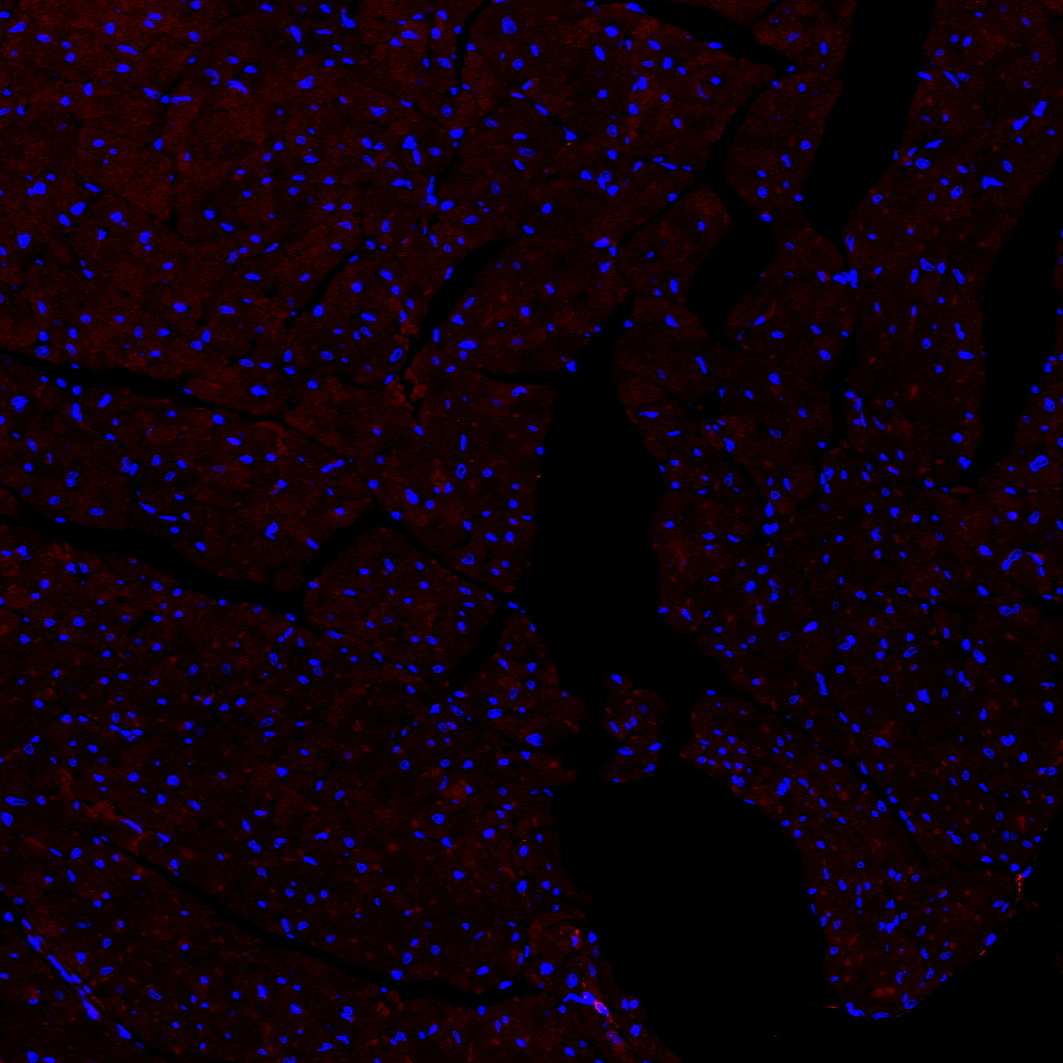

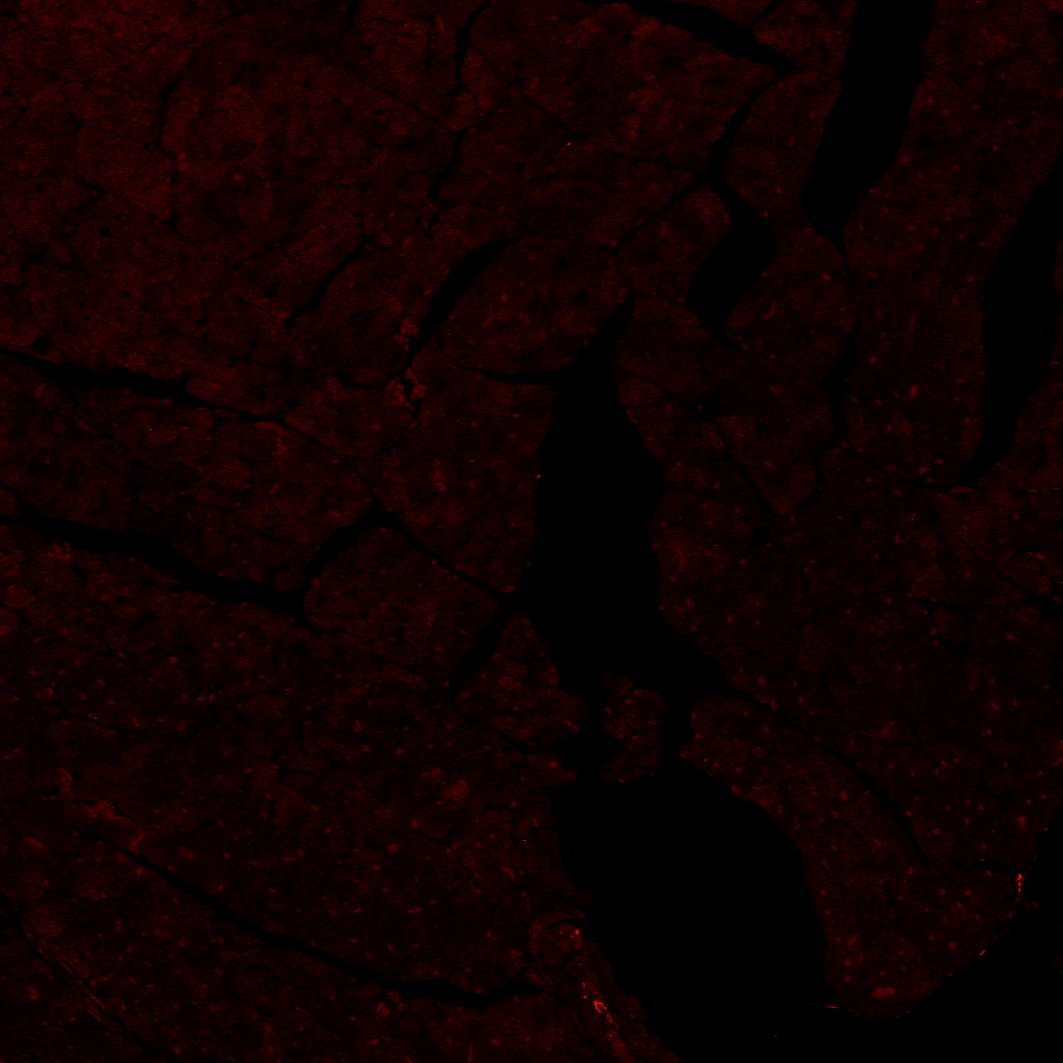


**Merge**

**GPX4**

**DAPI**

**20 μm**

**Figure S12.** Immunofluorescence analysis of GPX4 expression in cardiac tissues.


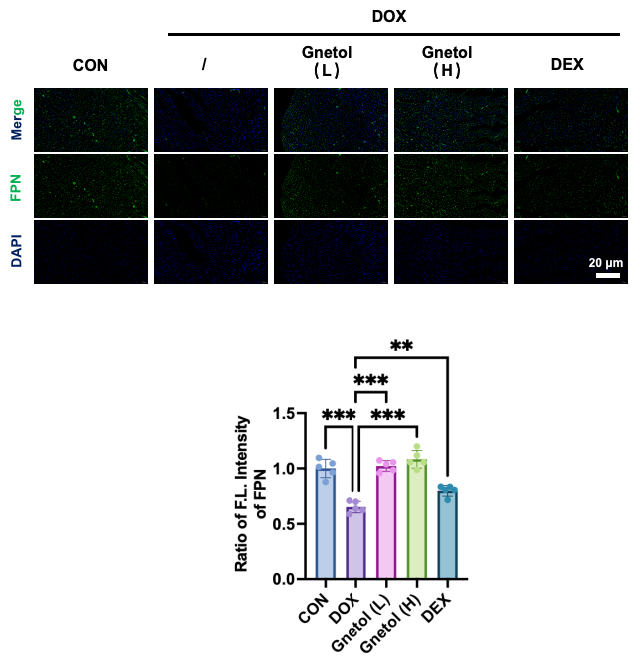


**Figure S13**. Immunofluorescence analysis of FPN1 expression in cardiac tissues.
